# Long non-coding RNAs in the crosstalk between diabetes and colorectal cancer: molecular mechanisms and endocrine pathways

**DOI:** 10.3389/fmolb.2026.1858981

**Published:** 2026-07-10

**Authors:** Lu Zhang, Min Li

**Affiliations:** Clinical Medical College of Acupuncture Moxibustion and Rehabilitation, Guangzhou University of Chinese Medicine, Guangzhou, Guangdong, China

**Keywords:** biomarkers, colorectal cancer, diabetes mellitus, epigenetic regulation, inflammation, long non-coding RNAs, metabolic reprogramming, molecular mechanisms

## Abstract

Colorectal cancer (CRC) and diabetes mellitus are major global health burdens that frequently coexist and share overlapping metabolic and molecular abnormalities. Accumulating epidemiological evidence indicates that diabetes is associated with an increased risk of CRC; however, the underlying mechanisms remain incompletely defined. Long non-coding RNAs (lncRNAs) have recently emerged as important regulators of gene expression and cellular signaling, with critical roles in both metabolic disorders and cancer. In this review, we summarize current evidence on key lncRNAs that are implicated in the shared molecular networks linking diabetes and CRC. These lncRNAs modulate multiple signaling pathways, including PI3K/AKT, Wnt/β-catenin, NF-κB–mediated inflammation, hypoxia-associated signaling, metabolic reprogramming, angiogenesis, and epigenetic regulation. Through interactions with microRNAs (miRNAs), transcription factors, and chromatin-modifying complexes, they influence key biological processes such as insulin signaling, glucose metabolism, epithelial–mesenchymal transition, oxidative stress responses, and tumor progression. Elucidating the roles of lncRNAs provides important insight into the molecular interplay between metabolic dysfunction and colorectal tumorigenesis. Notably, many lncRNAs are detectable in circulating fluids, highlighting their potential as non-invasive biomarkers for early detection and risk assessment in diabetic populations at increased risk of CRC. Furthermore, targeting dysregulated lncRNAs may offer novel therapeutic opportunities to simultaneously modulate metabolic and oncogenic pathways. Overall, integrating lncRNA biology into the study of CRC and diabetes may advance our understanding of disease mechanisms and support the development of improved diagnostic and therapeutic strategies.

## 1 Introduction

Colorectal cancer (CRC) is one of the most common malignancies worldwide and remains a leading cause of cancer-related morbidity and mortality (Wang et al., 2025; Rawla et al., 2019). According to global cancer statistics, CRC ranks among the top three most frequently diagnosed cancers and is a major contributor to cancer-associated deaths (Rawla et al., 2019). The development of CRC is a complex, multistep process driven by the accumulation of genetic mutations, epigenetic alterations, metabolic changes, and dysregulated signaling pathways that collectively promote uncontrolled cellular proliferation, resistance to apoptosis, and tumor progression (Chen Y. et al., 2026). While traditional risk factors such as age, diet, obesity, sedentary lifestyle, and genetic predisposition are well established, growing evidence indicates that metabolic disorders, particularly diabetes mellitus, also contribute significantly to the development and progression of CRC (Chen Y. et al., 2026). Diabetes mellitus, especially type 2 diabetes (T2D), is a chronic metabolic disease characterized by persistent hyperglycemia, insulin resistance, and impaired insulin signaling (Lu et al., 2024). The global prevalence of diabetes has increased dramatically over recent decades, creating a substantial public health burden (Chen S. et al., 2026). Epidemiological studies have consistently demonstrated that patients with diabetes have a significantly higher risk of developing several types of cancer, including CRC (Ali Khan et al., 2020). Moreover, diabetic patients with CRC often exhibit poorer clinical outcomes, increased tumor aggressiveness, and reduced survival compared with non-diabetic individuals (Ali Khan et al., 2020). Although several mechanisms have been proposed to explain the association between diabetes and CRC, including hyperinsulinemia, chronic inflammation, oxidative stress, and metabolic dysregulation, the precise molecular pathways linking these conditions remain incompletely understood (Vekic et al., 2021).

In recent years, non-coding RNAs have emerged as critical regulators of gene expression and cellular homeostasis (Loganathan and Doss, 2023; Diamantopoulos et al., 2025). Among them, lncRNAs, defined as RNA transcripts longer than 200 nucleotides that lack protein-coding potential, have gained considerable attention in biomedical research (Mattick et al., 2023). Initially considered transcriptional noise, lncRNAs are now recognized as key modulators of numerous biological processes, including chromatin remodeling, transcriptional regulation, RNA splicing, and post-transcriptional gene control (Mattick et al., 2023). LncRNAs exert their functions through diverse mechanisms, such as acting as molecular scaffolds, decoys, guides for chromatin-modifying complexes, or competing endogenous RNAs (ceRNAs) that regulate miRNA availability (Mattick et al., 2023). Through these mechanisms, lncRNAs influence critical cellular processes such as proliferation, apoptosis, differentiation, metabolism, and immune responses (Zhang D. et al., 2025). Accumulating evidence indicates that lncRNAs play essential roles in both cancer biology and metabolic diseases (Lin et al., 2020; Shin et al., 2025). In CRC, numerous lncRNAs have been shown to regulate tumor initiation, progression, metastasis, and resistance to therapy by modulating key oncogenic pathways such as PI3K/AKT, Wnt/β-catenin, MAPK/ERK, and NF-κB signaling (Pei et al., 2025; Hashemi et al., 2025). At the same time, many lncRNAs are involved in metabolic regulation, influencing insulin signaling, glucose metabolism, lipid homeostasis, oxidative stress responses, and inflammatory processes associated with diabetes (Tello-Flores et al., 2021; Amine et al., 2025). Importantly, several lncRNAs appear to function at the intersection of these pathways, suggesting that they may act as molecular links between metabolic disorders and tumorigenesis (Tello-Flores et al., 2021).

Recent studies have identified a number of lncRNAs that participate in signaling networks relevant to both CRC and diabetes. These lncRNAs regulate shared molecular mechanisms such as insulin and PI3K/AKT signaling, Wnt/β-catenin activation, inflammatory NF-κB pathways, hypoxia responses mediated by HIF-1α, metabolic reprogramming, angiogenesis, and epigenetic modifications (Wen et al., 2022; Carbone et al., 2026; Fan et al., 2022; Ren et al., 2025; Maharati and Moghbeli, 2023; Yang et al., 2020; Yang Q. et al., 2023). Through interactions with miRNAs, transcription factors, and chromatin-modifying complexes, these molecules influence cellular processes including epithelial–mesenchymal transition (EMT), oxidative stress regulation, autophagy, and immune responses (Feng et al., 2022). The identification of these overlapping regulatory networks provides important insight into how metabolic abnormalities associated with diabetes may promote colorectal tumorigenesis.

Understanding the role of lncRNAs in the molecular crosstalk between diabetes and CRC has significant clinical implications. Many lncRNAs are detectable in circulating blood, plasma, or exosomal fractions, making them promising candidates for non-invasive biomarkers for early detection and disease monitoring. In addition, targeting dysregulated lncRNAs may provide new therapeutic opportunities aimed at modulating both metabolic and oncogenic pathways simultaneously. In this article, we review and synthesize current evidence on lncRNAs that are involved in the shared molecular mechanisms linking CRC and diabetes. We highlight the major signaling pathways regulated by these lncRNAs and discuss their potential roles as biomarkers and therapeutic targets. By integrating findings from cancer biology and metabolic disease research, this work aims to provide a comprehensive overview of the emerging role of lncRNAs as key molecular mediators connecting diabetes with CRC.

## 2 Shared pathophysiological mechanisms linking T2D and colorectal cancer

The epidemiological association between diabetes and CRC has important clinical implications. Extensive evidence from cohort and case–control studies consistently demonstrates that T2D is linked to a moderately increased risk of CRC. Meta-analyses estimate that diabetes confers approximately a 30%–40% higher risk of colon cancer compared with non-diabetic individuals (Lecarpentier et al., 2017). This elevated risk appears to be only partially explained by shared factors such as obesity or age, suggesting that diabetes itself, or its associated metabolic disturbances, may play a causal role in CRC development. Diabetic patients are more frequently diagnosed at advanced disease stages and experience poorer survival outcomes. CRC patients with diabetes exhibit reduced 5-year survival and increased mortality compared with their non-diabetic counterparts (Bella et al., 2013). Collectively, these findings indicate that diabetes not only increases cancer risk but also adversely affects clinical outcomes.

CRC and T2D share multiple lifestyle-related and metabolic risk factors, alongside overlapping molecular pathways. Nearly all features of the metabolic syndrome confer increased susceptibility to both diseases. In particular, obesity and physical inactivity represent well-established risk factors for the development of CRC as well as T2D (Grega et al., 2021; Yu et al., 2022). Excess adiposity contributes to insulin resistance, compensatory hyperinsulinemia, and chronic low-grade inflammation, and individuals with obesity demonstrate a higher incidence of CRC compared with lean counterparts. In the United States and other countries, obesity is estimated to account for a substantial proportion of CRC cases (Bultman, 2017). In contrast, intentional weight reduction and increased levels of physical activity are associated with a decreased incidence of T2D and a reduced risk of CRC (Bultman, 2017). At the cellular level, obesity and diabetes generate a pro-carcinogenic environment through chronic inflammation. Adipose tissue dysfunction in obesity leads to elevated pro-inflammatory cytokines (e.g., TNF-α, IL-6) and activation of NF-κB signaling (Grega et al., 2021). These inflammatory mediators can act systemically and within the colonic mucosa to promote DNA damage, cell proliferation, angiogenesis, and tumorigenesis. In mouse models, a high-fat diet or genetic obesity increases CRC formation through inflammatory pathways (Garcia-Villatoro et al., 2020). Indeed, one review noted that “adipose tissue dysfunction has been associated with accentuated carcinogenesis with chronic subclinical inflammation” (Grega et al., 2021; Ruiz-Malagón et al., 2025). Thus, inflammatory signaling is a shared mechanism linking metabolic disease to colorectal carcinogenesis. Thus, inflammatory signaling is a shared mechanism linking metabolic disease to colorectal carcinogenesis.

Dietary patterns characterized by high consumption of red and processed meats, animal-derived fats, and refined sugars—typical of a Western-style diet—have been associated with an elevated risk of CRC, and also promote insulin resistance and diabetes (Bultman, 2017). In contrast, dietary patterns abundant in fiber, fruits, vegetables, and whole grains are associated with a reduced risk of CRC and contribute to the prevention of T2D (Bultman, 2017).

Tobacco use and excessive alcohol consumption also increase the risk of CRC, partly through the generation of carcinogenic metabolites and the promotion of chronic inflammation. Smoking is additionally associated with a higher risk of T2D, highlighting the shared contribution of behavioral risk factors.

Importantly, even after adjusting for shared factors like obesity and inactivity, patients with diabetes have a higher CRC risk than non-diabetics, suggesting additional biological links (Yu et al., 2022). Likewise, diabetes causes low-grade systemic inflammation that may potentiate tumor growth. Indeed, one review notes that “adipose tissue dysfunction has been associated with accentuated carcinogenesis with chronic subclinical inflammation” (Grega et al., 2021; Ruiz-Malagón et al., 2025). Thus, inflammatory signaling is a shared mechanism linking metabolic disease to colorectal carcinogenesis.

The gut microbiome represents another shared link between diabetes and CRC. Dietary factors, obesity, and diabetes drive alterations in gut microbial composition, leading to dysbiosis. Notably, bacterial taxa enriched in diabetes or obesity can promote colorectal carcinogenesis; for example, *Fusobacterium* species are frequently increased in insulin-resistant individuals and can adhere to the colonic epithelium, triggering pro-inflammatory and pro-survival signaling pathways. Moreover, the gut microbiome associated with high-risk diets generates metabolites that influence cancer susceptibility. Fermentation of dietary fiber by commensal bacteria produces short-chain fatty acids, such as butyrate, which exert tumor-suppressive effects through epigenetic mechanisms, including histone deacetylase (HDAC) inhibition and anti-inflammatory activity (Bultman, 2017). Conversely, diets rich in fat and red meat promote the expansion of bacterial populations that generate secondary bile acids and hydrogen sulfide, both of which induce DNA damage. Collectively, these diet–microbiome interactions influence the risk of both diabetes and CRC. Bultman (2017) emphasized that diet shapes both the composition and functional activity of the gut microbiota, thereby modulating inflammation and influencing CRC development, with microbial metabolites acting as cofactors in epigenetic regulation. In individuals with diabetes, reduced microbial diversity and increased endotoxemia may further promote a tumor-supportive microenvironment. Together, these observations suggest that microbiome dysregulation constitutes a shared mechanistic link between diabetes and colorectal neoplasia (Grega et al., 2021; Bultman, 2017).

At the level of cellular signaling and metabolic regulation, insulin resistance, which is the defining feature of T2D, promotes biological processes that can also support tumor development. Chronic hyperinsulinemia, a compensatory increase in circulating insulin that accompanies insulin resistance, exerts strong mitogenic effects. Through activation of the insulin receptor and hybrid insulin/insulin-like growth factor-1 (IGF-1) receptors, insulin stimulates major oncogenic signaling pathways, including PI3K/AKT/mTOR and RAS/RAF/MEK/ERK, which enhance cellular proliferation and suppress apoptotic processes (Lecarpentier et al., 2017; Bultman, 2017). Insulin also elevates the bioavailability of IGF-1 by downregulating IGF-binding proteins. IGF-1 functions as a powerful growth factor, and evidence from *in vitro* and animal studies demonstrates that IGF-1 signaling promotes colorectal tumor cell proliferation and survival, frequently through activation of the same PI3K/Akt and MAPK signaling pathways. Notably, colorectal tumors often exhibit elevated expression of the IGF-1 receptor as well as the fetal isoform of the insulin receptor, which renders them highly responsive to circulating insulin and IGF signals (Lecarpentier et al., 2017; Yu et al., 2022). Thus, hyperinsulinemia associated with diabetes may directly promote the development of CRC. Consistent with this, epidemiological studies have linked insulin resistance and elevated C-peptide levels, a marker of endogenous insulin secretion, with an increased risk of CRC (Yu et al., 2022). Importantly, the insulin/IGF signaling axis interacts with the canonical Wnt/β-catenin pathway, a key driver of CRC pathogenesis. Mutations in APC, present in the majority of sporadic CRCs, lead to activation of β-catenin–mediated proliferative signaling.

Recent studies indicate that hyperglycemia itself can stimulate Wnt signaling. Elevated glucose levels promote β-catenin acetylation and its translocation to the nucleus, thereby enhancing the expression of Wnt target genes (Lecarpentier et al., 2017). In other words, the metabolic conditions associated with diabetes may potentiate Wnt-driven oncogenic signaling. In contrast, the nuclear co-activator PPARγ, a target of several antidiabetic agents, can inhibit β-catenin signaling, indicating that metabolic therapies may modulate Wnt pathway activity in the colon (Lecarpentier et al., 2017). Hyperglycemia exerts additional pro-tumorigenic effects, as elevated glucose levels promote the generation of reactive oxygen species (ROS) and advanced glycation end-products (AGEs), which induce DNA damage and inflammatory responses. In patients with CRC, elevated levels of AGEs and activation of their receptor (RAGE) have been associated with enhanced tumor invasion and metastasis through ERK/SP1/MMP2 signaling cascades (Yu et al., 2022). These findings indicate that chronic hyperglycemia in diabetes may promote CRC progression through mechanisms independent of insulin signaling.

Diabetes and obesity cause epigenetic and metabolic reprogramming that can facilitate cancer. For example, nutrient excess and metabolic stress in diabetes may alter DNA methylation and histone acetylation patterns in colonic cells, potentially silencing tumor-suppressor genes and activating oncogenes. The same dietary factors that feed tumors can also supply acetyl-CoA and other cofactors for epigenetic enzymes. A landmark review notes that dietary inputs and microbiota-derived metabolites such as butyrate act as co-factors for chromatin modifiers, influencing gene expression in ways that affect CRC risk (Bultman, 2017).

Within this regulatory landscape, lncRNAs have emerged as important molecular mediators linking metabolic disorders and cancer. LncRNAs are RNA transcripts exceeding 200 nucleotides in length that lack protein-coding capacity but serve as important regulators of gene expression (Shan et al., 2020). These molecules interact with chromatin-modifying complexes, transcription factors, and miRNAs, thereby influencing epigenetic regulation, RNA stability, and multiple signaling pathways (Wang and Chang, 2011; Pullen and Rutter, 2014). Aberrant lncRNA expression has been consistently associated with both cancer and metabolic disorders, underscoring their extensive influence on cellular physiology (Xu et al., 2021). Many lncRNAs involved in CRC also regulate glucose homeostasis and insulin signaling. For instance, MALAT1 is overexpressed in multiple cancers, including CRC, as well as in diabetic tissues, where it modulates insulin sensitivity through the NRF2 and JNK pathways (Shan et al., 2020; Arun et al., 2020). These findings indicate substantial crosstalk between CRC and diabetes mediated by lncRNAs. Several shared lncRNAs regulate chromatin architecture and signaling pathways that are critical for both glucose metabolism and tumor progression. They can modulate epigenetic mechanisms, including DNA methylation and histone modifications (Pullen and Rutter, 2014; Zhang et al., 2018), and may also interact with ubiquitination processes (Guha et al., 2024). This study examines 16 lncRNAs implicated in both conditions, with the aim of characterizing their roles in epigenetic regulation and ubiquitination while mapping the convergent molecular pathways they influence. Their frequent detection in accessible biofluids and tissues further underscores their translational potential as biomarkers and therapeutic targets. Investigating these shared lncRNAs may therefore provide valuable insights into common disease mechanisms and support the development of targeted strategies for both metabolic and neoplastic disorders (Wang JJ. et al., 2019).

**FIGURE 1:**
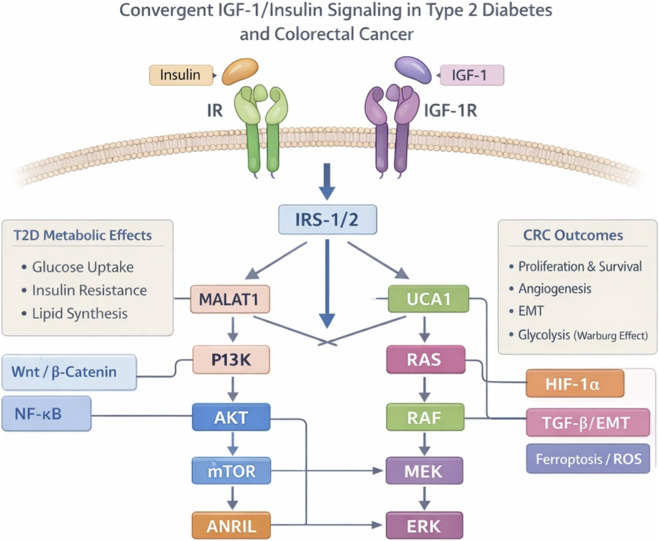
Convergent IGF-1/insulin signaling pathways linking Type 2 Diabetes (T2D) and Colorectal Cancer (CRC). This schematic illustrates how insulin and IGF-1 bind to their respective receptors (IR and IGF-1R) and activate IRS-dependent downstream signaling. Two major pathways—PI3K/AKT/mTOR and RAS/RAF/MEK/ERK (MAPK)—are highlighted due to their central roles in glucose metabolism, proliferation, and survival. In T2D, chronic hyperinsulinemia results in sustained activation of these pathways, contributing to insulin resistance, altered glucose uptake, and increased metabolic stress. In CRC, the same pathways drive tumor growth, cell survival, glycolytic reprogramming (Warburg effect), and enhanced migration. Crosstalk with additional cancer-relevant pathways, including Wnt/β-catenin, NF-κB, and HIF-1α, facilitates angiogenesis, inflammation, and epithelial–mesenchymal transition (EMT). Shown lncRNAs (such as MALAT1, H19, ANRIL, UCA1, ZEB1-AS1) modulate these signaling axes, providing a mechanistic link between diabetic metabolic dysregulation and colorectal tumor progression.

Collectively, Tables 1, 2 summarize the major systemic mechanisms and intracellular signaling pathways that underlie the mechanistic overlap between diabetes and CRC, and Figure 1 depicts the convergent signaling pathways regulated by the IGF-1/insulin axis in CRC and T2D.Building on this framework, the following section provides an overview of lncRNA biogenesis, molecular mechanisms and functional roles, with a particular focus on how lncRNAs integrate metabolic and oncogenic signaling in the context of CRC and T2D.

**TABLE 1:** Systemic mechanisms linking Type 2 Diabetes (T2D) and Colorectal Cancer (CRC).

Systemic mechanism	Role in T2D	Role in CRC	Key molecules/ Pathways
Chronic low-grade inflammation	Elevation of IL-6, TNF-α, CRP; exacerbation of insulin resistance	Promotes proliferation, inhibits apoptosis, enhances tumor invasion	NF-κB, IL-6/STAT3, TNF-α
Hyperinsulinemia and elevated IGF-1	Compensatory response to insulin resistance → increased circulating insulin/IGF-1	IGF-1R activation → PI3K/AKT and MAPK → tumor growth	IGF-1/IGF-1R, IRS-1, PI3K/AKT
Chronic hyperglycemia	Oxidative stress, metabolic dysfunction, epigenetic alterations	Fuels glycolysis, enhances proliferation, supports tumor progression	ROS, HIF-1α, PKM2
Gut microbiome dysbiosis	Metabolic inflammation, reduced SCFAs	Bacterial toxins → mucosal damage → carcinogenesis	LPS/TLR4, SCFAs, pro-carcinogenic bacteria
Obesity and insulin resistance	Increased inflammatory adipokines (leptin), reduced adiponectin	Enhances proliferation and angiogenesis	Leptin/JAK-STAT, Adiponectin/AMPK
Oxidative stress	Endothelial damage, metabolic impairment	DNA mutations, genomic instability	ROS, NRF2, NOX
Epigenetic alterations	Aberrant methylation/acetylation driven by metabolic stress	Regulation of oncogenes and tumor suppressors	DNMTs, HDACs, lncRNAs

**TABLE 2:** Intracellular signaling pathways shared between Type 2 Diabetes (T2D) and Colorectal Cancer (CRC), and corresponding lncRNAs.

Signaling pathway	Role in T2D	Role in CRC	lncRNAs involved	Shared outcomes
PI3K/AKT/mTOR	Impaired insulin signaling and glucose uptake	Tumor growth, survival, drug resistance	MALAT1, H19, ANRIL, KCNQ1OT1	Enhanced metabolism, proliferation, survival
Wnt/β-catenin	Metabolic dysregulation, inflammation	Carcinogenesis, EMT, metastasis	H19, UCA1, ZEB1-AS1	Increased proliferation and invasion
NF-κB	Chronic inflammation, insulin resistance	Tumor inflammation, survival, invasion	MIAT, GAS5 (inhibitory), SNHG4	Systemic and tumor-associated inflammation
HIF-1α/Hypoxia	Glucose metabolism, angiogenesis	Tumor glycolysis, VEGF-mediated angiogenesis	UCA1, SOX2OT	Hypoxia-driven metabolism and angiogenesis
MAPK/ERK	Insulin response, stress signaling	Proliferation, migration	MALAT1, ZEB1-AS1	Enhanced cell growth
TGF-β/EMT	Fibrosis, inflammation	EMT, invasion, metastasis	H19, ZEB1-AS1	Increased migration and phenotypic transition
Ferroptosis regulation	Oxidative stress and cell death modulation	Tumor sensitivity/resistance	GAS5, MIR31HG	ROS and iron homeostasis regulation

## 3 Long non-coding RNAs as molecular integrators in CRC and T2D

### 3.1 Overview of lncRNA biogenesis and characteristics

lncRNAs are a diverse class of RNA transcripts typically longer than 200 nucleotides that lack protein-coding potential. They are transcribed from various regions of the genome, including intergenic regions, antisense strands of protein-coding genes, introns, and enhancer regions (Mattick et al., 2023). Similar to messenger RNAs (mRNAs), most lncRNAs are transcribed by RNA polymerase II and undergo canonical RNA processing steps such as 5 capping, splicing, and polyadenylation (Basu et al., 2023). However, unlike mRNAs, lncRNAs generally exhibit lower expression levels and show more restricted and cell-type-specific expression patterns (Mattick et al., 2023).

LncRNAs can localize to different cellular compartments, including the nucleus, cytoplasm, and occasionally mitochondria. Their subcellular localization is closely linked to their functional roles (Yi et al., 2025). Nuclear lncRNAs frequently participate in transcriptional regulation and chromatin organization, whereas cytoplasmic lncRNAs often regulate mRNA stability, translation, and miRNA activity (Karakas and Ozpolat, 2021). Importantly, lncRNA expression is often tissue-specific and dynamically regulated during development, differentiation, and disease progression (Mehmandar-Oskuie et al., 2024; Cuevas-Diaz Duran et al., 2019). This selective expression pattern suggests that lncRNAs play critical roles in maintaining cellular homeostasis and responding to environmental or metabolic changes.

### 3.2 Molecular mechanisms of lncRNA action

LncRNAs exert their regulatory functions through multiple molecular mechanisms, enabling them to influence gene expression at transcriptional, post-transcriptional, and epigenetic levels (Dai et al., 2025).

One of the most well-characterized mechanisms involves miRNA sponging through the ceRNA network (Dai et al., 2025). In this mechanism, lncRNAs contain binding sites for specific miRNAs, allowing them to sequester these miRNAs and prevent them from repressing their target messenger RNAs (Dai et al., 2025). By acting as molecular sponges, lncRNAs indirectly increase the expression of genes normally suppressed by these miRNAs, thereby influencing pathways involved in cell proliferation, apoptosis, and metabolism (Dai et al., 2025).

Another important mechanism involves chromatin remodeling. Certain lncRNAs interact with chromatin-modifying complexes such as Polycomb repressive complex 2 (PRC2), histone methyltransferases, and HDACs (Costa et al., 2025; Davidovich and Cech, 2015). Through these interactions, lncRNAs can recruit epigenetic regulators to specific genomic loci, altering histone modifications and chromatin accessibility, which ultimately influences gene transcription (Davidovich and Cech, 2015). LncRNAs can also regulate transcription directly by interacting with transcription factors or by forming RNA-DNA hybrid structures at promoter or enhancer regions (Long et al., 2017; Ferrer and Dimitrova, 2024). These interactions may either enhance or suppress the transcription of target genes depending on the context.

In addition, lncRNAs participate in RNA-protein interactions, acting as scaffolds that bring together multiple proteins to form functional regulatory complexes. This scaffolding function enables the coordination of signaling pathways and regulatory networks within the cell. Finally, many lncRNAs contribute to epigenetic modification by guiding chromatin-modifying enzymes to specific genomic regions (Morlando and Fatica, 2018; Jasim et al., 2025). Through these processes, lncRNAs can influence DNA methylation patterns, histone modifications, and chromatin architecture, thereby regulating long-term gene expression programs (Bure et al., 2022; Gaggi et al., 2025).

### 3.3 lncRNAs in metabolic regulation and cancer biology

Increasing evidence suggests that lncRNAs play a crucial role in linking metabolic regulation with cancer development (Shin et al., 2025; Wang K. et al., 2024; Peng et al., 2024). In metabolic disorders such as diabetes mellitus, lncRNAs regulate key processes including glucose metabolism, insulin signaling, lipid metabolism, and mitochondrial function (Abdel-Megee and d, 2026). Dysregulation of these processes contributes to metabolic imbalance and cellular stress.

LncRNAs also participate in the regulation of inflammatory responses (Ghahramani et al., 2025). Chronic inflammation is a common feature of metabolic diseases and is closely associated with tumor initiation and progression. By modulating inflammatory signaling pathways such as NF-κB and cytokine signaling networks, lncRNAs influence the production of inflammatory mediators and immune responses within tissues (Ghahramani et al., 2025).

In cancer biology, lncRNAs regulate multiple tumor-related signaling pathways that control cell proliferation, apoptosis, migration, invasion, and angiogenesis (Ding B. et al., 2025; Zhao S. et al., 2021; Zhang X. et al., 2025). Important pathways influenced by lncRNAs include PI3K/AKT/mTOR signaling, Wnt/β-catenin signaling, and hypoxia-related pathways such as HIF-1α (Hashemi et al., 2025; Maharati and Moghbeli, 2023; Ding B. et al., 2025; Zhao S. et al., 2021; Zhang X. et al., 2025; Braga et al., 2021; Glaviano et al., 2023; Hu et al., 2018). Through these regulatory networks, lncRNAs can function either as oncogenes or tumor suppressors depending on the cellular context.

Notably, the intersection between metabolic regulation and tumor signaling highlights the role of lncRNAs as molecular bridges linking metabolic disorders and cancer. In the context of CRC and diabetes, dysregulated lncRNAs integrate signals from hyperglycemia, oxidative stress, and inflammatory pathways to promote tumor initiation and progression (Table 3). These properties make lncRNAs promising candidates for diagnostic biomarkers and therapeutic targets in metabolic-associated cancers.

**TABLE 3:** Summary of lncRNAs involved in Type 2 Diabetes (T2D) and Colorectal Cancer (CRC) and their associated molecular mechanisms.

LncRNA	Expression in CRC	Expression in T2D	Key targets (miRNAs/Proteins)	Affected pathways	Functional similarities/ Differences
SNHG1	Upregulated, associated with poor prognosis	Upregulated in adipose tissue	miR-449, p53, EZH2	PI3K/AKT, Wnt/β-catenin	Oncogenic in CRC; may promote insulin resistance
GAS5	Downregulated	Low expression in T2D patients	miR-21, IGF1R, mTOR	NF-κB, apoptosis pathways	Protective role in T2D; pro-apoptotic in CRC
UCA1	Upregulated	Increased in various tissues	miR-143, KLF4	TGF-β signaling, cell cycle regulation	Oncogenic in CRC; controversial roles in metabolism
SOX2OT	Upregulated in aggressive tumors	High expression in pancreatic cells	SOX2, p53, NOTCH	Cell cycle, stem cell signaling	Tumor-promoting in CRC; possible regenerative roles in T2D
ZEB1-AS1	Upregulated, supports metastasis	Unknown	miR-200 family, ZEB1	Epithelial-mesenchymal transition, Wnt signaling	Promotes metastasis in CRC; indicated in poor T2D outcomes
MIR31HG	Upregulated	Elevated in diabetic patients	miR-31, PDCD4	MAPK/ERK, inflammatory pathways	Oncogenic role in CRC; involved in inflammation in T2D
HOTAIR	Upregulated, associated with metastasis	Elevated in liver tissue	miR-124, EZH2	PI3K/AKT, transcriptional regulation	Drives CRC metastasis; uncertain T2D link
PANDAR	Upregulated	Unknown	miR-221, c-Myc	NF-κB, p53 pathways	Oncogenic; potential protective effects in T2D unknown
TUG1	Increased in various CRC subtypes	Reduced in adipose tissue	miR-299, p300	Histone modification, cell cycle regulation	Oncogenic in CRC; unclear in T2D
NEAT1	Upregulated in metastatic CRC	Elevated in insulin-resistant models	miR-141, IL-6	Stress response, NF-kB pathways	Pro-oncogenic in CRC; implicated in T2D pathogenesis
LINC00152	Heavily upregulated	Increased in diabetic rat models	miR-149, PTEN	PI3K/AKT, Wnt signaling	Oncogenic role in CRC; no clear protective role in T2D
MALAT1	Upregulated	Elevated in various metabolic tissues	miR-200 family, AXL	Cell cycle, migration pathways	Oncogenic in CRC; roles in metabolic regulation uncertain

## 4 Key lncRNAs implicated in both CRC and T2D

### 4.1 MALAT1

#### 4.1.1 Biological role

Metastasis-Associated Lung Adenocarcinoma Transcript 1 (MALAT1), also known as NEAT2, is an approximately 8-kb nuclear long non-coding RNA located on human chromosome 11q13. It is highly conserved and broadly expressed across multiple tissues. MALAT1 is primarily localized in nuclear speckles, where it functions as a molecular scaffold for RNA–protein complexes and regulates gene expression through transcriptional, epigenetic, and post-transcriptional mechanisms (Arun et al., 2020; Xu et al., 2022).

Originally identified in association with lung adenocarcinoma metastasis, MALAT1 has since been implicated in tumor progression across many cancer types. Although MALAT1 appears largely dispensable for normal development, its aberrant overexpression has been reported in numerous pathological conditions, including CRC and diabetes-associated complications, suggesting the presence of shared pathogenic regulatory mechanisms (Arun et al., 2020; Abdulle et al., 2019).

#### 4.1.2 Mechanisms in colorectal cancer

In CRC, MALAT1 is consistently upregulated and strongly associated with aggressive clinicopathological features, including advanced tumor stage, metastasis, chemotherapy resistance, and poor prognosis. Elevated MALAT1 expression correlates with reduced overall survival and decreased responsiveness to oxaliplatin-based chemotherapy (Xu et al., 2022; Li P. et al., 2017; Amodio et al., 2018).

Mechanistically, MALAT1 promotes CRC progression through multiple oncogenic pathways. A central mechanism involves its function as a ceRNA that sequesters tumor-suppressive miRNAs and thereby derepresses oncogenic targets (Xu et al., 2022). For example, MALAT1 sponges miR-363-3p, leading to increased expression of EZH2, and the catalytic histone methyltransferase of the PRC2 complex. This interaction results in repression of E-cadherin, thereby promoting EMT and resistance to oxaliplatin therapy. MALAT1 also binds miR-218 to enhance metastatic potential and sponges miR-145, resulting in upregulation of SOX9 and increased cellular proliferation (Xu et al., 2022).

Through these regulatory interactions, MALAT1 influences numerous oncogenic signaling networks, including Wnt/β-catenin, Hippo/YAP, SOX9, RUNX2, Snail-mediated EMT, EGF/EGFR, PI3K/AKT/mTOR, p53, and VEGF pathways. Transcriptomic analyses indicate that MALAT1 may regulate the expression of more than 240 genes involved in CRC progression, collectively promoting tumor growth, invasion, angiogenesis, and therapy resistance (Xu et al., 2022). Consistent with these findings, MALAT1 knockdown suppresses CRC cell proliferation and migration, increases E-cadherin expression, and restores chemosensitivity (Xu et al., 2022; Li P. et al., 2017).

At the epigenetic level, MALAT1 interacts directly with the PRC2 complex, including EZH2, SUZ12, and EED, facilitating recruitment of PRC2 to specific genomic loci and promoting H3K27 trimethylation–mediated gene silencing (Xu et al., 2022). In CRC cells, MALAT1-EZH2 complexes repress tumor-suppressor genes and invasion inhibitors such as E-cadherin (Xu et al., 2022; Li P. et al., 2017). MALAT1 can also regulate RNA processing by disrupting the SFPQ/PTBP2 splicing complex, thereby releasing oncogenic PTBP2 and promoting tumor growth (Xu et al., 2022). Through these coordinated epigenetic and post-transcriptional mechanisms, MALAT1 sustains oncogenic signaling networks and contributes to CRC progression.

#### 4.1.3 Mechanisms in diabetes and metabolic dysregulation

Emerging evidence indicates that MALAT1 is also involved in the pathogenesis of diabetes mellitus and its complications. Elevated MALAT1 expression has been observed in diabetic patients and experimental models, particularly in tissues exposed to chronic hyperglycemia, including the retina, kidney, heart, liver, and vascular endothelium (Abdulle et al., 2019; Huang et al., 2020).

Functionally, MALAT1 contributes to diabetic pathology primarily by promoting inflammation, apoptosis, and metabolic dysregulation. Under high-glucose conditions, MALAT1 enhances the expression of pro-inflammatory cytokines such as TNF-α, IL-6, and IL-1β, thereby amplifying inflammatory signaling. In endothelial cells, MALAT1 acts as a sponge for miR-361-3p, relieving repression of SOCS3, a key regulator of the JAK/STAT signaling pathway. Through this mechanism, MALAT1 amplifies inflammatory responses associated with diabetic vascular injury (Huang et al., 2020). Consistently, silencing MALAT1 significantly reduces the secretion of inflammatory cytokines.

In models of diabetic cerebrovascular injury, MALAT1 expression correlates with increased levels of MyD88, IRAK1, and TRAF6, resulting in activation of the NF-κB signaling pathway and enhanced inflammatory damage (Abdulle et al., 2019). These findings support the role of MALAT1 as a pro-inflammatory regulator in diabetes.

Beyond inflammatory signaling, MALAT1 also contributes to metabolic dysfunction in T2D. In obese (ob/ob) mouse models and fatty liver disease, MALAT1 promotes hepatic steatosis and insulin resistance by stabilizing the lipogenic transcription factor SREBP-1c, preventing its degradation and enhancing lipid accumulation. Silencing MALAT1 reduces SREBP-1c expression, improves glucose tolerance, and attenuates hepatic lipid deposition (Yan et al., 2016).

MALAT1 additionally participates in diabetic microvascular complications. In diabetic retinopathy (DR), it functions as a ceRNA for miR-203a-3p, resulting in upregulation of HIF-1α and VEGF, thereby promoting pathological angiogenesis (Perisset et al., 2023). Similar regulatory networks involving miR-15b-5p/TLR4 and miR-144 contribute to apoptosis, fibrosis, and inflammasome activation in diabetic nephropathy (DN) and cardiomyopathy (Yang et al., 2022; Zheng et al., 2025). Collectively, these findings identify MALAT1 as an important regulator of inflammatory and metabolic pathways in diabetes.

#### 4.1.4 Crosstalk between CRC and diabetes

Several signaling pathways regulated by MALAT1 are shared between CRC and diabetes, highlighting its potential role as a molecular bridge between these diseases. One important point of convergence involves inflammatory signaling. In diabetic tissues, MALAT1 activates NF-κB signaling through the MyD88/TRAF6 axis, promoting cytokine production and inflammatory injury (Abdulle et al., 2019; Huang et al., 2020). In cancer cells, MALAT1 can also interact with the NF-κB p65 subunit, modulating inflammatory gene expression and contributing to tumor progression (Gupta et al., 2020).

MALAT1 additionally regulates angiogenesis and EMT in both contexts. In CRC, it promotes VEGF expression through the YAP1/miR-126-5p regulatory axis, while in DR it enhances VEGF signaling via miR-203a-3p sequestration. Likewise, MALAT1 modulates hypoxia responses by stabilizing HIF-1α, a mechanism observed in diabetic retinal cells and potentially relevant to tumor hypoxia within CRC microenvironments (Perisset et al., 2023).

Metabolic signaling represents another shared axis. MALAT1 influences PI3K/AKT/mTOR signaling, which is central to both insulin receptor signaling and cancer cell survival. In CRC, MALAT1 promotes oncogenic signaling through ceRNA-mediated regulation of pathway components (Xu et al., 2022), whereas in metabolic disease it contributes to insulin resistance through stabilization of SREBP-1c and disruption of lipid metabolism (Yan et al., 2016). These overlapping regulatory mechanisms suggest that hyperglycemic and inflammatory environments associated with diabetes may potentiate MALAT1-mediated oncogenic signaling in colorectal tissues.

#### 4.1.5 Immune and inflammatory crosstalk

In addition to metabolic and oncogenic signaling, MALAT1 is increasingly recognized as a regulator of immune-related interactions linking CRC and diabetes. Both diseases are characterized by chronic low-grade inflammation and altered immune cell activity within affected tissues. MALAT1 contributes to this immune dysregulation by modulating cytokine signaling pathways, innate immune activation, and tumor–immune interactions.

In diabetic tissues, MALAT1 enhances inflammatory signaling through NF-κB and JAK/STAT pathways, leading to increased production of cytokines such as TNF-α, IL-6, and IL-1β (Abdulle et al., 2019; Huang et al., 2020). These cytokines contribute to systemic inflammation and insulin resistance while also shaping the tumor-promoting inflammatory microenvironment associated with CRC development. MALAT1-mediated activation of MyD88-dependent signaling further links innate immune responses with metabolic stress. Within the tumor microenvironment, MALAT1 has been reported to influence immune cell recruitment, macrophage polarization, and inflammatory signaling networks, processes that affect tumor growth and immune evasion. Activation of pathways such as NF-κB, PI3K/AKT, and HIF-1α, which are regulated by MALAT1, can modulate cytokine production and angiogenic signaling that shape tumor-associated immune responses.

Although many of these observations derive from studies conducted independently in cancer or metabolic disease models, the convergence of inflammatory and immune regulatory pathways suggests that MALAT1 may contribute to immune-metabolic crosstalk linking diabetes-associated inflammation with colorectal tumorigenesis. Further mechanistic studies in integrated disease models will be necessary to clarify the precise role of MALAT1 in coordinating immune responses at the intersection of metabolic disorders and cancer.

### 4.2 H19

#### 4.2.1 Biological role

H19 is a multifunctional, maternally expressed long non-coding RNA located on chromosome 11p15.5 within an imprinted locus shared with IGF2. This locus plays a critical role in genomic imprinting and epigenetic regulation of growth-related genes. H19 participates in diverse regulatory processes, including transcriptional control, chromatin remodeling, miRNA generation, and ceRNA activity. Through these mechanisms, H19 influences cellular proliferation, metabolism, and developmental pathways (Zhao M. et al., 2021; Hernández-Aguilar et al., 2021; Liao et al., 2023; Rajendran et al., 2024).

Unlike many lncRNAs whose disease associations are inferred indirectly, H19 is supported by substantial clinical and experimental evidence in both CRC and metabolic disorders such as T2D. Its regulatory roles in insulin signaling, inflammatory responses, and tumor-associated pathways position it as an important molecular link between metabolic dysregulation and oncogenesis. Clinical observations indicate that H19 expression is generally upregulated in CRC, although its expression patterns appear sensitive to metabolic status. For example, studies have reported a positive correlation between H19 expression and tumor progression in non-diabetic CRC patients, whereas diabetic CRC cases may show slightly altered expression dynamics, suggesting metabolic context may influence H19-mediated regulation (Zhao M. et al., 2021; Hernández-Aguilar et al., 2021; Liao et al., 2023; Rajendran et al., 2024).

#### 4.2.2 Mechanisms in colorectal cancer

H19 contributes to colorectal tumorigenesis through multiple molecular pathways and exhibits context-dependent functional duality, acting predominantly as an oncogenic regulator but occasionally displaying tumor-suppressive properties under specific conditions. As an oncogenic lncRNA, H19 promotes CRC cell proliferation by interacting with the translation initiation factor eIF4A3, leading to increased expression of CDK4, Cyclin D1, and Cyclin E1, thereby accelerating cell-cycle progression (Zhao M. et al., 2021). H19 also facilitates invasion and metastasis through regulation of key oncogenic signaling pathways.

One well-characterized mechanism involves the H19/miR-29b-3p/progranulin (PGRN) axis. In this pathway, H19 functions as a ceRNA that sequesters miR-29b-3p, resulting in derepression of PGRN. Elevated PGRN activates Wnt/β-catenin signaling, promoting EMT, which is characterized by decreased E-cadherin and increased Vimentin and SNAI1 expression. Concurrently, H19 can activate the Ras/RAF/MEK/ERK pathway, enhancing CRC cell migration and invasion (Chowdhury et al., 2023).

H19 also exerts epigenetic regulatory effects that influence CRC progression. Interaction with the m6A reader HNRNPA2B1 stabilizes RAF-1 mRNA, sustaining activation of the RAF/ERK signaling cascade and promoting oncogenic transcriptional programs. In addition, H19 can recruit chromatin-modifying complexes such as PRC2 or MBD1 to differentially methylated regions within the genome, contributing to silencing of growth-regulatory genes (Chowdhury et al., 2023; Tang et al., 2021).

Through its ceRNA activity, H19 sponges miRNAs including miR-200a and miR-138, leading to upregulation of SIRT1 and subsequent activation of downstream oncogenic pathways such as Rb/E2F and CDK8/β-catenin signaling. Under certain conditions, however, H19 may exert inhibitory effects on tumor growth through its embedded miR-675 or by modulating IGF1R signaling, highlighting the complexity of its regulatory functions (Farooqi et al., 2022; Yang et al., 2021; Zichittella et al., 2023).

Hypoxia represents another key regulatory context for H19 activity. In hypoxic tumor microenvironments, stabilization of HIF-1α leads to transcriptional upregulation of H19. The resulting HIF-1α/H19 signaling axis promotes tumor stemness, chemoresistance, and adaptive metabolic changes. HIF-1α-mediated regulation of ubiquitination pathways can also lead to degradation of tumor suppressors while maintaining oncogenic signaling networks, further contributing to CRC progression (Liao et al., 2023; Zeng et al., 2022; Mu et al., 2023; Groulx and Lee, 2002).

#### 4.2.3 Mechanisms in diabetes and metabolic dysregulation

H19 also plays an important role in glucose metabolism, insulin signaling, and inflammatory regulation, all of which are central features of T2D pathophysiology.

One of the most studied regulatory circuits involves the H19/let-7/PI3K/AKT axis. Under diabetic conditions characterized by hyperinsulinemia and insulin resistance, H19 expression is often reduced, leading to increased levels of let-7 miRNAs. Elevated let-7 suppresses several key metabolic genes, including INSR and LPL, thereby impairing insulin signaling and lipid metabolism (Zhao M. et al., 2021; Nie et al., 2016).

In healthy muscle tissue, insulin stimulation can induce let-7-mediated degradation of H19, forming a feedback mechanism that prevents excessive glucose uptake. Insulin signaling can also suppress H19 expression through PI3K/AKT-dependent phosphorylation of the RNA-binding protein KSRP, which promotes maturation of let-7 while destabilizing H19 transcripts. This regulatory loop highlights a critical checkpoint in insulin signaling and glucose homeostasis (Zhao M. et al., 2021; Nie et al., 2016).

Epigenetically, metabolic dysregulation may further influence H19 activity. In conditions of chronic hyperinsulinemia, H19 has been reported to increase expression of DNA methyltransferase 3 (DNMT3), leading to altered methylation patterns at the H19/IGF2 imprinting control region. These epigenetic changes can disrupt normal growth-factor regulation and may contribute to metabolic complications and cancer susceptibility (Liao et al., 2023). Through these mechanisms, H19 participates in the broader metabolic disturbances associated with T2D, including insulin resistance, dysregulated lipid metabolism, and chronic inflammation.

#### 4.2.4 Crosstalk between CRC and diabetes

Several signaling networks regulated by H19 intersect between CRC and diabetes, highlighting its potential role as a molecular bridge between metabolic disease and tumorigenesis. First, the PI3K/AKT signaling pathway—central to insulin receptor signaling—is also a key driver of cancer cell proliferation and survival. Dysregulation of the H19/let-7 regulatory axis in metabolic disease can therefore influence oncogenic signaling networks involved in CRC development. Second, H19 participates in epigenetic remodeling and chromatin regulation, processes that can be altered by metabolic stress such as hyperinsulinemia or chronic inflammation. Changes in methylation at the H19/IGF2 locus may simultaneously affect metabolic gene regulation and tumor-associated growth pathways. Finally, the HIF-1α/H19 hypoxia signaling axis represents another important point of convergence. Hypoxia is common in solid tumors but can also arise in metabolically stressed tissues. Activation of this axis promotes angiogenesis, metabolic adaptation, and tumor progression, suggesting that metabolic dysfunction may enhance hypoxia-driven oncogenic signaling.

#### 4.2.5 Immune and inflammatory crosstalk

Chronic inflammation is a shared hallmark of both T2D and CRC, and emerging evidence suggests that H19 contributes to the immune and inflammatory signaling networks connecting these conditions. In metabolic tissues, dysregulation of H19 can influence inflammatory pathways associated with insulin resistance, including modulation of cytokine production and signaling cascades such as NF-κB and PI3K/AKT. Persistent metabolic inflammation promotes systemic cytokine release and oxidative stress, which can create a pro-tumorigenic environment. Within the tumor microenvironment, H19-mediated activation of Wnt/β-catenin, Ras/MAPK, and HIF-1α signaling may indirectly influence immune cell recruitment, macrophage polarization, and inflammatory cytokine production. These processes contribute to tumor progression, immune evasion, and angiogenesis.

Although many mechanistic studies have examined these pathways independently in cancer or metabolic disease, their convergence suggests that H19 may participate in immune-metabolic crosstalk linking diabetes-associated inflammation with colorectal carcinogenesis. Further research using integrated disease models will be required to clarify the precise role of H19 in coordinating immune responses at the intersection of metabolic disorders and cancer.

### 4.3 ANRIL

#### 4.3.1 Biological role

Antisense non-coding RNA in the INK4 locus (ANRIL), transcribed from the CDKN2A/B region on chromosome 9p21, functions as a central epigenetic regulator at the intersection of cell-cycle control, inflammation, and metabolism. It exerts its effects mainly through histone modification–mediated gene silencing and post-transcriptional regulation. Acting as a scaffold for Polycomb repressive complexes (PRC1/PRC2), ANRIL governs chromatin states that determine the balance between cellular proliferation and senescence.

#### 4.3.2 Mechanistic roles in colorectal cancer

In oncogenesis, ANRIL modulates PRC2, a histone methyltransferase composed of EZH2, EED, and SUZ12. It recruits EZH2—the catalytic subunit responsible for H3K27 methylation—to promote H3K27me3, thereby repressing tumor-suppressor genes such as p15^INK4B and p16^INK4A. Concurrently, through interaction with CBX7 (a PRC1 component), ANRIL facilitates H2A-K119 ubiquitination (H2AK119ub) and associates with DNMT3B, reinforcing a repressive chromatin configuration across the CDKN2A/B locus. Such aberrant silencing occurs in approximately 40% of human cancers—including gastrointestinal, ovarian, prostate, and pulmonary malignancies (Congrains et al., 2013; Ma and Hu, 2023; Kong et al., 2018).

Upon DNA damage, ANRIL expression is transcriptionally induced by E2F1, which then recruits PRC2 to silence CDKN2A/B and suppress ARF. This limits p53 stabilization by sustaining MDM2 activity, while concurrently activating CDK4/6–cyclin D, allowing Rb phosphorylation and additional E2F1 release—a feed-forward loop that amplifies ANRIL expression and circumvents p53-dependent cell-cycle arrest (Lin et al., 2021). Furthermore, ANRIL directly interacts with SOX2, enhancing Wnt/β-catenin signaling and promoting tumor growth, invasion, and stemness (Kong et al., 2018).

#### 4.3.3 Mechanistic roles in type 2 diabetes and its complications

Beyond cancer, ANRIL is strongly implicated in T2D susceptibility through both genetic and epigenetic mechanisms. Multiple diabetes-associated SNPs adjacent to the ANRIL locus influence pancreatic-islet activity independently of nearby protein-coding genes. Some risk alleles elevate ANRIL expression with age, impairing insulin secretion and β-cell replication, whereas others diminish expression, producing the opposite metabolic consequence. A key variant within ANRIL exon 2 eliminates a CpG methylation site, disrupting glucose-responsive β-cell proliferation—an effect confirmed in human islets.

Under hyperglycemic conditions, ANRIL expression is increased, promoting VEGF transcription via the PRC2/miR-200b axis. In diabetic mice and retinal endothelial cells, ANRIL silencing markedly reduced VEGF-driven vascular leakage and angiogenesis, confirming a causal role in DR (Kong et al., 2018; Thomas et al., 2017). Mechanistically, ANRIL forms an EZH2–p300 epigenetic complex, which activates VEGF chromatin regions and potentiates pathologic neovascularization, providing a potential therapeutic entry point for diabetic microvascular disease.

#### 4.3.4 Crosstalk and immune-inflammatory links

ANRIL integrates cell-cycle regulation, metabolic stress, and inflammatory signaling across the T2D–CRC continuum. Its modulation of NF-κB and HIF-1α pathways under hyperglycemic or hypoxic conditions links chronic inflammation to angiogenesis and tumor progression. The recurrent activation of Wnt/β-catenin, PI3K/AKT, and PRC2-EZH2 circuits in both diabetic and neoplastic tissues underscores ANRIL’s role as a shared epigenetic hub. Collectively, these data identify ANRIL as both a biomarker and a therapeutic target for diseases driven by the confluence of metabolic dysregulation and malignant transformation, although further *in-vivo* validation is warranted to distinguish direct from correlative effects.

### 4.4 KCNQ1OT1

#### 4.4.1 Biological role

KCNQ1OT1 (KCNQ1 opposite strand/antisense transcript 1) is an imprinted lncRNA located on chromosome 11p15.5 with essential regulatory functions across multiple cellular processes, including proliferation, EMT, apoptosis, autophagy, and inflammation. It acts through epigenetic modification, ubiquitin-dependent protein stabilization, and miRNA sponging, enabling broad control over growth and metabolic signaling pathways. Dysregulated KCNQ1OT1 expression has been implicated in CRC, prostate cancer, T2D, DN, and diabetic cardiomyopathy (DCM). Many of these effects converge on shared pathways, most prominently PI3K/AKT/mTOR, Wnt/β-catenin, NF-κB, Hippo/YAP, RAS/ERK, and TGF-β/Smad3, highlighting its significance as a molecular integrator across oncogenic and metabolic disease states (Cagle et al., 2021; Duan et al., 2020; Lin et al., 2021).

#### 4.4.2 Mechanisms in colorectal cancer

KCNQ1OT1 functions as an oncogenic driver in CRC through diverse ceRNA- and protein-regulatory mechanisms. It acts as a competing endogenous RNA, sponging several tumor-suppressive miRNAs. For example, KCNQ1OT1 binds miR-34a to upregulate Atg4B and enhance chemoresistance; regulates PPP1R1B via miR-760 to promote methotrexate resistance; and facilitates metastasis through the KCNQ1OT1/miR-484/ANKRD36 axis (Cagle et al., 2021; Liu et al., 2024a; Zhan et al., 2023; Xia et al., 2022). Additional pathways include the miR-181a-5p/PCGF2 axis and upregulation of ZNF146 via miR-216b-5p sponging, a mechanism relevant in up to 80% of CRC cases (Zhu et al., 2021; Ferbus et al., 2003; Perez-Oquendo and Gibbons, 2022).

KCNQ1OT1 also contributes to immune evasion by upregulating CD155, the ligand for TIGIT, leading to CD8^+^ T-cell exhaustion. Silencing KCNQ1OT1 restores CD8^+^ T-cell infiltration, IFN-γ production, and antitumor immunity, partly through enhanced glucose uptake and restored metabolic fitness in T cells (Lin et al., 2021; Liu J. et al., 2021).

Post-translationally, KCNQ1OT1 stabilizes hexokinase 2 (HK2) by preventing its ubiquitination, promoting aerobic glycolysis and tumor proliferation. It also sponges miR-30a-5p to increase USP22, a deubiquitinase that stabilizes PD-L1, further supporting immune escape mechanisms (Cagle et al., 2021; Lin et al., 2021; Xian et al., 2021).

Autophagy regulation is another oncogenic mechanism: KCNQ1OT1 elevates LC3 expression, enhancing chemoresistance. Epigenetically, it modulates imprinted genes at the KvDMR locus, including CDKN1C (p57), and correlates with CTNNB1 (β-catenin) activation to drive Wnt-dependent tumor growth (Cagle et al., 2021; Lin et al., 2021). Knockdown studies in HCT116 and SW480 cells show reduced proliferation, G2/M arrest, and downregulation of oncogenic pathways, confirming its role as a pro-tumorigenic factor (Liu et al., 2024b). Polymorphisms such as rs35622507 and promoter methylation patterns further influence its expression, chemoresistance, and tumor behavior (Zhang and Wang, 2022).

#### 4.4.3 Mechanisms in T2D and diabetes-related complications

In metabolic disease, KCNQ1OT1 contributes to diabetes progression and its complications through mechanisms analogous to those observed in cancer, chiefly *via* miRNA sponging and activation of inflammatory/fibrotic pathways. In DCM, KCNQ1OT1 sequesters miR-214-3p, increasing caspase-1 and TGF-β1/Smad3 signaling, thereby enhancing cardiomyocyte apoptosis and fibrosis (Yang et al., 2018). In DN, it binds miR-506-3p and miR-18b-5p, upregulating the adaptor protein SORBS2 and activating NF-κB, promoting oxidative stress and renal injury. Additional sponging of miR-93-5p results in ROCK2 overexpression, excessive extracellular matrix (ECM) deposition, and progressive renal fibrosis (Zhao L. et al., 2021). In DR, KCNQ1OT1 drives disease progression by sponging miR-1470, upregulating EGFR, and promoting angiogenesis. Its regulation of the miR-26a-5p/ITGAV/TGF-β/Smad3 axis also contributes to EMT in diabetic cataracts (Xia et al., 2022).

#### 4.4.4 Crosstalk and immune–inflammatory links

KCNQ1OT1 regulates common molecular circuits in both CRC and diabetes, positioning it as a potential mediator of metabolic–oncogenic crosstalk. Across tissues, it activates PI3K/AKT/mTOR, which drives cell proliferation in CRC and contributes to insulin resistance in diabetes (Duan et al., 2020; Ramasubbu and Devi, 2023). Similarly, TGF-β/Smad3 signaling promotes renal fibrosis in DN and EMT/metastasis in CRC. Upregulation of NF-κB by KCNQ1OT1 supports chronic inflammation in diabetes and immune suppression in CRC (Zhao L. et al., 2021; Xia et al., 2022).

KCNQ1OT1 also enhances Wnt/β-catenin signaling via β-catenin stabilization, accelerating tumor progression while influencing metabolic inflammation. Silencing KCNQ1OT1 reverses oncogenic signaling (e.g., Bcl-2, MMP9, Cyclin D1) and restores epithelial integrity (e.g., E-cadherin), supporting its candidacy as a therapeutic target in diseases driven by shared inflammatory, metabolic, and proliferative pathways (Lin et al., 2021; Cagle et al., 2021).

### 4.5 MIAT

#### 4.5.1 Biological role

Myocardial infarction–associated transcript (MIAT; also “Gomafu”) is a nuclear long non-coding RNA with multiple splicing variants, encoded at chromosome 22q12.1. Initially identified through genetic studies in cardiovascular disease, MIAT has since been recognized as a pleiotropic regulator in cancer, neuropsychiatric, and metabolic disorders. Its primary biological functions include modulation of RNA stability, chromatin regulation, and the formation of complex ceRNA networks controlling stress, proliferation, oxidative damage, and apoptosis (Zeinelabdeen et al., 2024; Liu et al., 2018).

#### 4.5.2 Mechanisms in colorectal cancer

In CRC, MIAT acts predominantly as an oncogenic lncRNA. Its overexpression is consistently observed in CRC tissues and cell lines, correlating with poor prognosis and aggressive clinical behavior (Vienot et al., 2025; Zhang et al., 2017; Ghafouri-Fard and Taheri, 2019). Mechanistically, MIAT functions as a ceRNA, sponging tumor-suppressive miRNAs (notably miR-132). By sequestering miR-132, MIAT upregulates Derlin-1 (a p97 ATPase complex member integral to ER-associated degradation), which enhances tumor cell survival, resistance to apoptosis, and EMT progression. Furthermore, MIAT interacts with miR-532-3p to derepress STC1, a pro-angiogenic and pro-metastatic factor, and leverages the miR-488-3p/IGF1R axis to promote CRC cell proliferation and immune evasion (Rostom et al., 2025; Vienot et al., 2025).

#### 4.5.3 Mechanisms in T2D and diabetes-related complications

MIAT is tightly linked with diabetes pathogenesis and its vascular complications. Under hyperglycemic conditions, MIAT expression increases, in part due to direct transcriptional activation by NF-κB and oncogenic transcription factors c-Myc and OCT4. MIAT competitively binds miR-29b, preventing miR-29b from suppressing its target Sp1, a factor that regulates pro-apoptotic and inflammatory gene expression in DR (Liu et al., 2018). Elevated MIAT thereby upregulates Sp1, intensifying apoptosis and retinal cell injury. Importantly, MIAT can potentiate metabolic inflammation through the MIAT/TXNIP axis, enhancing oxidative stress, while also amplifying the activity of inflammatory mediators (TNF-α, IL-6).

#### 4.5.4 Crosstalk and immune–inflammatory links

MIAT acts as a convergent node for tumor progression and diabetic complications by intersecting key inflammatory and growth signaling pathways. NF-κB signaling both induces and is activated by MIAT, establishing a positive feedback loop that sustains chronic inflammation in metabolic and neoplastic tissue (Zhang et al., 2017). Similarly, c-Myc and OCT4–MIAT interactions reinforce survival and apoptosis resistance. The ceRNA networks (miR-132/Derlin-1, miR-532-3p/STC1, miR-488-3p/IGF1R, miR-29b/Sp1) not only promote CRC progression and metastasis, but also overlap with pathways mediating oxidative injury, abnormal angiogenesis, and cellular apoptosis in diabetes-related complications. These mechanistic intersections underscore MIAT’s capacity to connect metabolic/inflammatory cues with cancerous transformation, positioning it as a promising cross-disease biomarker and potential therapeutic target.

### 4.6 UCA1

#### 4.6.1 Biological role

Urothelial cancer–associated 1 (UCA1) is a multifunctional lncRNA first identified in bladder cancer (Ghafouri-Fard and Taheri, 2019). It produces three transcript isoforms (∼1.4, 2.2, and 2.7 kb), with the 1.4 kb variant being the best characterized (Neve et al., 2018). UCA1 is highly expressed in many malignancies, including CRC, and generally correlates with aggressive phenotypes (Neve et al., 2018; Shi CH. et al., 2019). Functionally, UCA1 acts as an oncogenic regulator by influencing transcription and chromatin structure through interactions with epigenetic modifiers (e.g., EZH2) and by serving as a ceRNA that sponges tumor-suppressive miRNAs. It is increasingly recognized as an important modulator of cellular metabolism and energy balance, linking oncogenic transformation with metabolic reprogramming (Zeinelabdeen et al., 2024; Ghafouri-Fard and Taheri, 2019).

#### 4.6.2 Mechanisms in colorectal cancer

UCA1 is markedly upregulated in CRC tissues and cell lines, with high expression correlating with chemoresistance, metastasis, and poor patient survival (Ghafouri-Fard and Taheri, 2019; Luan et al., 2020). Mechanistically, UCA1 promotes tumor proliferation and invasion through coordinated ceRNA activity and epigenetic regulation. As a ceRNA, UCA1 sequesters miR-143 to relieve repression of targets such as MYO6 and KRAS, thereby enhancing cell proliferation, motility, and epithelial–mesenchymal transition (Neve et al., 2018; Luan et al., 2020); it similarly sponges miR-28-5p and miR-204-5p to upregulate oncogenic proteins including BCL2, RAB22A, HOXB3, and CREB1, conferring survival advantages and drug resistance (Ramli et al., 2021). UCA1 also regulates tumor metabolism by activating the mTOR–STAT3–HK2 axis, which increases glycolysis and ATP production, reinforcing the Warburg effect and supporting rapid tumor growth (Li et al., 2014). In addition, UCA1 interacts with major oncogenic signaling pathways, including Wnt/β catenin, MAPK, PI3K/AKT, JAK/STAT, and NF κB, to further drive malignant progression (Liu Z. et al., 2021). Beyond these post-transcriptional and signaling roles, UCA1 physically associates with chromatin remodelers such as EZH2, recruiting it to the p21^Cip1 and p27^Kip1 promoters to promote H3K27me3 deposition and suppress tumor-suppressor gene expression (Neve et al., 2018). Collectively, these mechanisms establish UCA1 as a key driver of CRC progression and a promising biomarker, supported by evidence that circulating UCA1 levels correlate closely with clinical disease stage (Maeda et al., 2021).

#### 4.6.3 Mechanisms in type 2 diabetes and its complications

In contrast to its upregulation in cancer, UCA1 is generally downregulated in T2D, and reduced expression correlates with insulin resistance and heightened cardiovascular risk (Yuan et al., 2022). Functionally, UCA1 contributes to metabolic homeostasis in insulin-responsive tissues. In skeletal muscle, it forms a ceRNA axis with miR-143-3p and FGF21, and under lipotoxic stress, diminished UCA1 permits miR-143-3p–mediated repression of FGF21, leading to mitochondrial dysfunction, increased ROS production, and decreased ATP generation; restoration of UCA1 or FGF21 reverses these metabolic defects (Huang et al., 2023). In the kidney, UCA1 has been shown to inhibit PI3K/AKT signaling and alleviate DN (Shi CH. et al., 2019). Conversely, in DR, UCA1 is induced by hyperglycemia and promotes pathology by sponging miR-624-3p to upregulate VEGF-C, thereby enhancing endothelial proliferation and neovascularization (Yan et al., 2021). Together, these findings highlight the tissue-specific and context-dependent nature of UCA1, which acts protectively in muscle and kidney but contributes to vascular pathology in the diabetic retina.

#### 4.6.4 Crosstalk and immune–inflammatory links

UCA1 serves as a critical nexus integrating metabolic, inflammatory, and oncogenic signaling pathways, thereby linking diabetes-related metabolism with tumor development. The UCA1/miR-143 axis exemplifies this bidirectional relationship: in CRC, UCA1 sequesters miR-143 to promote glycolytic metabolism and cellular proliferation, whereas in muscle tissue, its suppression permits miR-143-3p to downregulate FGF21 and impair mitochondrial respiration (Luan et al., 2020; Huang et al., 2023). Chronic oxidative stress and activation of NF-κB in diabetes may lead to upregulation of UCA1, potentially creating a feedback loop that initially confers protection against ROS but eventually fosters glycolytic reprogramming, inflammation, and increased cancer risk. Shared signaling pathways, such as PI3K/AKT, JAK/STAT, NF-κB, and Wnt/β-catenin, are involved in both metabolic dysfunction and tumorigenesis (Liu Z. et al., 2021; Xia et al., 2014; Indira and Abhilash, 2013; Maschio et al., 2016; Zhang and Wang, 2020). Furthermore, UCA1 interacts with the epigenetic modifier EZH2, which is implicated in insulin resistance, and facilitates GRK2 ubiquitination via Cbl-c, affecting GPCR and insulin receptor signaling (Ramli et al., 2021; Varney and Benovic, 2024). Through these molecular interactions, UCA1 emerges as a central regulator of energy metabolism, inflammation, angiogenesis, and chromatin remodeling, potentially bridging the pathophysiology of T2D and CRC. Consequently, targeting the UCA1/miR-143 axis may offer a promising approach to disrupt this deleterious metabolic and oncogenic crosstalk.

### 4.7 HNF1A-AS1

#### 4.7.1 Biological role

HNF1A-AS1 is a primate-specific long non-coding RNA transcribed antisense to the HNF1A gene. It is broadly expressed in HNF1A-expressing tissues, including the pancreas, liver, and kidney, and is localized predominantly in the nucleus (Beucher et al., 2022; Sepehri et al., 2022). Dysregulation of HNF1A-AS1 has been reported in multiple cancers, including gastric cancer, hepatocellular carcinoma, glioma, lung cancer, and CRC, often correlating with poor clinical outcomes (Sepehri et al., 2022; Zhang et al., 2022). In several malignancies, including CRC, HNF1A-AS1 functions primarily as an oncogenic lncRNA: it is overexpressed in tumors, promotes cellular proliferation, migration, and metastasis, and is associated with reduced patient survival (Zhang et al., 2022; Fang et al., 2017).

Mechanistically, HNF1A-AS1 operates through multiple regulatory modes. It can function as a molecular scaffold or ceRNA, interacting with chromatin-modifying proteins and miRNAs to influence gene expression. For example, HNF1A-AS1 interacts with the PRC2 component EZH2, affecting histone methylation and transcriptional repression (Sepehri et al., 2022). It can also scaffold PRMT1 and the nuclear receptor PXR, facilitating histone arginine methylation and activation of target genes such as CYP3A4. In addition, HNF1A-AS1 directly binds the HNF1A protein, preventing its ubiquitination and proteasomal degradation. Through these interactions, HNF1A-AS1 integrates transcriptional, epigenetic, and post-translational regulation, thereby influencing cellular metabolism and growth (Wang Y. et al., 2024).

#### 4.7.2 Mechanisms in colorectal cancer

HNF1A-AS1 is significantly upregulated in CRC tissues compared with normal mucosa, and elevated expression is associated with poor prognosis. Functional studies demonstrate that silencing HNF1A-AS1 suppresses CRC cell proliferation, invasion, and metastasis both *in vitro* and *in vivo* (Fang et al., 2017).

Several molecular mechanisms contribute to these oncogenic effects. HNF1A-AS1 frequently functions as a ceRNA regulating tumor-suppressive miRNAs. For example, it sponges miR-34a, leading to increased expression of SIRT1, suppression of the p53 pathway, and activation of Wnt/β-catenin signaling, which promotes EMT and metastatic behavior (Fang et al., 2017). Similarly, HNF1A-AS1 can sponge miR-124, resulting in upregulation of its target MYO6, thereby enhancing CRC cell migration, invasion, and glycolytic metabolism (Guo et al., 2020).

Additional mechanisms involve transcriptional activation of oncogenic pathways. HNF1A-AS1 interacts with the transcription factor PBX3, inducing expression of OTX1, which activates ERK/MAPK signaling and promotes tumor angiogenesis (Wu J. et al., 2020). Collectively, these activities position HNF1A-AS1 as a central regulator of multiple oncogenic pathways, including Wnt/β-catenin, PI3K/AKT, and MAPK signaling, through interactions with miRNAs, transcription factors, and chromatin regulators (Fang et al., 2017; Wu J. et al., 2020; Liu HT. et al., 2020). The net effect is enhanced tumor growth, invasion, angiogenesis, and metastatic potential in CRC.

#### 4.7.3 Mechanisms in diabetes and metabolic regulation

Although HNF1A-AS1 itself has not been extensively studied in diabetes, its sense gene HNF1A is a well-established regulator of glucose metabolism. HNF1A encodes a transcription factor essential for pancreatic β-cell function, insulin secretion, and hepatic glucose regulation. Loss-of-function mutations in HNF1A cause maturity-onset diabetes of the young type 3 (MODY3) and increase susceptibility to T2D (DeForest et al., 2023).

HNF1A controls the expression of numerous metabolic genes, including GLUT2, PKLR, insulin, and enzymes involved in glycosylation (Tudor et al., 2022). Because HNF1A-AS1 directly interacts with and stabilizes the HNF1A protein, it may modulate these metabolic pathways indirectly. For instance, stabilization of HNF1A by HNF1A-AS1 can enhance transcription of HNF1A-dependent genes involved in glucose transport, insulin production, and hepatic metabolism (Wang Y. et al., 2024).

Genetic studies further support this connection. The HNF1A antisense promoter region (HASTER) produces HNF1A-AS1 transcripts in pancreatic β-cells and liver tissue. Experimental deletion of HASTER in mouse models leads to dysregulation of HNF1A expression in β-cells, resulting in impaired insulin secretion and diabetic phenotypes (Beucher et al., 2022). These findings indicate that the HNF1A-AS1 genomic locus is important for maintaining HNF1A expression and glucose homeostasis.

In addition, genetic variants within the HNF1A locus, including antisense regions, have been associated with changes in plasma protein glycosylation and inflammatory signaling, processes closely linked to metabolic disease (Tudor et al., 2022). Together, these observations suggest that HNF1A-AS1 may influence diabetes susceptibility by modulating HNF1A-dependent metabolic networks.

#### 4.7.4 Crosstalk between CRC and diabetes

Several shared metabolic and signaling pathways provide a mechanistic link between CRC and diabetes involving HNF1A-AS1. One key intersection involves metabolic reprogramming. HNF1A-AS1 promotes aerobic glycolysis (the Warburg effect) in cancer cells by sponging miR-124 and increasing MYO6 expression (Guo et al., 2020; Cheng et al., 2022). This metabolic shift resembles the altered glucose utilization observed under hyperglycemic conditions in diabetes, suggesting that diabetic metabolic environments may further amplify HNF1A-AS1-mediated tumor metabolism.

Another shared axis is PI3K/AKT signaling. HNF1A-AS1 activates this pathway in several cancers by sponging miR-30b-3p, thereby increasing PIK3CD expression (Liu HT. et al., 2020). Because PI3K/AKT is central to insulin signaling as well as cancer cell survival and proliferation, dysregulation of this pathway may represent a molecular bridge linking metabolic disease and CRC progression (Cheng et al., 2022).

Similarly, HNF1A-AS1 stimulates Wnt/β-catenin signaling through the miR-34a/SIRT1 axis (Fang et al., 2017), a pathway involved not only in intestinal epithelial homeostasis but also in pancreatic β-cell biology. Additional shared signaling cascades include ERK/MAPK activation *via* OTX1(139), which can interact with insulin/IGF-1 signaling networks. Furthermore, HNF1A-regulated genes involved in glycosylation and inflammatory responses provide another potential intersection between diabetes-related metabolic alterations and CRC progression (Tudor et al., 2022).

#### 4.7.5 Immune and inflammatory crosstalk

Beyond metabolic signaling, emerging evidence suggests that HNF1A-AS1 may also contribute to immune-related interactions between diabetes and CRC, although direct experimental evidence remains limited. Both diseases are characterized by chronic low-grade inflammation and dysregulated immune signaling, and several pathways influenced by HNF1A-AS1 intersect with immune regulatory networks.

For instance, activation of PI3K/AKT and MAPK signaling—two pathways influenced by HNF1A-AS1—can regulate inflammatory cytokine production and immune-cell activation in both metabolic tissues and tumor microenvironments. Similarly, modulation of the p53/SIRT1 axis by HNF1A-AS1 may influence inflammatory stress responses and macrophage polarization, processes implicated in diabetes-associated inflammation and tumor immune evasion.

Alterations in protein glycosylation linked to HNF1A signaling may further affect immune recognition and inflammatory responses. Changes in N-glycan structures can modulate cytokine signaling, immune-cell receptor activity, and systemic inflammation, mechanisms relevant to both metabolic disease and cancer progression (Tudor et al., 2022). In the tumor microenvironment, such modifications may influence immune cell recruitment, angiogenesis, and tumor-associated inflammation.

Taken together, these observations suggest that HNF1A-AS1 may participate in a broader regulatory network linking metabolic dysregulation, inflammatory signaling, and tumor progression. Although most current evidence derives from separate metabolic or cancer studies, the convergence of these pathways supports the hypothesis that HNF1A-AS1 could contribute to immune-metabolic crosstalk underlying the increased risk and aggressiveness of CRC in patients with diabetes.

### 4.8 SNHG4

#### 4.8.1 Biological role

Small nucleolar RNA host gene 4 (SNHG4) is a long non-coding RNA located on chromosome 5q31.2 and belongs to the SNHG family, whose members harbor small nucleolar RNAs (snoRNAs) within their introns. The SNHG family (SNHG1–SNHG22) is evolutionarily conserved and broadly expressed, and their exon-derived transcripts function predominantly as regulatory non-coding RNAs that operate in both the nucleus and cytoplasm. Early observations of elevated SNHG4 expression in irradiated TK6 lymphoblastoid cells suggested that it participates in cellular stress responses and adaptive gene regulation (Chu et al., 2021).

Increasing evidence indicates that SNHG4 is dysregulated in a wide range of human diseases. It is commonly upregulated in malignancies, including gastric, renal, glioblastoma, neuroblastoma, colorectal, osteosarcoma, prostate, and lung cancers, where it often acts as an oncogenic regulator that promotes tumor proliferation, migration, and invasion. In contrast, SNHG4 expression is reduced in several inflammatory or degenerative disorders, such as neonatal pneumonia, DR, acute cerebral infarction, and acute myeloid leukemia (Chu et al., 2021). These divergent expression patterns highlight the context-dependent functions of SNHG4, which may either promote tumorigenesis or exert protective anti-inflammatory effects depending on the biological environment.

Mechanistically, SNHG4 regulates gene expression through multiple molecular processes, including miRNA sponging as a ceRNA, transcriptional and epigenetic regulation, modulation of translational activity, and control of protein stability through ubiquitination-related mechanisms (Chu et al., 2021; Dong et al., 2023). Through these diverse activities, SNHG4 influences pathways governing cell proliferation, apoptosis, inflammation, and oxidative stress, positioning it as a multifunctional regulator of cellular homeostasis.

#### 4.8.2 Mechanisms in colorectal cancer

SNHG4 has emerged as an important oncogenic lncRNA in CRC. Multiple studies report significantly increased SNHG4 expression in CRC tissues and cell lines, and its upregulation correlates with advanced disease stage and aggressive clinicopathological characteristics (Chu et al., 2021; Zhou et al., 2021). Functional experiments demonstrate that SNHG4 knockdown suppresses CRC cell proliferation and induces S-phase cell-cycle arrest, suggesting a central role in tumor cell cycle regulation (Zhou et al., 2021).

One key mechanism involves the SNHG4/miR-590-3p/CDK1 axis. SNHG4 acts as a ceRNA that sequesters miR-590-3p, thereby relieving repression of the cell-cycle kinase CDK1. Increased CDK1 expression promotes the upregulation of cyclin B1 and cyclin A2, facilitating G2/M transition and tumor cell proliferation. *In vivo* experiments further confirm this regulatory network, as SNHG4 silencing suppresses tumor growth while inhibition of miR-590-3p reverses the antiproliferative effects of SNHG4 depletion (Zhou et al., 2021).

SNHG4 also regulates ferroptosis, an iron-dependent form of programmed cell death associated with lipid peroxidation. Through the miR-150-5p/c-Myb/GPX4 axis, SNHG4 sponges miR-150-5p to increase expression of the transcription factor c-Myb, which subsequently represses the ferroptosis inducer CDO1 and enhances expression of GPX4, a key antioxidant enzyme that prevents lipid peroxidation. This pathway inhibits ferroptotic cell death, thereby promoting CRC cell survival and contributing to chemoresistance to oxaliplatin (Zhao et al., 2025).

Another important mechanism involves activation of the Wnt/β-catenin signaling pathway. SNHG4 interacts with the RNA-binding protein TAF15, stabilizing RNF14 mRNA, which encodes a RING-type E3 ubiquitin ligase that enhances Wnt/β-catenin signaling. Increased RNF14 expression promotes CRC cell migration, invasion, and tumor growth (Lv et al., 2023). In addition, SNHG4 may regulate additional miRNA networks analogous to other SNHG family members involved in CRC, such as SNHG14-mediated miR-519b-3p/DDX5 signaling (Wang et al., 2021).

SNHG4 has also been linked to chemotherapy resistance through its regulation of tumor suppressor pathways. Overexpression of SNHG4 reduces PTEN mRNA stability, leading to decreased PTEN protein levels and suppression of ferroptosis, thereby enhancing resistance to oxaliplatin treatment (Zhao et al., 2025). Together, these findings identify SNHG4 as a multifaceted oncogenic regulator in CRC that promotes tumor progression through cell-cycle activation, inhibition of ferroptosis, Wnt signaling activation, and chemoresistance mechanisms.

#### 4.8.3 Mechanisms in type 2 diabetes and metabolic complications

Beyond cancer biology, SNHG4 has been implicated in diabetic complications, where it appears to exert largely protective effects against oxidative stress and inflammation. In DR, SNHG4 expression is significantly reduced in retinal tissues of affected patients. Experimental studies in ARPE-19 retinal cells exposed to high glucose demonstrate that SNHG4 overexpression protects against apoptosis. Mechanistically, SNHG4 functions as a ceRNA for miR-200b, which normally suppresses the antioxidant gene Oxr1 (oxidation resistance 1). By sequestering miR-200b, SNHG4 restores Oxr1 expression and reduces oxidative stress–induced retinal cell death, indicating that the SNHG4/miR-200b/Oxr1 axis plays a protective role in retinal cells exposed to hyperglycemic conditions (Yu et al., 2023). Consistent with this observation, SNHG4 downregulation has been specifically associated with DR but not with diabetes alone, suggesting a role in complication-specific oxidative stress responses (Perisset et al., 2023).

SNHG4 also participates in metabolic regulation in non-alcoholic fatty liver disease (NAFLD), a metabolic disorder frequently associated with insulin resistance and T2D. In hepatocyte models exposed to free fatty acids, SNHG4 expression is suppressed, leading to increased lipid accumulation and inflammatory cytokine production. Restoration of SNHG4 expression alleviates triglyceride accumulation and reduces secretion of TNF-α, IL-1β, and IL-6. These effects are mediated through the SNHG4/miR-34b-5p/XIAP axis, in which SNHG4 sponges miR-34b-5p to increase expression of XIAP, an inhibitor of apoptosis that also modulates inflammatory signaling and lipid metabolism (Ding Y. et al., 2025). Knockdown of SNHG4 exacerbates steatosis and inflammatory responses, whereas inhibition of miR-34b-5p rescues these effects, indicating that SNHG4 contributes to metabolic homeostasis and anti-inflammatory regulation in hepatocytes.

Additional studies demonstrate broader anti-inflammatory roles for SNHG4. In neuronal models, SNHG4 suppresses neuroinflammation through the miR-449c-5p/STAT6 pathway, while its knockdown in microglial cells increases production of pro-inflammatory cytokines such as IL-6 and TNF-α (Chu et al., 2021). These findings suggest that SNHG4 may act as a regulator of chronic low-grade inflammation, a central pathological feature of T2Ds.

#### 4.8.4 Crosstalk between CRC and T2D

The contrasting roles of SNHG4 in cancer and metabolic disorders highlight several shared biological processes linking CRC and T2D, including inflammation, oxidative stress, and ferroptosis. In CRC, SNHG4 promotes tumor progression primarily by inhibiting ferroptosis and activating oncogenic signaling pathways, whereas in metabolic diseases its downregulation is associated with enhanced oxidative damage and inflammatory responses (Ding Y. et al., 2025; Yang X. et al., 2023). These findings suggest that SNHG4 may influence the inflammatory microenvironment in both conditions, potentially contributing to tumor-promoting inflammation in CRC and metabolic inflammation in diabetes.

SNHG4 also participates in redox homeostasis, a process central to both tumor biology and diabetic tissue injury. In DR, SNHG4 upregulates Oxr1 through miR-200b sequestration, thereby reducing ROS and protecting retinal cells from oxidative damage (Yu et al., 2023). Although direct regulation of oxidative pathways by SNHG4 has not yet been demonstrated in CRC, cancer cells similarly rely on antioxidant mechanisms to cope with metabolic stress, suggesting that SNHG4-mediated redox regulation may have broader implications across disease contexts.

Ferroptosis represents another critical point of convergence. In CRC, SNHG4 suppresses ferroptotic cell death through the miR-150-5p/c-Myb/GPX4 pathway, enabling tumor survival and resistance to chemotherapy (Zhao et al., 2025). Conversely, GPX4 downregulation in diabetic complications contributes to ferroptosis-mediated tissue damage in organs such as the retina and kidney (Li et al., 2023). These opposing roles underscore the context-dependent regulation of ferroptosis, where increased GPX4 activity promotes tumor progression but protects against metabolic tissue injury.

Beyond signaling pathways, SNHG4 may also exert broader regulatory effects through epigenetic modulation and control of protein ubiquitination. For instance, SNHG4 can indirectly regulate the histone methyltransferase EZH2 via the miR-let-7a axis, suggesting a potential role in chromatin remodeling and transcriptional reprogramming (Dong et al., 2023). Moreover, by stabilizing RNF14 mRNA, SNHG4 increases the expression of this E3 ubiquitin ligase, which enhances degradation of inhibitors of the Wnt pathway and sustains oncogenic signaling in CRC (Lv et al., 2023). SNHG4-dependent regulation of protein turnover may also influence the stability of tumor suppressors such as PTEN, further contributing to tumor progression.

These divergent roles present an important therapeutic challenge. Inhibition of SNHG4 may represent a strategy to suppress tumor growth and overcome chemoresistance in CRC, whereas restoration of SNHG4 activity could be beneficial in diabetic complications by reducing oxidative stress and inflammation. Future research should therefore focus on understanding how metabolic conditions such as hyperglycemia regulate SNHG4 expression and whether tissue-specific targeting strategies could exploit its disease-specific functions. Such insights may enable the development of precision therapeutic approaches that address the complex and context-dependent roles of SNHG4 in both cancer and metabolic disease.

#### 4.8.5 Crosstalk and immune/inflammatory links

Emerging evidence suggests that SNHG4 participates in immune and inflammatory regulatory networks that may connect metabolic disorders such as T2D with colorectal tumorigenesis, although direct mechanistic studies linking both diseases remain limited. Chronic inflammation and oxidative stress are central pathogenic features shared by T2Ds and CRC, and SNHG4 appears to influence several molecular pathways involved in these processes.

In metabolic disorders, particularly DR and fatty liver disease, SNHG4 generally exhibits anti-inflammatory and cytoprotective effects. Reduced SNHG4 expression under hyperglycemic conditions leads to increased oxidative stress and inflammatory cytokine production. For instance, in hepatocyte models of fatty-acid–induced steatosis, decreased SNHG4 expression enhances secretion of pro-inflammatory mediators including TNF-α, IL-1β, and IL-6, while SNHG4 restoration suppresses these cytokines through the SNHG4/miR-34b-5p/XIAP regulatory axis (Ding Y. et al., 2025). Similarly, in retinal cells exposed to high glucose, SNHG4 protects against oxidative injury through the miR-200b/Oxr1 pathway, reducing ROS accumulation and apoptosis (Yu et al., 2023). These findings indicate that SNHG4 can modulate oxidative stress–associated inflammatory signaling, which is a hallmark of diabetic tissue injury.

In contrast, in CRC, SNHG4 tends to promote tumor progression partly by shaping the tumor microenvironment and sustaining survival pathways that counteract stress-induced cell death. By suppressing ferroptosis through the miR-150-5p/c-Myb/GPX4 axis, SNHG4 enhances cellular antioxidant capacity and enables tumor cells to tolerate inflammatory and metabolic stress (Zhao et al., 2025). Elevated GPX4 activity reduces lipid peroxidation and prevents ferroptotic cell death, thereby supporting tumor survival within the inflammatory microenvironment characteristic of CRC. Because ferroptosis is closely linked to immune responses and tumor immunity, SNHG4-mediated ferroptosis suppression may indirectly influence immune cell activity and inflammatory signaling in the tumor milieu.

Additionally, SNHG4 has been implicated in broader inflammatory regulatory circuits involving transcriptional and signaling pathways. Experimental studies in neural and immune cells demonstrate that SNHG4 can regulate inflammatory responses through the miR-449c-5p/STAT6 pathway, which influences cytokine production and macrophage-associated immune signaling (Chu et al., 2021). STAT6 is a key mediator of IL-4/IL-13–dependent immune responses and contributes to macrophage polarization and immune modulation in several pathological contexts, including tumor progression and metabolic inflammation. Although this mechanism has not yet been directly demonstrated in CRC or T2D tissues, it suggests that SNHG4 may influence immune cell behavior and cytokine signaling across different disease environments.

Taken together, available evidence indicates that SNHG4 participates in shared inflammatory and oxidative stress pathways that may contribute to the pathological intersection between diabetes and CRC. In metabolic tissues, reduced SNHG4 expression is associated with enhanced inflammation and oxidative damage, whereas in CRC, increased SNHG4 expression supports tumor survival, ferroptosis resistance, and adaptation to inflammatory stress. This context-dependent regulation highlights the possibility that SNHG4 contributes to the broader network of metabolic-inflammatory signaling linking T2D and cancer, although further experimental studies are required to clarify its precise role in immune modulation within the CRC–T2D axis.

### 4.9 ZEB1-AS1

#### 4.9.1 Biological role

ZEB1-AS1 is an antisense long non-coding RNA transcribed from the promoter region of Zinc Finger E-Box Binding Homeobox 1 (ZEB1) on chromosome 10p11.22. ZEB1 is a well-established transcription factor that functions as a master regulator of EMT, a process critical for tissue remodeling, fibrosis, and tumor metastasis. ZEB1-AS1 positively regulates ZEB1 expression through multiple mechanisms, including epigenetic activation of the ZEB1 promoter and post-transcriptional regulation via ceRNA activity (Su et al., 2017; Jiang et al., 2020; Ghafouri-Fard et al., 2023).

Across multiple cancers, ZEB1-AS1 is frequently overexpressed and functions as an oncogenic lncRNA. Its oncogenic activity is largely mediated through sequestration of tumor-suppressive miRNAs such as miR-205, miR-200c, and miR-141-3p, which normally repress EMT drivers and growth-promoting genes (Ghafouri-Fard et al., 2023; Wu G. et al., 2020). By relieving this repression, ZEB1-AS1 facilitates increased expression of ZEB1 and other downstream oncogenic targets. Consistent with this role, elevated ZEB1-AS1 expression has been reported in several malignancies including colorectal, breast, pancreatic, and glioma cancers, where it contributes to tumor growth, invasion, and therapy resistance.

Mechanistically, ZEB1-AS1 also participates in chromatin remodeling and transcriptional regulation. Experimental studies demonstrate that ZEB1-AS1 can recruit the histone methyltransferase MLL1 to the ZEB1 promoter, increasing H3K4 trimethylation (H3K4me3) and enhancing transcriptional activation of ZEB1 (Su et al., 2017). In addition, ZEB1-AS1 has been linked to recruitment of DNA methyltransferases and HDACs, thereby contributing to epigenetic silencing of epithelial markers such as E-cadherin (CDH1) in cancer cells (Shahryari et al., 2015). Beyond epigenetic regulation, emerging evidence indicates that ZEB1-AS1 may influence protein stability and ubiquitin-dependent signaling pathways, suggesting broader regulatory effects on cellular homeostasis.

Outside of cancer, ZEB1-AS1 is also dysregulated in several metabolic and fibrotic disorders, including DN, pulmonary fibrosis, and atherosclerosis (Ghafouri-Fard et al., 2023). In contrast to its oncogenic role in cancer, ZEB1-AS1 is often downregulated in diabetic tissues, suggesting context-dependent functions in metabolic disease. These contrasting patterns position ZEB1-AS1 as a regulatory molecule capable of influencing EMT, fibrosis, and metabolic stress responses, processes that are relevant to both CRC and T2Ds.

#### 4.9.2 Mechanisms in colorectal cancer

ZEB1-AS1 is consistently upregulated in CRC tissues and cell lines, and increased expression correlates with advanced tumor stage, metastasis, and reduced patient survival (Wu et al., 2023). Functional studies using CRC cell models such as HT-29, HCT116, and SW480 demonstrate that silencing ZEB1-AS1 significantly suppresses tumor cell proliferation and increases sensitivity to 5-fluorouracil chemotherapy, highlighting its contribution to tumor progression and therapy resistance (Ghafouri-Fard et al., 2023).

A major mechanism underlying ZEB1-AS1 activity in CRC involves its role as a ceRNA that regulates oncogenic signaling networks. ZEB1-AS1 sponges tumor-suppressive miRNAs including miR-205 and miR-141-3p, thereby relieving repression of downstream targets such as Yes-associated protein 1 (YAP1), a key effector of the Hippo signaling pathway (Jin and Chen, 2020). Increased YAP1 expression enhances cancer cell proliferation, survival, and resistance to apoptosis. Experimental restoration of YAP1 expression reverses the inhibitory effects of ZEB1-AS1 silencing, supporting the functional significance of the ZEB1-AS1/miR-205/YAP1 axis in CRC progression.

In addition to miRNA sponging, ZEB1-AS1 amplifies major oncogenic signaling pathways implicated in colorectal tumorigenesis. Knockdown experiments reveal that reduced ZEB1-AS1 expression attenuates PI3K/AKT signaling and suppresses Wnt/β-catenin activity, resulting in decreased colony formation and enhanced apoptosis in CRC cells (Ghafouri-Fard et al., 2023). These pathways are well-established drivers of CRC growth and metastasis, and their activation by ZEB1-AS1 suggests that the lncRNA functions as an upstream regulatory hub coordinating multiple proliferative signals.

ZEB1-AS1 also promotes EMT and metastatic dissemination through transcriptional activation of its sense gene ZEB1. Elevated ZEB1 expression suppresses epithelial markers such as E-cadherin while inducing mesenchymal markers, thereby enhancing cellular motility and invasiveness (Otake et al., 2021). Epigenetically, ZEB1-AS1 contributes to sustained ZEB1 expression through MLL1-mediated histone modification, reinforcing EMT transcriptional programs (Su et al., 2017). Together, these mechanisms establish ZEB1-AS1 as a key regulator of EMT activation, oncogenic signaling, and chemoresistance in CRC.

Emerging studies also suggest that ZEB1-AS1 may influence protein stability pathways relevant to tumor progression. Although direct evidence in CRC remains limited, data from other biological systems indicate that ZEB1-AS1 can interact with proteins involved in ubiquitin-dependent degradation. For example, the murine ortholog Zeb1os1 promotes ubiquitination and degradation of the lysosomal channel TRPML1, thereby affecting cellular senescence (Liu et al., 2024b). By analogy, ZEB1-AS1 may regulate stability of key CRC drivers such as β-catenin or ZEB1, potentially by modulating ubiquitin ligases or deubiquitinases (Zhou et al., 2017; Lv et al., 2018; Wang et al., 2018). However, these mechanisms remain largely inferential and require direct experimental validation in CRC models.

#### 4.9.3 Mechanisms in type 2 diabetes and metabolic complications

In contrast to its oncogenic role in CRC, ZEB1-AS1 is typically downregulated in diabetic tissues, and restoration of its expression has been shown to exert protective effects against fibrosis and metabolic injury. Several studies have demonstrated reduced ZEB1-AS1 levels in DN, both in renal tissues from patients and in experimental diabetic mouse models (Song et al., 2019).

Functional experiments indicate that ZEB1-AS1 overexpression in high-glucose–treated renal tubular epithelial cells (HK-2) reduces ECM accumulation and suppresses fibrotic signaling. Mechanistically, this effect is mediated through the ZEB1-AS1/miR-217/MAFB axis. ZEB1-AS1 functions as a molecular sponge for miR-217, thereby increasing expression of the transcription factor MAFB, which inhibits fibrogenic pathways and attenuates renal fibrosis (Song et al., 2019). In diabetic mouse models, ZEB1-AS1 overexpression improves renal function and reduces histological markers of fibrosis, supporting a protective role in diabetic kidney disease.

Similar mechanisms have been described in DCM. Under hyperglycemic conditions, reduced ZEB1-AS1 expression contributes to cardiac fibroblast activation and collagen deposition. Experimental restoration of ZEB1-AS1 suppresses fibroblast proliferation and ECM accumulation via the miR-181c-5p/SIRT1-YAP pathway (Wu et al., 2023). Through sequestration of miR-181c-5p, ZEB1-AS1 increases expression of SIRT1, a deacetylase that regulates metabolic stress responses and modulates YAP signaling. This pathway ultimately reduces myocardial fibrosis and improves cardiac cellular homeostasis.

ZEB1-AS1 has also been implicated in diabetic pulmonary injury, where decreased circulating levels correlate with disease severity. Forced expression of ZEB1-AS1 in lung epithelial cells reduces apoptosis and inflammatory injury partly through downregulation of p53-dependent pathways (Gu et al., 2020). Collectively, these findings indicate that ZEB1-AS1 acts as a protective regulator in diabetic complications, primarily by suppressing fibrosis, reducing apoptosis, and modulating metabolic stress signaling.

Interestingly, ZEB1-AS1 appears to exert opposite effects on EMT-related pathways in metabolic disease compared with cancer. In diabetic kidney models, ZEB1-AS1 upregulation increases expression of bone morphogenetic protein-7 (BMP7) and suppresses EMT markers, thereby inhibiting fibrosis and ECM deposition (Ghafouri-Fard et al., 2023). These observations suggest that the regulatory relationship between ZEB1-AS1 and ZEB1 may differ depending on tissue context and metabolic conditions. However, the precise molecular interplay between these factors in diabetes remains incompletely understood.

#### 4.9.4 Crosstalk and shared molecular pathways between CRC and T2D

Several molecular pathways regulated by ZEB1-AS1 are implicated in both CRC and metabolic diseases, suggesting potential mechanistic links between the two conditions. Notably, ZEB1-AS1 modulates signaling cascades such as PI3K/AKT, Wnt/β-catenin, and Hippo/YAP, all of which play central roles in tumor growth as well as metabolic regulation (Ghafouri-Fard et al., 2023; Wu G. et al., 2020; Jin and Chen, 2020). In CRC, activation of these pathways promotes cell proliferation, survival, and EMT, whereas in metabolic tissues they regulate processes such as fibrosis, insulin signaling, and cellular stress responses.

ZEB1-AS1-mediated regulation of EMT and fibrosis pathways also represents a potential point of convergence. EMT contributes to tumor metastasis in cancer but also drives fibrogenic remodeling in diabetic organs, including the kidney and heart. In CRC, ZEB1-AS1 enhances EMT by activating ZEB1 and repressing epithelial markers, whereas in diabetic tissues ZEB1-AS1 appears to counteract EMT-like processes by increasing BMP7 and suppressing fibrotic signaling (Ghafouri-Fard et al., 2023). These contrasting effects highlight the context-dependent regulatory role of ZEB1-AS1 in tissue remodeling pathways.

Another potential connection involves hypoxia-associated signaling. Experimental studies demonstrate that HIF-1α can induce ZEB1-AS1 transcription by binding hypoxia response elements within its promoter. In turn, ZEB1-AS1 stabilizes HIF-1α through ZEB1-dependent mechanisms, forming a feed-forward regulatory loop that enhances cellular adaptation to hypoxic stress (Lin et al., 2021). Hypoxia signaling is widely implicated in tumor progression and has also been linked to metabolic inflammation and diabetic tissue injury, suggesting that ZEB1-AS1 may participate in hypoxia-responsive gene networks relevant to both diseases.

Although these overlapping pathways suggest potential biological links, direct mechanistic studies connecting ZEB1-AS1 to both CRC and T2D within the same experimental framework remain limited. Current evidence therefore supports the idea that ZEB1-AS1 regulates shared signaling pathways involved in tumor progression and metabolic fibrosis, but its precise role in mediating disease crosstalk between CRC and diabetes requires further investigation.

#### 4.9.5 Crosstalk and immune/inflammatory links

Chronic inflammation represents a central pathogenic mechanism linking T2Ds and CRC, and emerging evidence suggests that ZEB1-AS1 may influence inflammatory signaling pathways relevant to both diseases. Several of the pathways modulated by ZEB1-AS1, including HIF-1α, PI3K/AKT, and YAP signaling, are known regulators of inflammatory responses and immune cell behavior within tumor and metabolic microenvironments.

Hypoxia-dependent signaling provides one potential link. Activation of HIF-1α not only promotes tumor angiogenesis and metastasis but also regulates inflammatory gene expression and immune cell recruitment. The reported HIF-1α/ZEB1-AS1 regulatory loop suggests that ZEB1-AS1 may contribute to hypoxia-driven inflammatory signaling within the tumor microenvironment (Lin et al., 2021). Similar hypoxia-associated inflammatory pathways are activated in diabetic tissues exposed to metabolic stress, indicating a possible shared regulatory context.

ZEB1-AS1 may also influence inflammatory cytokine signaling indirectly through regulation of EMT and fibrosis pathways. In cancer, EMT activation is associated with increased production of inflammatory mediators and recruitment of tumor-associated immune cells. Conversely, in diabetic organs, EMT-like transitions contribute to fibrotic inflammation and tissue remodeling. By modulating EMT regulators such as ZEB1, BMP7, and SIRT1, ZEB1-AS1 could potentially influence inflammatory signaling in both settings.

Furthermore, pathways controlled by ZEB1-AS1 intersect with transcriptional regulators of immune responses. For instance, SIRT1 signaling, which is enhanced by ZEB1-AS1 in DCM models, is known to suppress NF-κB–dependent inflammatory transcription and reduce cytokine production. Through this mechanism, ZEB1-AS1 may contribute to the regulation of inflammatory signaling in metabolic tissues (Wu et al., 2023). Although similar immune-regulatory effects have not yet been directly demonstrated in CRC, SIRT1 and NF-κB signaling are key modulators of the colorectal tumor microenvironment.

Overall, current evidence indicates that ZEB1-AS1 participates in regulatory networks involving hypoxia signaling, EMT-associated inflammation, and metabolic stress responses, which are relevant to both CRC and diabetic complications. However, most data derive from separate disease models, and direct experimental evidence linking ZEB1-AS1-mediated immune regulation to the CRC–T2D axis remains limited. Further studies investigating its role in cytokine signaling, immune cell infiltration, and inflammatory pathway activation may clarify whether ZEB1-AS1 represents a functional molecular bridge between metabolic disease and colorectal tumorigenesis.

### 4.10 SOX2OT

#### 4.10.1 Biological role

The long non-coding RNA SOX2 overlapping transcript (SOX2OT) is a multi-exonic transcript located on chromosome 3q26.3 that physically overlaps the pluripotency gene SOX2 and is transcribed in the same orientation. The SOX2OT genomic locus spans more than 700 kb, is evolutionarily conserved, and is highly expressed in embryonic stem cells, where its transcription is frequently co-regulated with SOX2 (Wang et al., 2022). In healthy adult tissues, SOX2OT expression is relatively low and largely restricted to the central nervous system, particularly the brain. However, aberrant upregulation of SOX2OT has been reported in multiple malignancies, including lung, esophageal, breast, and colorectal cancers, where increased expression is often associated with tumor progression and poor clinical outcomes (Shahryari et al., 2015; Liu et al., 2016).

Functionally, SOX2OT acts as a regulatory lncRNA involved in pluripotency maintenance, transcriptional regulation, and cellular stress responses. A substantial portion of its regulatory activity is mediated through ceRNA networks, whereby SOX2OT sequesters tumor-suppressive miRNAs and modulates the expression of downstream genes involved in proliferation and differentiation. In addition to miRNA sponging, SOX2OT participates in epigenetic and post-transcriptional regulation, including modulation of RNA methylation and protein stability.

Recent studies have also identified SOX2OT dysregulation in metabolic and diabetic conditions, including DN, DR, and gestational diabetes mellitus (GDM) (Ran et al., 2021; Chen et al., 2021). These findings suggest that SOX2OT may participate in both oncogenic signaling and metabolic stress responses, highlighting its potential role as a regulatory node connecting cancer biology and metabolic disease.

#### 4.10.2 Mechanisms in colorectal cancer

SOX2OT has been consistently reported to be upregulated in CRC tissues and cell lines, and elevated expression correlates with advanced tumor stage, lymph node metastasis, and unfavorable prognosis (Liu et al., 2016; Feng et al., 2021). Functional studies demonstrate that silencing SOX2OT significantly suppresses CRC cell proliferation, migration, and invasion, while inducing G0/G1 cell-cycle arrest.

Mechanistically, SOX2OT primarily promotes CRC progression through ceRNA activity. The transcript contains multiple binding sites for tumor-suppressive miRNAs and acts as a molecular sponge, thereby relieving repression of oncogenic targets. For example, SOX2OT has been shown to sequester miR-194-5p, leading to upregulation of SOX5, a transcription factor associated with increased tumor aggressiveness (Feng et al., 2021). Knockdown of SOX2OT results in elevated miR-194-5p levels, reduced SOX5 expression, and decreased proliferation and invasion of CRC cells.

Additional studies indicate that SOX2OT may also regulate miR-122-3p, further contributing to suppression of proliferation, migration, and EMT when SOX2OT expression is reduced (Feng et al., 2021; Dodangeh et al., 2023). At the cellular level, depletion of SOX2OT decreases expression of cell-cycle regulators such as Cyclin B1 and Cdc25C, while reversing EMT by downregulating N-cadherin and restoring E-cadherin expression (Liu et al., 2016). *In vivo* experiments using CRC xenograft models confirm that SOX2OT knockdown significantly inhibits tumor growth, further supporting its oncogenic function (Feng et al., 2021).

Beyond miRNA sponging, emerging evidence suggests that SOX2OT can regulate epigenetic and post-transcriptional processes that influence oncogenic signaling. For instance, SOX2OT has been shown to recruit the m^6^A RNA demethylase ALKBH5 to SOX2 mRNA, leading to reduced methylation, increased transcript stability, and enhanced SOX2 expression (Liu B. et al., 2020). Elevated SOX2 can subsequently activate Wnt/β-catenin signaling, a pathway central to colorectal tumorigenesis. However, this regulatory mechanism has been demonstrated primarily in glioblastoma, and its direct relevance to CRC remains suggestive rather than fully established.

#### 4.10.3 Mechanisms in type 2 diabetes and metabolic complications

In contrast to its oncogenic role in CRC, SOX2OT appears to exert context-dependent and often protective functions in diabetic complications. In DN, SOX2OT expression is significantly downregulated in renal tissues from streptozotocin-induced diabetic mice and in kidney cells exposed to high-glucose conditions (Chen et al., 2021).

Experimental restoration of SOX2OT expression in mesangial cells and podocytes suppresses high-glucose–induced cell proliferation, ECM accumulation, fibrosis, and apoptosis, while promoting autophagy (Chen et al., 2021; Zhang et al., 2019). Mechanistically, SOX2OT inhibits the Akt/mTOR signaling pathway, a central regulator of autophagy, thereby enhancing autophagic flux and alleviating renal injury.

SOX2OT also participates in a ceRNA regulatory axis in diabetic kidneys. Specifically, SOX2OT sponges miR-9, leading to increased expression of SIRT1, a key metabolic regulator involved in stress resistance and mitochondrial homeostasis. Activation of the SOX2OT–miR-9–SIRT1 pathway enhances autophagy and protects podocytes from hyperglycemia-induced damage (Zhang et al., 2019). These findings collectively support a renoprotective role for SOX2OT in DN.

However, SOX2OT exhibits a different pattern in DR. In retinal tissues of diabetic mice and in retinal ganglion cells exposed to high glucose, SOX2OT expression is reduced (Li CP. et al., 2017). Interestingly, further experimental silencing of SOX2OT decreases apoptosis and enhances antioxidant defenses through activation of the NRF2/HO-1 signaling pathway, thereby protecting retinal cells from oxidative stress–induced injury. This observation suggests that, in retinal tissues, reduced SOX2OT expression may facilitate adaptive antioxidant responses.

SOX2OT has also been associated with GDM. Elevated circulating levels of SOX2OT during early pregnancy have been detected in women who subsequently develop GDM, and higher expression correlates with an increased risk of adverse pregnancy outcomes, including greater fetal birth weight (Ran et al., 2021). These findings suggest that SOX2OT may contribute to metabolic dysregulation during pregnancy or potentially serve as an early biomarker for GDM risk.

#### 4.10.4 Crosstalk and shared molecular pathways between CRC and T2D

Despite the contrasting roles of SOX2OT in cancer and metabolic disease, several shared signaling pathways regulated by this lncRNA are relevant to both CRC and T2Ds.

One major point of convergence is the Akt/mTOR–autophagy axis. In DN, SOX2OT inhibits Akt/mTOR signaling to activate autophagy and protect renal cells from hyperglycemic injury (Chen et al., 2021). Autophagy also plays an important role in cancer biology, where it can regulate tumor cell survival, metabolic adaptation, and therapeutic resistance (Manzoor et al., 2022). Studies in other malignancies, such as glioblastoma, indicate that SOX2OT-mediated upregulation of SIRT1 can enhance autophagy and contribute to drug resistance (Liu B. et al., 2020). Although this mechanism has not been fully characterized in CRC, it suggests a potential link between SOX2OT, metabolic stress responses, and tumor survival pathways.

Another potential intersection involves Wnt/β-catenin signaling, a central driver of colorectal tumorigenesis. SOX2OT may indirectly influence this pathway through regulation of SOX2 expression, as demonstrated in glioblastoma models where SOX2OT recruits ALKBH5 to demethylate SOX2 transcripts (Liu B. et al., 2020). Increased SOX2 expression subsequently activates Wnt5a/β-catenin signaling, promoting tumor progression. While direct evidence in CRC remains limited, this mechanism highlights a plausible regulatory link between SOX2OT and Wnt signaling.

Oxidative stress regulation also represents an overlapping biological process. In DR models, SOX2OT modulates NRF2-dependent antioxidant signaling, influencing cellular responses to oxidative injury (Li CP. et al., 2017). Redox signaling similarly contributes to cancer progression by regulating cell survival, metabolism, and genomic stability, suggesting that SOX2OT may participate in broader stress-response pathways relevant to both diseases.

#### 4.10.5 Crosstalk and immune/inflammatory links

Inflammation and metabolic stress are key drivers of both T2Ds and CRC, and several SOX2OT-regulated pathways intersect with inflammatory signaling networks. In DN, the SIRT1 pathway, which is activated through the SOX2OT–miR-9 regulatory axis, is known to suppress NF-κB–mediated inflammatory transcription and reduce production of pro-inflammatory cytokines. Through this mechanism, SOX2OT may contribute indirectly to the attenuation of inflammatory responses in diabetic tissues.

Oxidative stress signaling also interacts closely with inflammatory pathways. Activation of the NRF2/HO-1 antioxidant system, observed following SOX2OT silencing in retinal cells, can inhibit inflammatory cytokine production and protect tissues from oxidative injury (Li CP. et al., 2017). NRF2 signaling has similarly been implicated in the modulation of inflammatory responses within the colorectal tumor microenvironment, suggesting a potential shared regulatory context.

In addition, pathways such as PI3K/AKT and Wnt/β-catenin, which may be influenced by SOX2OT through indirect regulatory mechanisms, are known to interact with inflammatory signaling networks including NF-κB and cytokine pathways. Although direct evidence linking SOX2OT to immune cell recruitment or cytokine regulation in CRC remains limited, its influence on these upstream signaling cascades suggests that it could contribute to inflammation-associated tumor progression and metabolic tissue injury.

Overall, current evidence indicates that SOX2OT participates in regulatory networks involving autophagy, oxidative stress responses, metabolic signaling, and inflammatory pathways that are relevant to both CRC and diabetic complications. However, most available data derive from separate disease models, and direct mechanistic studies connecting SOX2OT to the CRC–T2D axis remain limited. Further research is required to determine whether SOX2OT plays a functional role in mediating the molecular interplay between metabolic disease and colorectal tumorigenesis.

### 4.11 GAS5

#### 4.11.1 Biological role

Growth arrest-specific transcript 5 (GAS5) is a well-characterized long non-coding RNA located on chromosome 1q25, a genomic region frequently altered in several malignancies and involved in the regulation of cell proliferation, apoptosis, and cellular stress responses (Nguyen et al., 2025; Shi Y. et al., 2019). GAS5 is transcribed as a non-coding RNA that hosts multiple intronic snoRNAs. Due to the presence of premature stop codons, however, GAS5 transcripts are not translated into proteins (Shi Y. et al., 2019). Instead, GAS5 functions as a regulatory RNA through several mechanisms, including acting as a molecular decoy, scaffold, and ceRNA that sponges multiple miRNAs (Nguyen et al., 2025; Shi Y. et al., 2019).

In many cancers, including CRC, GAS5 is generally downregulated and functions as a tumor-suppressive lncRNA. Restoration of GAS5 expression has been shown to inhibit tumor cell proliferation, migration, and metastasis (Nguyen et al., 2025; Luo et al., 2020). Beyond oncology, emerging evidence indicates that GAS5 also plays an important role in metabolic regulation. Reduced circulating and tissue levels of GAS5 have been reported in patients with T2D, and experimental restoration of GAS5 improves insulin secretion and insulin sensitivity (Shi Y. et al., 2019; Luo et al., 2020).

Mechanistically, GAS5 regulates several cellular signaling networks that are relevant to both tumorigenesis and metabolic homeostasis, including PI3K/AKT signaling, Hippo/YAP signaling, and epigenetic regulatory pathways. GAS5 has also been linked to ubiquitin-mediated protein degradation, further highlighting its role as a multifunctional regulatory hub influencing cell growth, apoptosis, and metabolic signaling.

#### 4.11.2 Mechanisms in colorectal cancer

In CRC, GAS5 expression is typically significantly reduced in tumor tissues and cell lines, and decreased GAS5 levels are associated with increased tumor proliferation, migration, and xenograft growth (Shi Y. et al., 2019; Luo et al., 2020). Functional studies indicate that GAS5 acts as a tumor suppressor by modulating oncogenic miRNAs and signaling pathways involved in CRC progression.

One of the best-characterized mechanisms involves the GAS5/miR-21/LIFR regulatory axis. GAS5 acts as a molecular sponge for the oncogenic miR-21, thereby relieving repression of leukemia inhibitory factor receptor (LIFR) and promoting apoptosis. Through inhibition of miR-21, GAS5 also increases expression of PTEN, resulting in suppression of the PI3K/AKT/mTOR signaling pathway, a central driver of colorectal tumor growth (Shi Y. et al., 2019; Luo et al., 2020). Loss of GAS5 therefore contributes to hyperactivation of AKT signaling and enhanced tumor proliferation.

GAS5 also regulates the Hippo/YAP signaling pathway, which is critical for controlling cell proliferation and organ size. GAS5 has been shown to bind directly to the WW domain of Yes-associated protein (YAP), facilitating its phosphorylation and cytoplasmic retention. Phosphorylated YAP subsequently undergoes ubiquitin-mediated degradation, preventing its nuclear translocation and transcriptional activation of genes associated with cell growth and survival. This GAS5-dependent regulation of YAP significantly suppresses CRC cell proliferation and tumor progression (Ni et al., 2019).

In addition to these signaling pathways, GAS5 participates in cell-cycle and transcriptional regulation. Following DNA damage, GAS5 promotes expression of its intronic snoRNAs, which activate p53 signaling and induce cell-cycle arrest. GAS5 has also been shown to act as a scaffold for the transcription factor E2F1, enhancing its binding to the CDKN1B promoter and increasing expression of the cyclin-dependent kinase inhibitor p27^Kip1, thereby further suppressing tumor proliferation (Nguyen et al., 2025).

Genetic variation within the GAS5 locus may also influence CRC susceptibility. For example, the rs55829688 T>C polymorphism in the GAS5 promoter has been associated with altered transcriptional activity and changes in CRC risk (Wang et al., 2019b). Although most evidence supports a tumor-suppressive role for GAS5, a small number of studies have reported elevated GAS5 expression in specific CRC cohorts, suggesting that its biological effects may be context dependent and warrant further investigation.

#### 4.11.3 Mechanisms in type 2 diabetes

In metabolic disorders, GAS5 is likewise downregulated in patients with T2D and plays a significant role in regulating insulin secretion and insulin sensitivity. Circulating GAS5 levels are significantly lower in individuals with T2D compared with healthy controls and show an inverse correlation with fasting glucose and HbA1c levels.

In pancreatic β-cells, overexpression of GAS5 enhances glucose-stimulated insulin secretion and increases intracellular insulin content, whereas GAS5 knockdown impairs these processes. Mechanistically, GAS5 functions as a ceRNA that sequesters several miRNAs, including miR-29a-3p, miR-96-3p, and miR-208a-3p, which normally suppress components of the insulin signaling pathway. By inhibiting these miRNAs, GAS5 increases expression of key signaling molecules such as the insulin receptor (IR), insulin receptor substrate (IRS), and PI3K regulatory subunits, thereby strengthening insulin signaling and improving glucose uptake (Luo et al., 2020).

GAS5 also regulates insulin sensitivity in adipose tissue through transcriptional control of the insulin receptor gene. GAS5 has been shown to bind the promoter region of the IR gene, enhancing its transcription, whereas depletion of GAS5 disrupts insulin signaling and reduces glucose uptake in adipocytes. Pharmacological stabilization of GAS5 using the small molecule NP-C86 prevents its degradation and restores glucose uptake in diabetic adipocytes, providing evidence that GAS5 directly regulates insulin signaling genes in metabolic tissues (Shi Y. et al., 2019).

Collectively, these findings indicate that GAS5 functions as a key metabolic regulator, and its downregulation contributes to insulin resistance and β-cell dysfunction, whereas restoration of GAS5 expression improves insulin signaling and glucose homeostasis (Shi Y. et al., 2019; Luo et al., 2020).

#### 4.11.4 Crosstalk and shared molecular pathways between CRC and T2D

Several signaling pathways regulated by GAS5 are involved in both CRC and metabolic disease, suggesting potential mechanistic links between these conditions. One of the most prominent shared pathways is PI3K/AKT signaling. In CRC, GAS5 suppresses PI3K/AKT activation by sponging miR-21 and restoring PTEN expression, thereby inhibiting tumor cell proliferation (Nguyen et al., 2025; Luo et al., 2020). In contrast, in metabolic tissues GAS5 enhances insulin-dependent PI3K/AKT signaling by increasing expression of IR and IRS, promoting glucose uptake and metabolic homeostasis (Luo et al., 2020). Thus, GAS5 deficiency may simultaneously facilitate oncogenic AKT signaling in cancer while impairing insulin-mediated AKT activation in metabolic tissues.

GAS5 also regulates cellular homeostasis through the Hippo/YAP signaling pathway. In CRC, GAS5 promotes phosphorylation and ubiquitin-mediated degradation of YAP, preventing its nuclear accumulation and transcriptional activation of growth-promoting genes (Ni et al., 2019). Dysregulation of Hippo/YAP signaling has also been implicated in metabolic disorders, including insulin resistance and hepatic metabolic dysfunction, suggesting that GAS5-mediated YAP regulation may influence both proliferative and metabolic pathways.

In addition to these pathways, GAS5 exerts epigenetic regulatory effects through interactions with chromatin-modifying proteins and miRNA networks. GAS5 has been reported to act as a ceRNA for oncogenic miRNAs such as miR-182-5p, restoring expression of tumor suppressors including FOXO1 and SMAD4 (Nguyen et al., 2025). GAS5 can also interact with the histone methyltransferase EZH2, limiting H3K27me3-mediated silencing of tumor-suppressor genes such as CDKN1B. Similar ceRNA-mediated regulatory mechanisms may operate in metabolic tissues, where GAS5 preserves expression of insulin signaling components by sequestering inhibitory miRNAs.

#### 4.11.5 Crosstalk and immune/inflammatory links

Chronic low-grade inflammation is a hallmark of both T2Ds and CRC, and several signaling pathways regulated by GAS5 intersect with inflammatory networks. In CRC, suppression of PI3K/AKT signaling by GAS5 can indirectly limit activation of downstream inflammatory mediators such as NF-κB, which plays a central role in tumor-associated inflammation and cytokine production. Loss of GAS5 may therefore contribute to enhanced inflammatory signaling within the colorectal tumor microenvironment.

In metabolic tissues, GAS5-mediated enhancement of insulin signaling and metabolic homeostasis may also reduce inflammatory stress associated with insulin resistance. Improved insulin signaling has been linked to reduced activation of NF-κB and pro-inflammatory cytokine pathways, suggesting that GAS5 restoration could indirectly attenuate inflammatory responses in T2D.

Furthermore, several transcription factors regulated by GAS5, including FOXO1 and SIRT1-associated metabolic regulators, are known modulators of inflammatory gene expression and oxidative stress responses. Through these networks, GAS5 may influence inflammatory signaling pathways that contribute to both metabolic tissue injury and tumor progression. However, most current evidence derives from separate disease models, and direct experimental studies linking GAS5-mediated immune regulation to the CRC–T2D axis remain limited.

Overall, GAS5 represents a multifunctional lncRNA that regulates PI3K/AKT signaling, Hippo/YAP activity, miRNA networks, epigenetic modifications, and ubiquitin-mediated protein degradation, thereby coordinating cellular proliferation, apoptosis, metabolic homeostasis, and inflammatory signaling. These convergent regulatory functions highlight the potential of GAS5 as a shared biomarker and therapeutic target for both CRC and T2D, although further research is required to clarify its context-dependent effects and to develop tissue-specific therapeutic strategies.

### 4.12 MIR31HG

#### 4.12.1 Biological role

MIR31HG (MIR31 host gene), also known as LncHIFCAR or LOC554202, is a ∼2.1-kb long non-coding RNA located on chromosome 9p21.3 that hosts miR-31 within its second intron. MIR31HG is broadly expressed and has been implicated in diverse physiological and pathological processes, including cell proliferation, EMT, metastasis, cellular senescence, and apoptosis (Ruan et al., 2023). Dysregulated MIR31HG expression has been reported in several diseases, including multiple cancers, psoriasis, and IgA nephropathy, highlighting its role as a multifunctional regulatory RNA (Ruan et al., 2023).

In cancer biology, MIR31HG frequently acts as a pro-tumorigenic lncRNA, and increased expression has been associated with aggressive tumor phenotypes and poor clinical outcomes in several malignancies, including CRC (Wang et al., 2022; Guo et al., 2021). Beyond oncology, emerging studies indicate that MIR31HG also participates in metabolic and tissue-repair processes, including adipocyte differentiation, wound healing, and diabetic neuropathy (Ruan et al., 2023; Huang et al., 2017; Groeneweg et al., 2020; Shen et al., 2022). Although these observations arise from distinct experimental systems, they suggest that MIR31HG may influence both tumorigenesis and metabolic disorders through overlapping regulatory pathways.

#### 4.12.2 Mechanisms in colorectal cancer

In CRC, MIR31HG has been shown to function primarily as an oncogenic lncRNA that promotes tumor proliferation, invasion, and metabolic adaptation. Several studies indicate that MIR31HG contributes to tumor progression through activation of key oncogenic signaling pathways.

One major mechanism involves the Wnt/β-catenin signaling pathway. MIR31HG has been reported to enhance β-catenin nuclear translocation and transcriptional activity, thereby promoting the expression of downstream genes that drive proliferation and survival (Wang et al., 2022; Ruan et al., 2023). MIR31HG also activates the PI3K/AKT/mTOR pathway, which further supports tumor growth, invasion, and resistance to cellular stress (Wang et al., 2022; Ruan et al., 2023). These signaling networks are central regulators of CRC progression and are recurrently influenced by oncogenic lncRNAs.

MIR31HG additionally functions as a ceRNA. For example, it has been shown to sponge miR-361-3p, thereby relieving repression of the transcription factor Yin Yang 1 (YY1). YY1 in turn enhances MIR31HG transcription, creating a positive feedback loop that amplifies oncogenic signaling and promotes tumor proliferation, angiogenesis, and metabolic activity (Guo et al., 2021).

Another important regulatory feature of MIR31HG involves hypoxia signaling. MIR31HG interacts with hypoxia-inducible factor-1α (HIF-1α), particularly under hypoxic tumor conditions, and enhances transcriptional programs associated with metastasis and therapy resistance. MIR31HG also regulates cellular senescence in a context-dependent manner. Nuclear MIR31HG can modulate the p16^INK4A/pRB pathway, whereas cytoplasmic MIR31HG influences the senescence-associated secretory phenotype (SASP) by promoting YBX1-mediated translation of IL1A, thereby increasing inflammatory signaling within the tumor microenvironment (Manzoor et al., 2022). Collectively, these findings position MIR31HG as a multifunctional regulator of CRC progression.

#### 4.12.3 Mechanisms in type 2 diabetes

Evidence linking MIR31HG to T2D primarily derives from studies examining metabolic regulation and diabetes-related complications. Although direct mechanistic studies in classical T2D models remain limited, several investigations suggest that MIR31HG influences processes relevant to metabolic homeostasis.

In human adipose-derived stem cells, MIR31HG promotes adipocyte differentiation, whereas MIR31HG knockdown significantly suppresses adipogenesis (Huang et al., 2017). This effect appears to involve epigenetic regulation of adipogenic gene expression. Specifically, depletion of MIR31HG reduces activating histone modifications—including H3K4 trimethylation (H3K4me3) and histone H3 acetylation—at the promoter of the adipogenic gene FABP4, leading to decreased transcription and impaired adipocyte maturation (Huang et al., 2017). Through this mechanism, MIR31HG may influence adipose tissue development and systemic energy balance, processes closely linked to obesity and insulin resistance, two major risk factors for T2D.

MIR31HG has also been implicated in diabetic wound healing. Exosomal MIR31HG delivered to diabetic skin tissues has been reported to regulate HIF-1α signaling, enhancing fibroblast proliferation and promoting tissue repair in diabetic ulcers (Shen et al., 2022). In addition, clinical observations indicate that circulating MIR31HG levels are reduced in patients with diabetic neuropathy compared with healthy individuals, suggesting that dysregulation of this lncRNA may contribute to diabetes-associated complications (Groeneweg et al., 2020). While these findings support a role for MIR31HG in metabolic disease contexts, further studies are needed to clarify its direct involvement in systemic glucose metabolism and T2D pathogenesis.

#### 4.12.4 Crosstalk and shared molecular pathways between CRC and T2D

Several pathways regulated by MIR31HG are implicated in both tumorigenesis and metabolic disease, suggesting potential mechanistic intersections between CRC and T2D.A major shared mechanism involves metabolic reprogramming and glycolysis. In CRC, MIR31HG promotes the Warburg effect by enhancing expression of glycolytic enzymes and supporting rapid tumor growth. Altered glucose metabolism and dysregulated glycolytic signaling are also central features of diabetes and its complications. Although direct evidence connecting MIR31HG-mediated metabolic regulation in CRC and T2D is currently limited, these observations suggest that MIR31HG may influence cellular energy metabolism in both pathological contexts.

Another convergence point involves hypoxia and angiogenesis signaling. MIR31HG regulates HIF-1α activity and VEGFA expression, promoting angiogenesis and vascular remodeling in tumor tissues. Hypoxia signaling pathways also contribute to diabetic vascular complications and impaired wound healing, suggesting that MIR31HG-dependent regulation of HIF-1α may influence vascular responses in both cancer and diabetic tissues (Shen et al., 2022).

MIR31HG further participates in epigenetic regulation by modulating chromatin structure and histone modifications. In adipogenic cells, MIR31HG controls histone activation marks at metabolic gene promoters (Huang et al., 2017). In cancer models, MIR31HG has been shown to interact with chromatin-modifying complexes such as WDR5, MLL3, and P300, promoting deposition of H3K4me1 and H3K27ac at oncogenic promoters and enhancing transcriptional activation (Wang et al., 2019b). Although most of these epigenetic mechanisms have been characterized outside CRC, they illustrate the capacity of MIR31HG to influence gene expression programs relevant to both metabolic and oncogenic processes.

MIR31HG may also regulate protein stability through ubiquitin-mediated pathways. For example, in gastric cancer models, MIR31HG interacts with the E3 ubiquitin ligase c-CBL, reducing β-catenin ubiquitination and stabilizing Wnt/β-catenin signaling. While this mechanism has not yet been directly demonstrated in CRC or metabolic tissues, it highlights a potential regulatory mechanism by which MIR31HG could modulate signaling proteins involved in proliferation and metabolism.

#### 4.12.5 Crosstalk and immune/inflammatory links

Chronic inflammation is a shared feature of both colorectal tumorigenesis and metabolic disorders, and MIR31HG participates in several inflammatory signaling networks. One important mechanism involves regulation of the senescence-associated secretory phenotype (SASP). MIR31HG enhances YBX1-mediated translation of IL1A, which stimulates production of pro-inflammatory cytokines and reinforces tumor-associated inflammatory signaling (Manzoor et al., 2022). Increased IL-1–dependent signaling can activate downstream inflammatory pathways, including NF-κB, thereby promoting tumor progression and shaping the tumor microenvironment.

Hypoxia signaling mediated by HIF-1α also intersects with inflammatory pathways. MIR31HG-dependent activation of HIF-1α can enhance angiogenesis and cytokine production in tumor tissues, while HIF-1α signaling is likewise involved in inflammatory and hypoxic responses in diabetic tissues, particularly in chronic wounds and vascular complications (Shen et al., 2022). These overlapping regulatory networks suggest that MIR31HG may influence both hypoxia-driven inflammation and metabolic stress responses.

Overall, MIR31HG integrates multiple regulatory layers—including Wnt/β-catenin, PI3K/AKT/mTOR, HIF-1α signaling, epigenetic chromatin remodeling, and inflammatory cytokine pathways—that are relevant to both CRC progression and metabolic disease. However, most available evidence derives from separate experimental contexts, and direct mechanistic studies linking MIR31HG to a unified CRC–T2D axis remain limited. Further investigation will therefore be necessary to clarify whether MIR31HG could function as a shared biomarker or therapeutic target in patients affected by both CRC and metabolic disorders.

**FIGURE 2:**
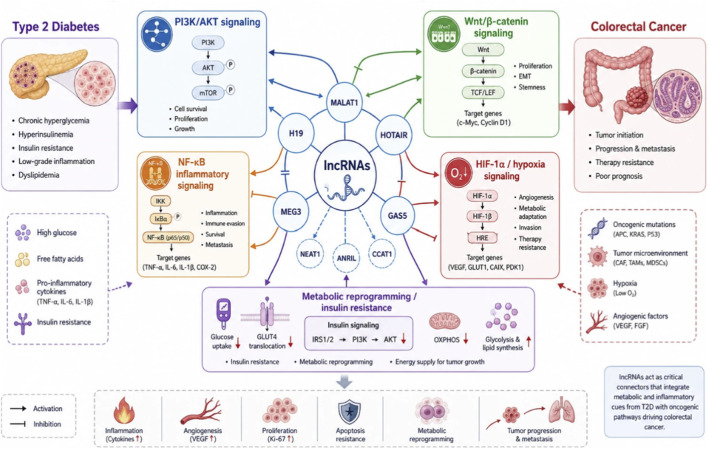
Integrative network of lncRNAs linking T2D and colorectal cancer. Schematic representation of the major long non-coding RNAs (lncRNAs) involved in the molecular crosstalk between type 2 diabetes (T2D) and colorectal cancer (CRC). Key lncRNAs, including MALAT1, H19, HOTAIR, MEG3, and GAS5, interact with multiple signaling pathways associated with insulin resistance, inflammation, metabolic reprogramming, angiogenesis, proliferation, and tumor progression. The figure highlights shared pathways such as PI3K/AKT, Wnt/β-catenin, NF-κB, and HIF-1α signaling, emphasizing the integrative role of lncRNAs in diabetes-associated colorectal carcinogenesis.

As illustrated in Figure 2, the major lncRNAs implicated in both T2D and CRC interact with multiple shared signaling pathways, including PI3K/AKT, Wnt/β-catenin, NF-κB, and HIF-1α, thereby contributing to metabolic dysregulation, inflammation, and colorectal tumor progression.

## 5 Integrative model of diabetes-driven carcinogenesis via lncRNAs

A growing body of evidence indicates that lncRNAs function as critical regulatory molecules connecting metabolic disorders with cancer development. In the context of DM and CRC, multiple lncRNAs modulate overlapping molecular pathways that regulate cellular metabolism, inflammation, survival, and tumor progression. These pathways form a complex regulatory network in which metabolic stress associated with diabetes influences lncRNA expression, thereby activating oncogenic signaling cascades involved in colorectal tumorigenesis. The lncRNAs discussed in this review, including MALAT1, H19, ANRIL, KCNQ1OT1, MIAT, UCA1, HNF1A-AS1, ZEB1-AS1, SOX2OT, SNHG4, GAS5, and MIR31HG, collectively regulate several key pathways that play fundamental roles in both metabolic dysregulation and cancer biology.

### 5.1 PI3K/AKT/mTOR signaling

The phosphatidylinositol 3-kinase (PI3K)/AKT/mTOR pathway is one of the most important signaling cascades linking metabolic regulation to cancer development (Glaviano et al., 2023; Wang et al., 2021). In diabetic conditions, hyperinsulinemia and enhanced insulin-like growth factor-1 (IGF-1) signaling stimulate PI3K activation, leading to phosphorylation of AKT and subsequent activation of the mammalian target of rapamycin (mTOR) (Zhao et al., 2023; Wang et al., 2021). This pathway regulates cell growth, proliferation, protein synthesis, and metabolic processes.

Several lncRNAs implicated in both diabetes and CRC modulate the PI3K/AKT/mTOR pathway. For example, MALAT1 has been shown to activate PI3K/AKT signaling, promoting CRC cell proliferation and survival (Xu et al., 2022). Similarly, H19 and UCA1 regulate downstream targets of this pathway through ceRNAs mechanisms, thereby enhancing tumor cell growth and metabolic adaptation (Yang et al., 2021; Yuan et al., 2022). KCNQ1OT1 and ANRIL also influence PI3K/AKT signaling through epigenetic regulation and interaction with miRNAs that target pathway components (Zhu et al., 2021; Lin et al., 2021). In diabetic tissues, dysregulation of these lncRNAs contributes to insulin resistance and inflammatory signaling, while in CRC they facilitate tumor progression and resistance to apoptosis (Zhang X. et al., 2025; Glaviano et al., 2023).

### 5.2 Wnt/β-catenin pathway

The Wnt/β-catenin pathway plays a central role in intestinal epithelial homeostasis and is a major driver of colorectal carcinogenesis (He and Gan, 2023). Activation of Wnt signaling leads to stabilization and nuclear accumulation of β-catenin, which interacts with transcription factors to promote the expression of genes involved in proliferation, stemness, and cell survival (He and Gan, 2023; Zhang and Wang, 2020).

Multiple lncRNAs associated with diabetes and CRC regulate the Wnt/β-catenin pathway. MALAT1 and H19 can enhance β-catenin signaling by sponging miRNAs that normally suppress Wnt pathway components (Xu et al., 2022; Chowdhury et al., 2023). ZEB1-AS1 and SOX2OT have also been reported to activate Wnt signaling, thereby promoting EMT and metastatic potential in CRC cells (Lv et al., 2018; Wang et al., 2019c). In the metabolic context of diabetes, alterations in Wnt signaling may influence cellular metabolism and tissue remodeling (Maschio et al., 2016), suggesting that lncRNA-mediated regulation of this pathway contributes to both metabolic dysfunction and tumorigenesis.

### 5.3 NF-κB inflammatory signaling

Chronic inflammation is a well-recognized link between metabolic diseases and cancer. NF-κB signaling pathway is a key mediator of inflammatory responses and regulates the expression of numerous cytokines, chemokines, and survival genes (Indira and Abhilash, 2013; Xia et al., 2014). In diabetes, persistent metabolic stress leads to sustained activation of NF-κB signaling, promoting the production of inflammatory mediators such as TNF-α and IL-6(129).

Several lncRNAs modulate NF-κB signaling in both diabetes and CRC. ANRIL and MALAT1 have been reported to enhance NF-κB activity, thereby amplifying inflammatory responses and promoting tumor cell proliferation and survival (Gupta et al., 2020; Ma and Hu, 2023). MIAT and SNHG4 also participate in inflammatory regulatory networks by interacting with miRNAs that control NF-κB pathway components (Rostom et al., 2025; Yu et al., 2023). Through these mechanisms, lncRNAs contribute to the establishment of a chronic inflammatory microenvironment that facilitates colorectal tumor initiation and progression in diabetic individuals.

### 5.4 Hypoxia and HIF-1α pathway

Hypoxia is a common feature of both diabetic tissues and rapidly growing tumors. Under low-oxygen conditions, HIF-1α becomes stabilized and activates the transcription of genes involved in angiogenesis, metabolic adaptation, and cell survival (Zeng et al., 2022; Mu et al., 2023). This pathway enables tumor cells to adapt to oxygen-deficient environments and supports aggressive tumor growth.

Several lncRNAs regulate the HIF-1α signaling pathway. MALAT1 and MIR31HG have been shown to enhance HIF-1α stability and activity, thereby promoting hypoxia-induced gene expression and tumor angiogenesis (Xu et al., 2022; Guo et al., 2021). UCA1 and SOX2OT can also influence hypoxia-responsive signaling networks, contributing to metabolic adaptation and tumor progression (Li et al., 2014; Wang et al., 2019c). In diabetic tissues, hypoxia and oxidative stress further amplify these regulatory mechanisms, highlighting the role of lncRNAs in coordinating hypoxia-associated responses in both metabolic disease and cancer (Dehghanzad et al., 2024; Yan, 2014).

### 5.5 VEGF-mediated angiogenesis

Angiogenesis, the formation of new blood vessels, is essential for tumor growth and metastasis. VEGF is a key regulator of angiogenesis and is frequently upregulated in both diabetic complications and malignant tumors (Xia et al., 2014; Thomas et al., 2017). Increased VEGF expression promotes vascular remodeling, endothelial cell proliferation, and increased vascular permeability.

Several lncRNAs influence VEGF signaling and angiogenic processes. MALAT1, UCA1, and MIR31HG have been reported to regulate VEGF expression either directly or through miRNA-mediated mechanisms (Huang et al., 2020; Yan et al., 2021; Guo et al., 2021). In diabetes, dysregulated angiogenesis contributes to complications such as DR and nephropathy (Groeneweg et al., 2020; Thomas et al., 2017), whereas in CRC it facilitates tumor vascularization and metastatic dissemination (Dehghanzad et al., 2024). By modulating VEGF-related pathways, lncRNAs serve as important regulators of angiogenic responses in both pathological contexts.

### 5.6 Metabolic reprogramming

Metabolic reprogramming is a hallmark of cancer and involves alterations in cellular energy metabolism that support rapid proliferation and survival (Ren et al., 2025; Wang K. et al., 2024). One of the most prominent metabolic changes in cancer cells is the shift toward aerobic glycolysis, commonly known as the Warburg effect (Fan et al., 2022; Ren et al., 2025). This metabolic adaptation allows cancer cells to generate energy and biosynthetic intermediates necessary for tumor growth.

LncRNAs play important roles in regulating metabolic pathways associated with glucose utilization, glycolysis, and mitochondrial function (Lin et al., 2020; Wang K. et al., 2024; Xu et al., 2021). For instance, UCA1 has been reported to promote glycolytic metabolism in CRC through activation of mTOR/STAT3 signaling and upregulation of glycolytic enzymes such as hexokinase 2 (Li et al., 2014). HNF1A-AS1 also regulates metabolic pathways by influencing transcriptional networks associated with glucose metabolism (Sepehri et al., 2022; Guo et al., 2020). In diabetic conditions characterized by chronic hyperglycemia, these lncRNA-mediated metabolic changes may further enhance the metabolic flexibility of CRC cells, thereby promoting tumor progression (Xu et al., 2021; Wang et al., 2019c).

### 5.7 Oxidative stress pathways

Oxidative stress represents another important mechanism linking diabetes with cancer development (Black, 2024). Chronic hyperglycemia increases the production of ROS, leading to oxidative damage and activation of stress-responsive signaling pathways (Yan, 2014). Excessive ROS can cause DNA damage, genomic instability, and alterations in cellular signaling that contribute to malignant transformation (Zhao et al., 2023).

Several lncRNAs are involved in the regulation of oxidative stress responses (Wang et al., 2019c). SOX2OT and SNHG4 have been associated with pathways controlling redox balance and cellular stress responses, while GAS5 has been reported to modulate signaling pathways that influence oxidative stress and apoptosis. Through interactions with antioxidant systems and stress-responsive transcription factors, these lncRNAs help determine cellular responses to oxidative damage. In the context of diabetes, increased oxidative stress may therefore trigger lncRNA-mediated regulatory mechanisms that facilitate colorectal carcinogenesis.

Collectively, these shared molecular pathways highlight the integrative role of lncRNAs in coordinating metabolic, inflammatory, and oncogenic signaling networks. By simultaneously influencing multiple signaling cascades, lncRNAs function as molecular bridges that connect metabolic dysfunction in diabetes with CRC development and progression.

## 6 Disease progression model linking diabetes to colorectal cancer

**FIGURE 3:**
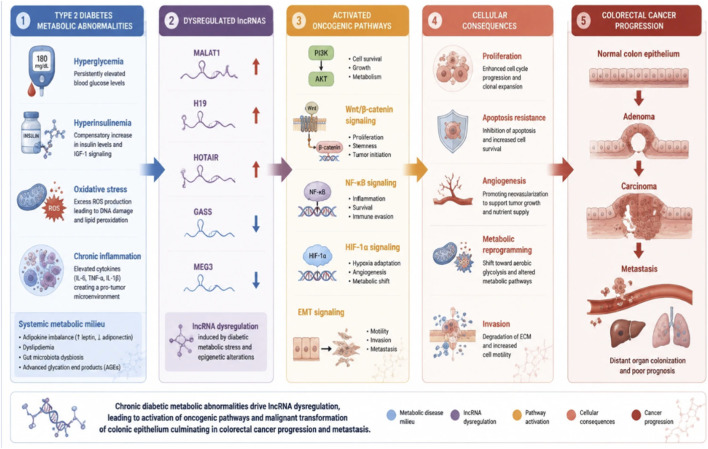
Proposed model of diabetes-driven colorectal carcinogenesis mediated by lncRNA dysregulation. Illustration of the sequential molecular events linking type 2 diabetes to colorectal cancer development. Chronic hyperglycemia, hyperinsulinemia, oxidative stress, and systemic inflammation promote dysregulation of specific lncRNAs, which subsequently modulate oncogenic signaling pathways and cellular processes including proliferation, apoptosis resistance, epithelial–mesenchymal transition, angiogenesis, and metabolic adaptation. These cumulative alterations contribute to colorectal tumor initiation, progression, and metastasis.

Accumulating evidence suggests that diabetes mellitus contributes to CRC development through a continuum of metabolic and molecular dysregulation. LncRNAs act as integrative regulatory hubs coupling metabolic stress with oncogenic signaling. The proposed model describes how diabetic metabolic abnormalities initiate and amplify tumorigenic cascades through a network of interacting lncRNAs (Figure 3).

### 6.1 Diabetic metabolic stress

Diabetes-related hyperglycemia, insulin resistance, hyperinsulinemia, and chronic inflammation activate multiple stress-signaling pathways. Persistent glucose elevation generates ROS and advanced glycation end products (AGEs), while aberrant insulin and IGF-1 signaling stimulate mitogenic activity (Chen et al., 2024; Gallagher and LeRoith, 2010). These events create a microenvironment enriched in inflammatory cytokines, growth factors, and hypoxic stress, conditions that promote transcriptional activation of oncogenic and stress-responsive lncRNAs.

### 6.2 LncRNA dysregulation

Diabetes-related hyperglycemia, insulin resistance, hyperinsulinemia, and chronic inflammation activate multiple stress-signaling pathways. Persistent glucose elevation generates ROS and advanced glycation end products (AGEs), while aberrant insulin and IGF-1 signaling stimulate mitogenic activity. Together, these events establish a microenvironment enriched in inflammatory cytokines, growth factors, and hypoxic stress—conditions that promote transcriptional activation of oncogenic and stress-responsive lncRNAs. Metabolic stress and inflammation further reshape the epigenetic and transcriptional landscapes, resulting in dysregulation of lncRNAs such as MALAT1, H19, ANRIL, KCNQ1OT1, MIAT, UCA1, MIR31HG, and others (Ghahramani et al., 2025; Li et al., 2025; Binjaw et al., 2023; Zhou et al., 2026). Several of these lncRNAs act as ceRNAs that sequester miRNAs targeting metabolic or proliferative genes, whereas others interact with chromatin-modifying complexes and transcription factors to reprogram gene expression networks linked to both diabetes and CRC (Chodurska and Kunej, 2025; Ma et al., 2023; Han and Chang, 2015). Importantly, their effects are not independent: multiple lncRNAs converge on overlapping regulatory circuits, leading to synergistic, antagonistic, or redundant behaviors. For example, H19, MIR31HG, and ZEB1-AS1 can cooperatively enhance Wnt/β-catenin signaling and promote EMT, thereby supporting tumor proliferation and metabolic reprogramming, while GAS5 may counterbalance oncogenic lncRNAs by attenuating PI3K/AKT signaling. In addition, functional redundancy has been suggested by overlapping control of gene sets through ceRNA activity and epigenetic silencing by ANRIL, SNHG4, and HNF1A-AS1, potentially converging at shared nodal regulators such as p53, EZH2, and CDKN2A/B. Although many of these interactions are inferred from studies conducted in distinct experimental systems, the overall emerging pattern supports the view that a diabetic–onco–lncRNA network behaves as an interconnected system rather than as a collection of isolated molecular players.

### 6.3 Activation of oncogenic pathways

Dysregulated lncRNAs influence several key signaling pathways implicated in both diabetes and CRC (Dehghanzad et al., 2024; Chen et al., 2019). Mechanistically supported evidence demonstrates that H19 directly activates Wnt/β-catenin signaling, ANRIL epigenetically represses tumor suppressor genes such as CDKN2A/B, and GAS5 modulates the PI3K/AKT pathway (Bhan et al., 2017). In addition to these well-characterized interactions, other lncRNAs have been associated with pathway alterations based on expression correlations, although direct causative evidence remains to be established. These affected pathways include PI3K/AKT/mTOR, Wnt/β-catenin, NF-κB–mediated inflammatory signaling, HIF-1α–mediated hypoxia responses, and VEGF-driven angiogenesis. Collectively, dysregulation of these signaling networks promotes cellular survival, proliferation, and resistance to apoptosis, thereby creating a permissive environment for tumor initiation and progression.

### 6.4 Tumor initiation

Chronic activation of these pathways causes DNA damage, genomic instability, and deregulated growth in colorectal epithelial cells. Epigenetic mechanisms, such as ANRIL-mediated repression of CDKN2A/B and H19-dependent β-catenin stabilization, represent experimentally supported causal events initiating tumorigenic transformation. Concurrently, redundant lncRNA activity in stress-response pathways ensures persistence of maladaptive gene expression even if one node is repressed, reinforcing the transition to a malignant phenotype.

### 6.5 CRC progression and metastasis

During tumor progression, synergistic lncRNA circuits (e.g., H19 + MIR31HG enhancing Wnt signaling; ZEB1-AS1 + SNHG4 promoting EMT) facilitate invasion, angiogenesis, and immune evasion. Antagonistic influences, such as GAS5-mediated suppression of PI3K/AKT, may be lost through downregulation, removing natural inhibitory checks. Redundant regulatory loops involving ANRIL and HNF1A-AS1 sustain transcriptional memory of oncogenic states.

## 7 Therapeutic and clinical implications

Growing evidence indicates that multiple lncRNAs regulate shared molecular pathways between CRC and T2D, highlighting important clinical implications for risk assessment, early detection, and therapeutic targeting. Diabetes is a well-recognized risk factor for CRC, and the identification of common regulatory lncRNAs provides mechanistic insight into this epidemiological association. LncRNAs such as MALAT1, H19, ANRIL, KCNQ1OT1, MIAT, UCA1, HNF1A-AS1, ZEB1-AS1, SOX2OT, SNHG4, GAS5, and MIR31HG influence key pathways including PI3K/AKT signaling, Wnt/β-catenin signaling, NF-κB-mediated inflammation, hypoxia signaling, metabolic reprogramming, and angiogenesis. These pathways contribute to both tumor development and metabolic dysregulation, suggesting that lncRNAs may function as molecular bridges linking metabolic disease with tumorigenesis.

From a diagnostic perspective, several of these lncRNAs have been detected in circulating blood, plasma, serum, or exosomal fractions, highlighting their potential as non-invasive biomarkers. Circulating lncRNAs can remain relatively stable in the bloodstream because they are frequently packaged within extracellular vesicles such as exosomes and microvesicles or associated with RNA-binding proteins and lipoprotein complexes, which protect them from RNase-mediated degradation. Previous studies have demonstrated that exosomal lncRNAs such as H19, MALAT1, and GAS5 can be reliably detected in plasma and may reflect disease-related molecular changes in both metabolic disorders and cancer. These findings support the possibility that circulating lncRNA signatures could improve risk stratification, early detection of CRC in diabetic populations, and monitoring of disease progression or therapeutic response.

Therapeutically, targeting dysregulated lncRNAs represents a promising strategy for modulating disease-associated signaling networks. Several RNA-based approaches are currently being explored, including antisense oligonucleotides (ASOs), small interfering RNAs (siRNAs), short hairpin RNAs, and CRISPR-based transcriptional modulation. These strategies may enable selective inhibition of oncogenic lncRNAs or restoration of protective lncRNAs that regulate metabolic homeostasis and tumor suppression. However, effective therapeutic implementation requires overcoming important challenges, including RNA stability, delivery efficiency, and tissue specificity.

Recent advances in RNA delivery technologies have begun to address these challenges. For example, lipid nanoparticles, polymer-based nanocarriers, and ligand-modified delivery systems have shown potential for improving nucleic-acid delivery to specific tissues, including the gastrointestinal tract. Colon-targeted delivery strategies, such as pH-responsive nanoparticles, microbiota-responsive carriers, and antibody- or ligand-conjugated nanoparticles that recognize intestinal epithelial receptors, have demonstrated promising results in experimental models of intestinal disease. Although most lncRNA-targeted therapies remain at the preclinical stage, these approaches provide a potential framework for achieving selective delivery to the colonic epithelium while minimizing off-target effects in other organs.

An additional challenge arises from the context-dependent functions of certain lncRNAs. Some molecules may act as oncogenes in CRC while exerting protective or compensatory effects in metabolic tissues. For example, lncRNAs that promote tumor growth through activation of proliferative signaling pathways may simultaneously participate in metabolic adaptation or stress responses in pancreatic, hepatic, or adipose tissues. Consequently, future therapeutic strategies will require careful consideration of tissue-specific expression patterns, delivery strategies, and pathway selectivity to avoid unintended systemic effects.

Finally, integrating lncRNA expression signatures with clinical and metabolic parameters may facilitate the development of precision medicine approaches for patients affected by both CRC and diabetes. Multi-parameter models incorporating lncRNA profiles, inflammatory markers, and metabolic indicators could improve individualized screening strategies, prognostic prediction, and therapeutic decision-making. Overall, continued investigation of lncRNA-mediated regulatory networks at the interface of metabolic disease and colorectal tumorigenesis may provide new opportunities for biomarker development, mechanistic insight, and integrated therapeutic interventions.

## 8 Future directions

Despite increasing evidence supporting the involvement of lncRNAs in the shared molecular mechanisms linking CRC and diabetes, several critical knowledge gaps and methodological limitations remain. Addressing these challenges will be essential for translating mechanistic insights into clinically meaningful applications.

First, a major unmet need is the development of integrated experimental models that can simultaneously recapitulate T2D and primary colorectal tumorigenesis. Most existing studies rely on either cancer models or metabolic disease models in isolation, which limits mechanistic understanding of how hyperglycemia, insulin resistance, dyslipidemia, and chronic low-grade inflammation actively reshape lncRNA regulatory networks during CRC initiation and progression. Future studies should prioritize dual-disease *in vivo* models, such as genetically engineered CRC mouse models combined with diet-induced or genetically driven metabolic dysfunction, as well as patient-derived organoids cultured under diabetic-like metabolic conditions. These approaches would allow direct interrogation of lncRNA function under physiologically relevant metabolic stress and enable causal inference rather than associative observation.

Second, further mechanistic work is required to clarify the context-dependent and sometimes opposing roles of individual lncRNAs across different tissues and disease states. LncRNAs such as UCA1, SOX2OT, GAS5, and ZEB1-AS1 exhibit divergent functions depending on cellular context, disease stage, or metabolic status, acting as oncogenic drivers in CRC while exerting protective or adaptive roles in metabolic tissues or diabetic complications. Advanced technologies including single-cell RNA sequencing, spatial transcriptomics, and lineage-tracing approaches will be critical for dissecting tissue-specific lncRNA expression patterns, cell-type-restricted functions, and dynamic regulatory changes during disease progression.

Third, although circulating and exosomal lncRNAs show promise as non-invasive biomarkers, their clinical utility remains insufficiently validated. Large-scale, prospective clinical studies in well-characterized diabetic cohorts are urgently needed to determine whether lncRNA signatures can reliably predict CRC risk, early tumor development, prognosis, or therapeutic response. Importantly, future biomarker studies should incorporate longitudinal sampling, standardized detection platforms, and integration with metabolic, inflammatory, and genetic risk factors to improve reproducibility and clinical relevance.

Fourth, significant translational barriers remain for lncRNA-targeted therapeutics, particularly with respect to tissue-specific delivery and safety. While emerging approaches such as antisense oligonucleotides, RNA interference, CRISPR-based modulation, and nanoparticle-mediated delivery offer exciting possibilities, achieving selective targeting of colonic tumor tissue while avoiding unintended effects in metabolically active organs such as the liver, pancreas, and adipose tissue remains a major challenge. Future research should focus on developing colon-targeted delivery systems, including ligand-modified nanoparticles, microbiota-responsive carriers, and pH-sensitive formulations, as well as improving our understanding of lncRNA stability, biodistribution, and off-target effects *in vivo*.

Finally, adopting systems biology and multi-omics frameworks will be essential to capture the complexity of lncRNA-mediated regulatory networks linking metabolic dysfunction and tumorigenesis. Integrative analyses combining transcriptomics, epigenomics, proteomics, metabolomics, and microbiome data may help distinguish causative regulatory circuits from secondary effects and identify key network hubs amenable to therapeutic intervention. Such approaches will also facilitate the development of precision medicine strategies that account for metabolic status when designing CRC prevention and treatment protocols.

Collectively, addressing these challenges will substantially advance our understanding of how lncRNAs coordinate the interplay between diabetes and CRC and will accelerate the development of robust biomarkers, mechanistically informed therapeutic targets, and integrated clinical strategies for patients affected by both diseases.

## 9 Conclusion

Accumulating evidence indicates that lncRNAs play crucial roles in the molecular crosstalk between CRC and diabetes by regulating several shared signaling pathways. LncRNAs such as MALAT1, H19, ANRIL, KCNQ1OT1, MIAT, UCA1, HNF1A-AS1, ZEB1-AS1, SOX2OT, SNHG4, GAS5, and MIR31HG influence key biological processes including PI3K/AKT signaling, Wnt/β-catenin activation, inflammatory NF-κB signaling, hypoxia responses, metabolic reprogramming, angiogenesis, and epigenetic regulation. These pathways are critically involved in both metabolic dysfunction and tumor progression, highlighting the role of lncRNAs as important molecular mediators linking diabetes with colorectal carcinogenesis. The identification of shared lncRNA-regulated mechanisms provides valuable insight into the biological basis underlying the increased risk of CRC in patients with diabetes. In addition to improving our understanding of disease pathogenesis, these molecules hold promise as potential biomarkers for early detection, risk stratification, and prognosis. Moreover, targeting dysregulated lncRNAs may offer novel therapeutic opportunities to simultaneously modulate metabolic abnormalities and oncogenic signaling pathways. Overall, the growing body of research on lncRNAs emphasizes their significance in bridging metabolic disorders and cancer biology. Continued investigation into lncRNA-mediated regulatory networks will be essential for translating these findings into clinically relevant diagnostic and therapeutic strategies, ultimately contributing to improved prevention, early detection, and management of CRC in individuals with diabetes.

## References

[B1] Abdel-Megeed R. M. (2026). Current insights into long non-coding RNAs role for insulin resistance emerging in type 2 diabetes mellitus and its complications. Toxicol. Rep. 16, 102235. 10.1016/j.toxrep.2026.102235 41853659 PMC12994053

[B2] Abdulle L. E. Hao J. L. Pant O. P. Liu X. F. Zhou D. D. Gao Y. (2019). MALAT1 as a diagnostic and therapeutic target in diabetes-related complications: a promising long-noncoding RNA. Int. J. Med. Sci. 16(4):548–555. 10.7150/ijms.30097 31171906 PMC6535662

[B3] Ali Khan U. Fallah M. Sundquist K. Sundquist J. Brenner H. Kharazmi E. (2020). Risk of colorectal cancer in patients with diabetes mellitus: a Swedish nationwide cohort study. PLoS Med. 17, e1003431. 10.1371/journal.pmed.100343 33186354 PMC7665813

[B4] Amine I. M. Salsabil H. Fadil B. Adnane B. Khaoula E. (2025). The impact of ncRNAs on type 2 diabetes: a comprehensive review covering molecular mechanisms to clinical applications. Mol. Ther. Nucleic Acids 36 (3), 102629. 10.1016/j.omtn.2025.102629 40777743 PMC12329536

[B5] Amodio N. Raimondi L. Juli G. Stamato M. A. Caracciolo D. Tagliaferri P. (2018). MALAT1: a druggable long non-coding RNA for targeted anti-cancer approaches. J. Hematol. Oncol. 11 (1), 63. 10.1186/s13045-018-0606-4 29739426 PMC5941496

[B6] Arun G. Aggarwal D. Spector D. L. (2020). MALAT1 long non-coding RNA: functional implications. Noncoding RNA 6 (2), 22. 10.3390/ncrna6020022 32503170 PMC7344863

[B7] Basu K. Dey A. Kiran M. (2023). Inefficient splicing of long non-coding RNAs is associated with higher transcript complexity in human and mouse. RNA Biol. 20 (1), 563–572. 10.1080/15476286.2023.2242649 37543950 PMC10405767

[B8] Bella F. Minicozzi P. Giacomin A. Crocetti E. Federico M. Ponz de Leon M. (2013). Impact of diabetes on overall and cancer-specific mortality in colorectal cancer patients. J. Cancer Res. Clin. Oncol. 139 (8), 1303–1310. 10.1007/s00432-013-1439-8 23633003 PMC11824221

[B9] Beucher A. Miguel-Escalada I. Balboa D. De Vas M. G. Maestro M. A. Garcia-Hurtado J. (2022). The HASTER lncRNA promoter is a cis-acting transcriptional stabilizer of HNF1A. Nat. Cell. Biol. 24 (10), 1528–1540. 10.1038/s41556-022-00996-8 36202974 PMC9586874

[B10] Bhan A. Soleimani M. Mandal S. S. (2017). Long noncoding RNA and cancer: a new paradigm. Cancer Res. 77, 3965–3981. 10.1158/0008-5472.CAN-16-2634 28701486 PMC8330958

[B11] Binjawhar D. N. Alhazmi A. T. Bin Jawhar W. N. Mohammed Saeed W. Safi S. Z. (2023). Hyperglycemia-induced oxidative stress and epigenetic regulation of ET-1 gene in endothelial cells. Front. Genet. 14, 1167773. 10.3389/fgene.2023.1167773 37139232 PMC10150048

[B12] Black H. S. (2024). Oxidative stress and ROS link diabetes and cancer. J. Mol. Pathol. 5, 96–119. 10.3390/jmp5010007

[B13] Braga E. A. Fridman M. V. Filippova E. A. Loginov V. I. Pronina I. V. Burdennyy A. M. (2021). LncRNAs in the regulation of genes and signaling pathways through miRNA-mediated and other mechanisms in clear cell renal cell carcinoma. Int. J. Mol. Sci. 22 (20), 11193. 10.3390/ijms222011193 34681854 PMC8539140

[B14] Bultman S. J. (2017). Interplay between diet, gut microbiota, epigenetic events, and colorectal cancer. Mol. Nutr. Food Res. 61 (1). 10.1002/mnfr.201500902 27138454 PMC5161716

[B15] Bure I. V. Nemtsova M. V. Kuznetsova E. B. (2022). Histone modifications and non-coding RNAs: mutual epigenetic regulation and role in pathogenesis. Int. J. Mol. Sci. 23 (10), 5801. 10.3390/ijms23105801 35628612 PMC9146199

[B16] Cagle P. Qi Q. Niture S. Kumar D. (2021). KCNQ1OT1: an oncogenic long noncoding RNA. Biomolecules 11 (11), 1602. 10.3390/biom11111602 34827600 PMC8615887

[B17] Carbone F. P. Hanau S. Bianchi N. (2026). Circular and long non-coding RNAs in cancer metabolism: dual perspective of biomarkers and therapeutic targets. Noncoding RNA 12 (2), 11. 10.3390/ncrna12020011 41874139 PMC13010655

[B18] Chen B. Li J. Chi D. Sahnoune I. Calin S. Girnita L. (2019). Non-coding RNAs in IGF-1R signaling regulation: the underlying pathophysiological link between diabetes and cancer. Cells 8 (12), 1638. 10.3390/cells8121638 31847392 PMC6953109

[B19] Chen K. Yu B. Liao J. (2021). LncRNA SOX2OT alleviates mesangial cell proliferation and fibrosis in diabetic nephropathy *via* Akt/mTOR-mediated autophagy. Mol. Med. 27 (1), 71. 10.1186/s10020-021-00310-6 34238205 PMC8268185

[B20] Chen Y. Meng Z. Li Y. Liu S. Hu P. Luo E. (2024). Advanced glycation end products and reactive oxygen species: uncovering the potential role of ferroptosis in diabetic complications. Mol. Med. 30 (1), 141. 10.1186/s10020-024-00905-9 39251935 PMC11385660

[B21] Chen Y. Zhang J. Ding Y. Zhu F. Chen Y. (2026a). Colorectal cancer pathogenesis, oncogenic signaling networks and targeted therapeutic advances. Mol. Biomed. 7 (1), 32. 10.1186/s43556-026-00433-4 41826571 PMC12988139

[B22] Chen S. Cao Z. Chen W. Zhao J. Jiao L. Prettner K. (2026b). The global macroeconomic burden of diabetes mellitus. Nat. Med. 32 (1), 126–138. 10.1038/s41591-025-04027-5 41466063 PMC12823416

[B23] Cheng Y. Wu X. Xia Y. Liu W. Wang P. (2022). The role of lncRNAs in regulation of DKD and diabetes-related cancer. Front. Oncol. 12, 1035487. 10.3389/fonc.2022.1035487 36313695 PMC9606714

[B24] Chodurska B. Kunej T. (2025). Long non-coding RNAs in humans: classification, genomic organization and function. Noncoding RNA Res. 11, 313–327. 10.1016/j.ncrna.2025.01.004 39967600 PMC11833636

[B25] Chowdhury P. R. Salvamani S. Gunasekaran B. Peng H. B. Ulaganathan V. (2023). H19: an oncogenic long non-coding RNA in colorectal cancer. Yale J. Biol. Med. 96 (4), 495–509. 10.59249/TDBJ7410 38161577 PMC10751868

[B26] Chu Q. Gu X. Zheng Q. Guo Z. Shan D. Wang J. (2021). Long noncoding RNA SNHG4: a novel target in human diseases. Cancer Cell. Int. 21, 583. 10.1186/s12935-021-02292-1 34717631 PMC8557547

[B27] Congrains A. Kamide K. Ohishi M. Rakugi H. (2013). ANRIL: molecular mechanisms and implications in human health. Int. J. Mol. Sci. 14 (1), 1278–1292. 10.3390/ijms14011278 23306151 PMC3565320

[B28] Costa S. La Rocca G. Cavalieri V. (2025). Epigenetic regulation of chromatin functions by microRNAs and long noncoding RNAs and implications in human diseases. Biomedicines 13 (3), 725. 10.3390/biomedicines13030725 40149701 PMC11939841

[B29] Cuevas-Diaz Duran R. Wei H. Kim D. H. Wu J. Q. (2019). Long non-coding RNAs: important regulators in the development, function and disorders of the central nervous system. Neuropathol. Appl. Neurobiol. 45 (6), 538–556. 10.1111/nan.12541 30636336 PMC6626588

[B30] Dai C. Qianjiang H. Fu R. Yang H. Shi A. Luo H. (2025). Epigenetic and epitranscriptomic role of lncRNA in carcinogenesis (review). Int. J. Oncol. 66 (4), 29. 10.3892/ijo.2025.5735 40017127 PMC11900940

[B31] Davidovich C. Cech T. R. (2015). The recruitment of chromatin modifiers by long noncoding RNAs: lessons from PRC2. RNA 21 (12), 2007–2022. 10.1261/rna.053918.115 26574518 PMC4647455

[B32] DeForest N. Kavitha B. Hu S. Isaac R. Krohn L. Wang M. (2023). Human gain-of-function variants in HNF1A confer protection from diabetes but independently increase hepatic secretion of atherogenic lipoproteins. Cell. Genom 3, 100346. 10.1016/j.xgen.2023.100339 37492105 PMC10363808

[B33] Dehghanzad R. Rahbar Parvaneh R. Keramatipour M. Kadkhoda S. Aghajanpour M. Taslimi R. (2024). Dysregulated long non-coding RNAs in colorectal cancer: identification and validation using RNA-seq and real-time reverse transcription polymerase chain reaction. Cell. J. (Yakhteh) 26 (12), 700–710. 10.22074/cellj.2025.2042114.1684 40600321

[B34] Diamantopoulos M. A. Boti M. A. Sarri T. Scorilas A. (2025). Non-coding RNAs in health and disease: from biomarkers to therapeutic targets. Lab. Med. 2, 17. 10.1016/j.labmed.2024.100012

[B35] Ding B. Wang X. Zhang Z. Wang Y. Bo W. Zhang M. (2025a). Interactions between lncRNAs and MAPK signaling pathways in the pathogenesis of breast cancer. Cancer Cell. Int. 25 (1), 339. 10.1186/s12935-025-03943-3 41053774 PMC12502614

[B36] Ding Y. Zheng S. Fu X. N. Ma C. Juan Y. (2025b). LncRNA SNHG4 regulates lipid metabolism and inflammation in non-alcoholic fatty liver disease by targeting the miR-34b-5p/XIAP axis. Turk J. Gastroenterol. 36 (10), 629–640. 10.5152/tjg.2025.24191 41084767 PMC12520139

[B37] Dodangeh F. Sadeghi Z. Maleki P. Raheb J. (2023). Long non-coding RNA SOX2-OT enhances cancer biological traits *via* sponging tumor suppressors miR-122-3p and miR-194-5p in non-small cell lung carcinoma. Sci. Rep. 13, 12371. 10.1038/s41598-023-39000-0 37524903 PMC10390639

[B38] Dong Q. Qiu H. Piao C. Li Z. Cui X. (2023). LncRNA SNHG4 promotes prostate cancer cell survival and resistance to enzalutamide through a let-7a/RREB1 positive feedback loop and a ceRNA network. J. Exp. Clin. Cancer Res. 42, 209. 10.1186/s13046-023-02774-2 37596700 PMC10436424

[B39] Duan Q. Cai L. Zheng K. Cui C. Huang R. Zheng Z. (2020). LncRNA KCNQ1OT1 knockdown inhibits colorectal cancer cell proliferation, migration and invasiveness *via* the PI3K/AKT pathway. Oncol. Lett. 20 (1), 601–610. 10.3892/ol.2020.11619 32565985 PMC7286112

[B40] Fan N. Fu H. Feng X. Chen Y. Wang J. Wu Y. (2022). Long non-coding RNAs play an important regulatory role in tumorigenesis and tumor progression through aerobic glycolysis. Front. Mol. Biosci. 9, 941653. 10.3389/fmolb.2022.941653 36072431 PMC9441491

[B41] Fang C. Qiu S. Sun F. Li W. Wang Z. Yue B. (2017). Long non-coding RNA HNF1A-AS1-mediated repression of the miR-34a/SIRT1/p53 feedback loop promotes the metastatic progression of colon cancer by functioning as a competing endogenous RNA. Cancer Lett. 410, 50–62. 10.1016/j.canlet.2017.09.012 28943452

[B42] Farooqi A. A. Fayyaz S. Poltronieri P. Calin G. Mallardo M. (2022). Epigenetic deregulation in cancer: enzyme players and non-coding RNAs. Semin. Cancer Biol. 83, 197–207. 10.1016/j.semcancer.2020.07.013 32738290

[B43] Feng Y. Xu Y. Gao Y. Chen Y. Wang X. Chen Z. (2021). A novel lncRNA SOX2OT promotes the malignancy of human colorectal cancer by interacting with the miR-194-5p/SOX5 axis. Cell. Death Dis. 12 (5), 499. 10.1038/s41419-021-03756-y 33993197 PMC8124073

[B44] Feng J. Hu S. Liu K. Sun G. Zhang Y. (2022). The role of microRNA in the regulation of tumor epithelial–mesenchymal transition. Cells 11 (13), 1981. 10.3390/cells11131981 35805066 PMC9265548

[B45] Ferbus D. Bovin C. Validire P. Goubin G. (2003). The zinc finger protein OZF (ZNF146) is overexpressed in colorectal cancer. J. Pathol. 200 (2), 177–182. 10.1002/path.1337 12754738

[B46] Ferrer J. Dimitrova N. (2024). Transcription regulation by long non-coding RNAs: mechanisms and disease relevance. Nat. Rev. Mol. Cell. Biol. 25 (5), 396–415. 10.1038/s41580-023-00694-9 38242953 PMC11045326

[B47] Gaggi G. Hausman C. Cho S. Badalamenti B. C. Trinh B. Q. Di Ruscio A. (2025). LncRNAs ride the storm of epigenetic marks. Genes. (Basel) 16 (3), 313. 10.3390/genes16030313 40149464 PMC11942515

[B48] Gallagher E. J. LeRoith D. (2010). The proliferating role of insulin and insulin-like growth factors in cancer. Trends Endocrinol. Metab. 21 (10), 610–618. 10.1016/j.tem.2010.06.007 20663687 PMC2949481

[B49] Garcia-Villatoro E. L. DeLuca J. A. Callaway E. S. Allred K. F. Davidson L. A. Hensel M. E. (2020). Effects of high-fat diet and intestinal aryl hydrocarbon receptor deletion on colon carcinogenesis. Am. J. Physiol. Gastrointest. Liver Physiol. 318 (3), G451–G463. 10.1152/ajpgi.00268.2019 31905023 PMC7137094

[B50] Ghafouri-Fard S. Taheri M. (2019). UCA1 long non-coding RNA: an update on its roles in malignant behavior of cancers. Biomed. Pharmacother. 120, 109459. 10.1016/j.biopha.2019.109459 31585301

[B51] Ghafouri-Fard S. Askari A. Moghadam K. B. Hussen B. M. Taheri M. Samadian M. (2023). A review on the role of ZEB1-AS1 in human disorders. Pathol. Res. Pract. 245, 154486. 10.1016/j.prp.2023.154486 37120907

[B52] Ghahramani A. H. Karimi B. Valizadeh S. Ghaedi K. (2025). Biological functions and affected signaling pathways by long non-coding RNAs in the immune system. Noncoding RNA Res. 10, 70–90. 10.1016/j.ncrna.2024.09.001 39315339 PMC11417496

[B53] Glaviano A. Foo A. S. C. Lam H. Y. Yap K. C. H. Jacot W. Jones R. H. (2023). PI3K/AKT/mTOR signaling transduction pathway and targeted therapies in cancer. Mol. Cancer 22 (1), 138. 10.1186/s12943-023-01827-6 37596643 PMC10436543

[B54] Grega T. Vojtechova G. Gregova M. Zavoral M. Suchanek S. (2021). Pathophysiological characteristics linking type 2 diabetes mellitus and colorectal neoplasia. Physiol. Res. 70 (4), 509–522. 10.33549/physiolres.934631 34062073 PMC8820551

[B55] Groeneweg K. E. Au Y. W. Duijs J. M. Florijn B. W. van Kooten C. de Fijter J. W. (2020). Diabetic nephropathy alters circulating long noncoding RNA levels that normalize following simultaneous pancreas–kidney transplantation. Am. J. Transpl. 20 (12), 3451–3461. 10.1111/ajt.15961 32353171 PMC7754299

[B56] Groulx I. Lee S. (2002). Oxygen-dependent ubiquitination and degradation of hypoxia-inducible factor requires nuclear-cytoplasmic trafficking of the von Hippel-Lindau tumor suppressor protein. Mol. Cell. Biol. 22 (15), 5319–5336. 10.1128/MCB.22.15.5319-5336.2002 12101228 PMC133938

[B57] Gu L. Sun H. Yan Z. (2020). LncRNA ZEB1-AS1 is downregulated in diabetic lung and regulates lung cell apoptosis. Exp. Ther. Med. 20 (6), 225. 10.3892/etm.2020.9355 33193839 PMC7646694

[B58] Guha P. Chini A. Rishi A. Mandal S. S. (2024). Long noncoding RNAs in ubiquitination, protein degradation, and human diseases. Biochim. Biophys. Acta Gene Regul. Mech. 1867 (4), 195061. 10.1016/j.bbagrm.2024.195061 39341591 PMC12874621

[B59] Guo X. Zhang Y. Liu L. Yang W. Zhang Q. (2020). HNF1A-AS1 regulates cell migration, invasion, and glycolysis *via* modulating miR-124/MYO6 in colorectal cancer cells. Onco Targets Ther. 13, 1507–1518. 10.2147/OTT.S231249 32110048 PMC7035897

[B60] Guo T. Liu D. Peng S. Wang M. Li Y. (2021). A positive feedback loop of lncRNA MIR31HG-miR-361-3p-YY1 accelerates colorectal cancer progression by modulating proliferation, angiogenesis, and glycolysis. Front. Oncol. 11, 684984. 10.3389/fonc.2021.684984 34485123 PMC8416113

[B61] Gupta S. C. Awasthee N. Rai V. Chava S. Gunda V. Challagundla K. B. (2020). Long non-coding RNAs and nuclear factor-κB crosstalk in cancer and other human diseases. Biochim. Biophys. Acta Rev. Cancer 873 (1), 188316. 10.1016/j.bbcan.2019.188316 31639408 PMC7775411

[B62] Han P. Chang C. P. (2015). Long non-coding RNA and chromatin remodeling. RNA Biol. 12 (10), 1094–1098. 10.1080/15476286.2015.1063770 26177256 PMC4829272

[B63] Hashemi M. Mohandesi Khosroshahi E. Asadi S. Tanha M. Ghatei Mohseni F. Abdolmohammad S. R. (2025). Emerging roles of non-coding RNAs in modulating the PI3K/Akt pathway in cancer. Noncoding RNA Res. 10, 1–15. 10.1016/j.ncrna.2024.08.001 39296640 PMC11406677

[B64] He K. Gan W. J. (2023). Wnt/β-catenin signaling pathway in the development and progression of colorectal cancer. Cancer Manag. Res. 15, 435–448. 10.2147/CMAR.S411168 37250384 PMC10224676

[B65] Hernández-Aguilar A. I. Luciano-Villa C. A. Tello-Flores V. A. Beltrán-Anaya F. O. Zubillaga-Guerrero M. I. Flores-Alfaro E. (2021). Dysregulation of lncRNA-H19 in cardiometabolic diseases and the molecular mechanism involved: a systematic review. Expert Rev. Mol. Diagn 21 (8), 809–821. 10.1080/14737159.2021.1944808 34133256

[B66] Hu X. Y. Hou P. F. Li T. T. Quan H. Y. Li M. L. Lin T. (2018). The roles of Wnt/β-catenin signaling pathway-related lncRNAs in cancer. Int. J. Biol. Sci. 14 (14), 2003–2011. 10.7150/ijbs.27977 30585264 PMC6299370

[B67] Huang Y. Jin C. Zheng Y. Li X. Zhang S. Zhang Y. (2017). Knockdown of lncRNA MIR31HG inhibits adipocyte differentiation of human adipose-derived stem cells *via* histone modification of FABP4. Sci. Rep. 7 (1), 8080. 10.1038/s41598-017-08131-6 28808264 PMC5556051

[B68] Huang K. Yu X. Yu Y. Zhang L. Cen Y. Chu J. (2020). Long noncoding RNA MALAT1 promotes high glucose-induced inflammation and apoptosis of vascular endothelial cells by regulating miR-361-3p/SOCS3 axis. Int. J. Clin. Exp. Pathol. 13 (5), 1243–1252. 32509100 PMC7270668

[B69] Huang S. Wu K. Li B. Liu Y. (2023). LncRNA UCA1 inhibits mitochondrial dysfunction of skeletal muscle in type 2 diabetes mellitus by sequestering miR-143-3p to release FGF21. Cell. Tissue Res. 391 (3), 561–575. 10.1007/s00441-022-03733-7 36602629

[B70] Indira M. Abhilash P. (2013). Role of NF-kappa B (NF-κB) in diabetes. Oncother Ther. 4 (2), 111–132. 10.1615/ForumImmunDisTher.2013008396

[B71] Jasim S. A. Pallathadka H. Hjazi A. Sanghvi G. Mr M. Agarwal M. (2025). LncRNA–histone modification crosstalk: orchestrating cancer pathobiology. Naunyn Schmiedeb. Arch. Pharmacol. 398 (12), 16641–16660. 10.1007/s00210-025-04402-6 40643648

[B72] Jiang X. Li J. Wang W. Hu Z. Guan C. Zhao Y. (2020). AR-induced ZEB1-AS1 represents poor prognosis in cholangiocarcinoma and facilitates tumor stemness, proliferation and invasion by mediating the miR-133b/HOXB8 axis. Aging (Albany NY) 12 (2), 1237–1255. 10.18632/aging.102680 31978895 PMC7053610

[B73] Jin Z. Chen B. (2020). LncRNA ZEB1-AS1 regulates colorectal cancer cells *via* the miR-205/YAP1 axis. Open Med. (Wars) 15, 175–184. 10.1515/med-2020-0026 32190742 PMC7065425

[B74] Karakas D. Ozpolat B. (2021). The role of lncRNAs in translation. Noncoding RNA 7 (1), 16. 10.3390/ncrna7010016 33672592 PMC8005997

[B75] Kong Y. Hsieh C. H. Alonso L. C. (2018). ANRIL: a lncRNA at the CDKN2A/B locus with roles in cancer and metabolic disease. Front. Endocrinol. (Lausanne) 9, 405. 10.3389/fendo.2018.00405 30087655 PMC6066557

[B76] Lecarpentier Y. Claes V. Vallée A. Hébert J. L. (2017). Interactions between PPAR gamma and the canonical Wnt/β-catenin pathway in type 2 diabetes and colon cancer. PPAR Res. 2017, 5879090. 10.1155/2017/5879090 28298922 PMC5337359

[B77] Li Z. Li X. Wu S. Xue M. Chen W. (2014). Long non-coding RNA UCA1 promotes glycolysis by upregulating hexokinase 2 through the mTOR–STAT3/microRNA-143 pathway. Cancer Sci. 105, 951–955. 10.1111/cas.12461 24890811 PMC4317864

[B78] Li P. Zhang X. Wang H. Wang L. Liu T. Du L. (2017a). MALAT1 is associated with poor response to oxaliplatin-based chemotherapy in colorectal cancer patients and promotes chemoresistance through EZH2. Mol. Cancer Ther. 16 (4), 739–751. 10.1158/1535-7163 28069878

[B79] Li C. P. Wang S. H. Wang W. Q. Song S. G. Liu X. M. (2017b). Long noncoding RNA Sox2OT knockdown alleviates diabetes mellitus-induced retinal ganglion cell injury. Cell. Mol. Neurobiol. 37 (2), 361–369. 10.1007/s10571-016-0380-1 27193103 PMC11482052

[B80] Li L. Dai Y. Ke D. Liu J. Chen P. Wei D. (2023). Ferroptosis: new insight into the mechanisms of diabetic nephropathy and retinopathy. Front. Endocrinol. (Lausanne) 14, 1215292. 10.3389/fendo.2023.1215292 37600716 PMC10435881

[B81] Li L. Wu Y. Q. Yang J. E. (2025). Stress-related lncRNAs and their roles in diabetes and diabetic complications. Int. J. Mol. Sci. 26(5):2194. 10.3390/ijms26052194 40076814 PMC11900361

[B82] Liao J. Chen B. Zhu Z. Du C. Gao S. Zhao G. (2023). Long noncoding RNA (lncRNA) H19: an essential developmental regulator with expanding roles in cancer, stem cell differentiation, and metabolic diseases. Genes. Dis. 10 (4), 1351–1366. 10.1016/j.gendis.2023.02.008 37397543 PMC10311118

[B83] Lin W. Zhou Q. Wang C. Q. Zhu L. Bi C. Zhang S. (2020). LncRNAs regulate metabolism in cancer. Int. J. Biol. Sci. 16, 1194–1206. 10.7150/ijbs.40713 32174794 PMC7053319

[B84] Lin Z. B. Long P. Zhao Z. Zhang Y. R. Chu X. D. Zhao X. X. (2021). Long noncoding RNA KCNQ1OT1 is a prognostic biomarker and mediates CD8(+) T cell exhaustion by regulating CD155 expression in colorectal cancer. Int. J. Biol. Sci. 17 (7), 1757–1768. 10.7150/ijbs.59001 33994860 PMC8120463

[B85] Liu S. Xu B. Yan D. (2016). Enhanced expression of long non-coding RNA SOX2OT promotes cell proliferation and motility in colorectal cancer. Minerva Med. 107 (5), 279–286. 27353770

[B86] Liu Z. Wang H. Cai H. Hong Y. Li Y. Su D. (2018). Long non-coding RNA MIAT promotes growth and metastasis of colorectal cancer cells through regulation of miR-132/Derlin-1 pathway. Cancer Cell. Int. 18, 59. 10.1186/s12935-017-0477-8 29686537 PMC5902964

[B87] Liu H. T. Ma R. R. Lv B. B. Zhang H. Shi D. B. Guo X. Y. (2020a). LncRNA HNF1A-AS1 functions as a competing endogenous RNA to activate the PI3K/AKT signalling pathway by sponging miR-30b-3p in gastric cancer. Br. J. Cancer 122, 1825–1836. 10.1038/s41416-020-0836-4 32336754 PMC7283217

[B88] Liu B. Zhou J. Wang C. Chi Y. Wei Q. Fu Z. (2020b). LncRNA SOX2OT promotes temozolomide resistance by elevating SOX2 expression *via* ALKBH5-mediated epigenetic regulation in glioblastoma. Cell. Death Dis. 11 (5), 384. 10.1038/s41419-020-2540-y 32439916 PMC7242335

[B89] Liu J. Lv W. Li S. Deng J. (2021a). Regulation of long non-coding RNA KCNQ1OT1 network in colorectal cancer immunity. Front. Genet. 12, 684002. 10.3389/fgene.2021.684002 34630508 PMC8493092

[B90] Liu Z. Wang Y. Yuan S. Wen F. Liu J. Zou L. (2021b). Regulatory role of long non-coding RNA UCA1 in signaling pathways and its clinical applications. Oncol. Lett. 21, 404. 10.3892/ol.2021.12665 33777227 PMC7988699

[B91] Liu G. Shi L. Wang B. Wu Z. Zhao H. Zhao T. (2024a). Role of oncogenic long noncoding RNA KCNQ1OT1 in colon cancer. Oncol. Res. 32 (3), 585–596. 10.32604/or.2023.029349 38361755 PMC10865742

[B92] Liu H. Zhang H. Lou H. Wang J. Hao S. Chen H. (2024b). ZEB1-AS1 as a TRPML1 inhibitor causes lysosome dysfunction and cardiac damage in aged mice. Eng. (Beijing) 43, 183–200. 10.1016/j.eng.2024.09.020

[B93] Loganathan T. Doss C. G. (2023). Non-coding RNAs in human health and disease: potential function as biomarkers and therapeutic targets. Funct. Integr. Genomics. 33. 10.1007/s10142-022-00943-7 PMC9838419 36625940

[B94] Long Y. Wang X. Youmans D. T. Cech T. R. (2017). How do lncRNAs regulate transcription? Sci. Adv. 3 (9), eaao2110. 10.1126/sciadv.aao2110 28959731 PMC5617379

[B95] Lu X. Xie Q. Pan X. Zhang R. Zhang X. Peng G. (2024). Type 2 diabetes mellitus in adults: pathogenesis, prevention and therapy. Signal Transduct. Target Ther. 9, 262. 10.1038/s41392-024-01968-4 39353925 PMC11445387

[B96] Luan Y. Li X. Luan Y. Zhao R. Li Y. Liu L. (2020). Circulating lncRNA UCA1 promotes malignancy of colorectal cancer *via* the miR-143/MYO6 axis. Mol. Ther. Nucleic Acids 19, 790–803. 10.1016/j.omtn.2019.12.009 31955010 PMC6970172

[B97] Luo Y. Guo J. Xu P. Gui R. (2020). Long non-coding RNA GAS5 maintains insulin secretion by regulating multiple miRNAs in INS-1 832/13 cells. Front. Mol. Biosci. 7, 559267. 10.3389/fmolb.2020.559267 33195407 PMC7542228

[B98] Lv S. Y. Shan T. D. Pan X. T. Tian Z. B. Liu X. S. Liu F. G. (2018). The lncRNA ZEB1-AS1 sponges miR-181a-5p to promote colorectal cancer cell proliferation by regulating Wnt/β-catenin signaling. Cell. Cycle 17 (10), 1245–1254. 10.1080/15384101.2018.1471317 29886791 PMC6110576

[B99] Lv L. Huang B. Yi L. Zhang L. (2023). Long non-coding RNA SNHG4 enhances RNF14 mRNA stability to promote the progression of colorectal cancer by recruiting TAF15 protein. Apoptosis 28, 414–431. 10.1007/s10495-022-01781-6 36482019

[B100] Ma W. Hu J. (2023). The linear ANRIL transcript P14AS regulates the NF-κB signaling to promote colon cancer progression. Mol. Med. 29 (1), 162. 10.1186/s10020-023-00761-z 38041015 PMC10690983

[B101] Ma B. Wang S. Wu W. Shan P. Chen Y. Meng J. (2023). Mechanisms of circRNA/lncRNA-miRNA interactions and applications in disease and drug research. Biomed. Pharmacother. 162, 114672. 10.1016/j.biopha.2023.114672 37060662

[B102] Maeda T. Konishi H. Takaki W. Takabatake K. Matsubara D. Shoda K. (2021). Significance of plasma UCA1 for predicting colorectal cancer and BRAF mutation status. Anticancer Res. 41 (4), 1761–1769. 10.21873/anticanres.14941 33813380

[B103] Maharati A. Moghbeli M. (2023). Long non-coding RNAs as critical regulators of PI3K/AKT, TGF-β, and MAPK signaling pathways during breast tumor progression. J. Transl. Med. 21 (1), 556. 10.1186/s12967-023-04434-7 37596669 PMC10439650

[B104] Manzoor S. Muhammad J. S. Maghazachi A. A. Hamid Q. (2022). Autophagy: a versatile player in the progression of colorectal cancer and drug resistance. Front. Oncol. 12, 924290. 10.3389/fonc.2022.924290 35912261 PMC9329589

[B105] Maschio D. Oliveira R. Santos M. Carvalho C. Barbosa-Sampaio H. Collares-Buzato C. (2016). Activation of the Wnt/β-catenin pathway in pancreatic beta cells during compensatory islet hyperplasia in prediabetic mice. Biochem. Biophys. Res. Commun. 478 (4), 1534–1540. 10.1016/j.bbrc.2016.08.146 27576200

[B106] Mattick J. S. Amaral P. P. Carninci P. Carpenter S. Chang H. Y. Chen L. L. (2023). Long non-coding RNAs: definitions, functions, challenges and recommendations. Nat. Rev. Mol. Cell. Biol. 24, 430–447. 10.1038/s41580-022-00566-8 36596869 PMC10213152

[B107] Mehmandar-Oskuie A. Jahankhani K. Rostamlou A. Mardafkan N. Karamali N. Razavi Z. S. (2024). Molecular mechanism of lncRNAs in pathogenesis and diagnosis of autoimmune diseases, with a special focus on lncRNA-based therapeutic approaches. Life Sci. 336, 122322. 10.1016/j.lfs.2023.122322 38042283

[B108] Morlando M. Fatica A. (2018). Alteration of epigenetic regulation by long noncoding RNAs in cancer. Int. J. Mol. Sci. 19 (2), 570. 10.3390/ijms19020570 29443889 PMC5855792

[B109] Mu M. Zhang Q. Li J. Zhao C. Li X. Chen Z. (2023). USP51 facilitates colorectal cancer stemness and chemoresistance by forming a positive feed-forward loop with HIF1A. Cell. Death Differ. 30 (11), 2393–2407. 10.1038/s41418-023-01228-8 37816999 PMC10657471

[B110] Neve B. Jonckheere N. Vincent A. Van Seuningen I. (2018). Epigenetic regulation by lncRNAs: an overview focused on UCA1 in colorectal cancer. Cancers (Basel) 10 (11), 440. 10.3390/cancers10110440 30441811 PMC6266399

[B111] Nguyen L. N. T. Pyburn J. S. Nguyen N. L. Schank M. B. Zhao J. Wang L. (2025). Epigenetic regulation by the lncRNA GAS5/miRNA/mRNA network in human diseases. Int. J. Mol. Sci. 26 (3), 1377. 10.3390/ijms26031377 39941145 PMC11818527

[B112] Ni W. Yao S. Zhou Y. Liu Y. Huang P. Zhou A. (2019). Long noncoding RNA GAS5 inhibits progression of colorectal cancer by interacting with and triggering YAP phosphorylation and degradation and is negatively regulated by the m6A reader YTHDF3. Mol. Cancer 18 (1), 143. 10.1186/s12943-019-1079-y 31619268 PMC6794841

[B113] Nie Z. Zhu H. Gu M. (2016). Reduced colorectal cancer incidence in type 2 diabetic patients treated with metformin: a meta-analysis. Pharm. Biol. 54 (11), 2636–2642. 10.1080/13880209.2016.1176057 27159666

[B114] Otake S. Itoh Y. Omata C. Saitoh M. Miyazawa K. (2021). ZEB1 and oncogenic Ras constitute a regulatory switch for stimulus-dependent E-cadherin downregulation. Cancer Sci. 112 (1), 205–216. 10.1111/cas.14701 33068045 PMC7780036

[B115] Pei D. Zhang D. Guo Y. Chang H. Cui H. (2025). Long non-coding RNAs in malignant human brain tumors: driving forces behind progression and therapy. Int. J. Mol. Sci. 26, 712. 10.3390/ijms26020712 39859408 PMC11766336

[B116] Peng X. Li S. Zeng A. Song L. (2024). Regulatory function of glycolysis-related lncRNAs in tumor progression: mechanism, facts, and perspectives. Biochem. Pharmacol. 229, 116511. 10.1016/j.bcp.2024.116511 39222714

[B117] Perez-Oquendo M. Gibbons D. L. (2022). Regulation of ZEB1 function and molecular associations in tumor progression and metastasis. Cancers (Basel) 14 (8), 1864. 10.3390/cancers14081864 35454770 PMC9031734

[B118] Perisset S. Potilinski M. C. Gallo J. E. (2023). Role of lnc-RNAs in the pathogenesis and development of diabetic retinopathy. Int. J. Mol. Sci. 24 (18), 13947. 10.3390/ijms241813947 37762249 PMC10531058

[B119] Pullen T. J. Rutter G. A. (2014). Roles of lncRNAs in pancreatic beta cell identity and diabetes susceptibility. Front. Genet. 5, 193. 10.3389/fgene.2014.00193 25071823 PMC4076741

[B120] Rajendran P. Sekar R. Abdallah B. M. Jh S. F. Ali E. M. Jayaraman S. (2024). Epigenetic modulation of long noncoding RNA H19 in oral squamous cell carcinoma: a narrative review. Noncoding RNA Res. 9 (2), 602–611. 10.1016/j.ncrna.2024.01.020 38532798 PMC10963247

[B121] Ramasubbu K. Devi R. V. (2023). Impairment of insulin signaling pathway PI3K/Akt/mTOR and insulin resistance induced AGEs on diabetes mellitus and neurodegenerative diseases: a perspective review. Mol. Cell. Biochem. 478 (6), 1307–1324. 10.1007/s11010-022-04587-x 36308670

[B122] Ramli S. Sim M. S. Guad R. M. Gopinath S. C. B. Subramaniyan V. Fuloria S. (2021). Long noncoding RNA UCA1 in gastrointestinal cancers: molecular regulatory roles and patterns, mechanisms, and interactions. J. Oncol. 2021, 5519720. 10.1155/2021/5519720 33936199 PMC8055404

[B123] Ran G. Zhu X. Qin Y. (2021). LncRNA SOX2OT is upregulated in gestational diabetes mellitus and correlated with multiple adverse events. Diabetes Metab. Syndr. Obes. 14, 3989–3995. 10.2147/DMSO.S319739 34531671 PMC8439441

[B124] Rawla P. Sunkara T. Barsouk A. (2019). Epidemiology of colorectal cancer: incidence, mortality, survival, and risk factors. Prz. Gastroenterol. 14, 89–103. 10.5114/pg.2018.81072 31616522 PMC6791134

[B125] Ren Y. Zhang Z. Lei X. Shi L. (2025). Long non-coding RNAs in cancer glycolysis and metabolism: mechanisms and translational opportunities. Cell. Death Dis. 17, 71. 10.1038/s41419-025-08289-2 41361062 PMC12827984

[B126] Rostom M. M. Rashwan A. A. Sotiropoulou C. D. Hozayen S. Z. Abdelhamid A. M. Abdelhalim M. M. (2025). MIAT: a pivotal oncogenic long noncoding RNA tuning the hallmarks of solid malignancies. Transl. Oncol. 54, 102329. 10.1016/j.tranon.2025.102329 40014977 PMC11910686

[B127] Ruan L. Lei J. Yuan Y. Li H. Yang H. Wang J. (2023). MIR31HG, a potential lncRNA in human cancers and non-cancers. Front. Genet. 14, 1145454. 10.3389/fgene.2023.1145454 37636269 PMC10449471

[B128] Ruiz-Malagón A. J. Rodríguez-Sojo M. J. Redondo E. Rodríguez-Cabezas M. E. Gálvez J. Rodríguez-Nogales A. (2025). Systematic review: the gut microbiota as a link between colorectal cancer and obesity. Obes. Rev. 26 (4), e13872. 10.1111/obr.13872 39614602 PMC11884970

[B129] Sepehri Z. Banerjee A. Vizeacoumar F. S. Freywald A. Vizeacoumar F. J. Dolinsky V. W. (2022). Differential expression of HNF1A and HNF1A-AS1 in colon cancer cells. IUBMB Life 74 (6), 496–507. 10.1002/iub.2609 35184384

[B130] Shahryari A. Jazi M. S. Samaei N. M. Mowla S. J. (2015). Long non-coding RNA SOX2OT: expression signature, splicing patterns, and emerging roles in pluripotency and tumorigenesis. Front. Genet. 6, 196. 10.3389/fgene.2015.00196 26136768 PMC4469893

[B131] Shan T. D. Tian Z. B. Jiang Y. P. (2020). Downregulation of lncRNA MALAT1 suppresses abnormal proliferation of small intestinal epithelial stem cells through miR-129-5p expression in diabetic mice. Int. J. Mol. Med. 45 (4), 1250–1260. 10.3892/ijmm.2020.4492 32124944

[B132] Shen J. Zhao X. Zhong Y. Yang P. Gao P. Wu X. (2022). Exosomal ncRNAs: the pivotal players in diabetic wound healing. Front. Immunol. 13, 1005307. 10.3389/fimmu.2022.1005307 36420273 PMC9677725

[B133] Shi C. H. Huang Y. Li W. Q. Chen R. G. (2019a). Influence of lncRNA UCA1 on glucose metabolism in rats with diabetic nephropathy through PI3K–Akt signaling pathway. Eur. Rev. Med. Pharmacol. Sci. 23 (22), 10058–10064. 10.26355/eurrev_201911_19573 31799676

[B134] Shi Y. Parag S. Patel R. Lui A. Murr M. Cai J. (2019b). Stabilization of lncRNA GAS5 by a small molecule and its implications in diabetic adipocytes. Cell. Chem. Biol. 26 (3), 319–330.e6. 10.1016/j.chembiol.2018.11.012 30661991 PMC10498384

[B135] Shin C. H. Kim K. Ho C. W. Lee J. W. Jo M. J. Min K. W. (2025). Long noncoding RNAs regulating enzymatic reactions in cancer. Exp. Mol. Med. 57, 1641–1650. 10.1038/s12276-024-01311-2 40804479 PMC12411633

[B136] Song Y. Miao C. Wang J. (2019). LncRNA ZEB1-AS1 inhibits renal fibrosis in diabetic nephropathy by regulating the miR-217/MAFB axis. RSC Adv. 9 (52), 30389–30397. 10.1039/c9ra05602e 35557748 PMC9088285

[B137] Su W. Xu M. Chen X. Chen N. Gong J. Nie L. (2017). Long noncoding RNA ZEB1-AS1 epigenetically regulates the expression of ZEB1 and downstream molecules in prostate cancer. Mol. Cancer 16 (1), 142. 10.1186/s12943-017-0711-y 28830551 PMC5568204

[B138] Tang J. Chen Z. Wang Q. Hao W. Gao W. Q. Xu H. (2021). hnRNPA2B1 promotes colon cancer progression *via* the MAPK pathway. Front. Genet. 12, 666451. 10.3389/fgene.2021.666451 34630502 PMC8494201

[B139] Tello-Flores V. A. Beltrán-Anaya F. O. Ramírez-Vargas M. A. Esteban-Casales B. E. Navarro-Tito N. Alarcón-Romero L. D. C. (2021). Role of long non-coding RNAs and the molecular mechanisms involved in insulin resistance. Int. J. Mol. Sci. 22, 7533. 10.3390/ijms22147533 34298896 PMC8306787

[B140] Thomas A. A. Feng B. Chakrabarti S. (2017). ANRIL: a regulator of VEGF in diabetic retinopathy. Invest. Ophthalmol. Vis. Sci. 58 (1), 470–480. 10.1167/iovs.16-20569 28122089

[B141] Tudor L. Konjevod M. Nedic Erjavec G. Nikolac Perkovic M. Uzun S. Kozumplik O. (2022). Genetic and epigenetic association of hepatocyte nuclear factor-1α with glycosylation in post-traumatic stress disorder. Genes. (Basel). 13, 1063. 10.3390/genes13061063 35741825 PMC9223288

[B142] Varney M. J. Benovic J. L. (2024). The role of G protein-coupled receptors and receptor kinases in pancreatic β-cell function and diabetes. Pharmacol. Rev. 76 (2), 267–299. 10.1124/pharmrev.123.001015 38351071 PMC10877731

[B143] Vekic J. Zeljkovic A. Stefanovic A. Giglio R. V. Ciaccio M. Rizzo M. (2021). Diabetes and colorectal cancer risk: a new look at molecular mechanisms and potential role of novel antidiabetic agents. Int. J. Mol. Sci. 22, 12440. 10.3390/ijms222212440 34830295 PMC8622770

[B144] Vienot A. Vernerey D. Bouard A. Klajer E. Kim S. Tournigand C. (2025). Stanniocalcin 1 in patients with refractory colorectal cancer treated with regorafenib: a post hoc biomarker analysis of the TEXCAN and CORRECT trials. Cancer Res. Commun. 5 (2), 287–294. 10.1158/2767-9764.CRC-24-0246 39807836 PMC11811826

[B145] Wang K. C. Chang H. Y. (2011). Molecular mechanisms of long noncoding RNAs. Mol. Cell. 43, 904–914. 10.1016/j.molcel.2011.08.018 21925379 PMC3199020

[B146] Wang J. Pan J. Li H. Long J. Fang F. Chen J. (2018). LncRNA ZEB1-AS1 is suppressed by p53 to attenuate renal fibrosis in diabetic nephropathy. Mol. Ther. Nucleic Acids 12, 741–750. 10.1016/j.omtn.2018.07.012 30121551 PMC6095953

[B147] Wang J. J. Wang X. Song Y. X. Zhao J. H. Sun J. X. Shi J. X. (2019a). Circulating noncoding RNAs have a promising future acting as novel biomarkers for colorectal cancer. Dis. Markers 2019, 2587109. 10.1155/2019/2587109 31275444 PMC6589288

[B148] Wang Y. Wu S. Yang X. Li X. Chen R. (2019b). Association between polymorphism in the promoter region of lncRNA GAS5 and the risk of colorectal cancer. Biosci. Rep. 39 (4), BSR20190091. 10.1042/BSR20190091 30902880 PMC6465203

[B149] Wang X. Shen C. Zhu J. Shen G. Li Z. Dong J. (2019c). Long noncoding RNAs in the regulation of oxidative stress. Oxid. Med. Cell. Longev. 2019, 1318795. 10.1155/2019/1318795 30911342 PMC6398004

[B150] Wang X. Yang P. Zhang D. Lu M. Zhang C. Sun Y. (2021). LncRNA SNHG14 promotes cell proliferation and invasion in colorectal cancer by modulating the miR-519b-3p/DDX5 axis. J. Cancer 12 (16), 4958–4970. 10.7150/jca.55495 34234865 PMC8247390

[B151] Wang J. Liu B. Cao J. Zhao L. Wang G. (2022). MIR31HG expression predicts poor prognosis and promotes colorectal cancer progression. Cancer Manag. Res. 14, 1973–1986. 10.2147/CMAR.S351928 35733512 PMC9208482

[B152] Wang K. Lu Y. Li H. Zhang J. Ju Y. Ouyang M. (2024a). Role of long non-coding RNAs in metabolic reprogramming of gastrointestinal cancer cells. Cancer Cell. Int. 24 (1), 15. 10.1186/s12935-023-03194-0 38184562 PMC10770979

[B153] Wang Y. Wang P. Wang Q. Chen S. Wang X. Zhong X. (2024b). The long noncoding RNA HNF1A-AS1 with dual functions in the regulation of cytochrome P450 3A4. Biochem. Pharmacol. 220, 116016. 10.1016/j.bcp.2023.116016 38176619

[B154] Wang M. Zheng C. Wang Z. Li R. Zhang W. Zhong Y. (2025). Colorectal cancer: highlight the clinical research current progress. Holist. Integr. Oncol. 4, 17. 10.1007/s44178-025-00152-w

[B155] Wen L. Tan C. Ma S. Li X. (2022). LncRNAs: key regulators of signaling pathways in tumor glycolysis. Dis. Markers 2022, 2267963. 10.1155/2022/2267963 36124026 PMC9482549

[B156] Wu J. Meng X. Jia Y. Chai J. Wang J. Xue X. (2020a). Long non-coding RNA HNF1A-AS1 upregulates OTX1 to enhance angiogenesis in colon cancer *via* binding of transcription factor PBX3. Exp. Cell. Res. 393 (2), 112025. 10.1016/j.yexcr.2020.112025 32325080

[B157] Wu G. Xue M. Zhao Y. Han Y. Li C. Zhang S. (2020b). Long noncoding RNA ZEB1-AS1 acts as a sponge of miR-141-3p to inhibit cell proliferation in colorectal cancer. Int. J. Med. Sci. 17 (11), 1589–1597. 10.7150/ijms.46698 32669962 PMC7359398

[B158] Wu J. Lyu R. Chen S. Wang X. (2023). Long noncoding RNA zinc finger E-box binding homeobox 1 antisense RNA 1 regulates myocardial fibrosis in diabetes through the Hippo–Yes-associated protein signaling pathway. J. Diabetes Investig. 14 (8), 940–952. 10.1111/jdi.13989 37309277 PMC10360388

[B159] Xia Y. Shen S. Verma I. M. (2014). NF-κB, an active player in human cancers. Cancer Immunol. Res. 2 (9), 823–830. 10.1158/2326-6066.CIR-14-0112 25187272 PMC4155602

[B160] Xia F. Wang Y. Xue M. Zhu L. Jia D. Shi Y. (2022). LncRNA KCNQ1OT1: molecular mechanisms and pathogenic roles in human diseases. Genes. Dis. 9 (6), 1556–1565. 10.1016/j.gendis.2021.07.003 36157505 PMC9485204

[B161] Xian D. Niu L. Zeng J. Wang L. (2021). LncRNA KCNQ1OT1 secreted by tumor cell-derived exosomes mediates immune escape in colorectal cancer by regulating PD-L1 ubiquitination *via* miR-30a-5p/USP22. Front. Cell. Dev. Biol. 9, 653808. 10.3389/fcell.2021.653808 34350172 PMC8326752

[B162] Xu Y. Qiu M. Shen M. Dong S. Ye G. Shi X. (2021). The emerging regulatory roles of long non-coding RNAs implicated in cancer metabolism. Mol. Ther. 29 (7), 2209–2218. 10.1016/j.ymthe.2021.03.017 33775912 PMC8261164

[B163] Xu W. W. Jin J. Wu X. Y. Ren Q. L. Farzaneh M. (2022). MALAT1-related signaling pathways in colorectal cancer. Cancer Cell. Int. 22 (1), 126. 10.1186/s12935-022-02540-y 35305641 PMC8933897

[B164] Yan L. J. (2014). Pathogenesis of chronic hyperglycemia: from reductive stress to oxidative stress. J. Diabetes Res. 2014, 137919. 10.1155/2014/137919 25019091 PMC4082845

[B165] Yan C. Chen J. Chen N. (2016). Long noncoding RNA MALAT1 promotes hepatic steatosis and insulin resistance by increasing nuclear SREBP-1c protein stability. Sci. Rep. 6, 22640. 10.1038/srep22640 26935028 PMC4776244

[B166] Yan H. Yao P. Hu K. Li X. Li H. (2021). Long non-coding RNA urothelial carcinoma-associated 1 promotes high glucose-induced human retinal endothelial cell angiogenesis through regulating microRNA-624-3p/vascular endothelial growth factor C. J. Diabetes Investig. 12 (11), 1948–1957. 10.1111/jdi.13617 34137197 PMC8565426

[B167] Yang F. Qin Y. Lv J. Wang Y. Che H. Chen X. (2018). Silencing long non-coding RNA Kcnq1ot1 alleviates pyroptosis and fibrosis in diabetic cardiomyopathy. Cell. Death Dis. 9 (10), 1000. 10.1038/s41419-018-1029-4 30250027 PMC6155223

[B168] Yang Y. Deng X. Li Q. Wang F. Miao L. Jiang Q. (2020). Emerging roles of long noncoding RNAs in cholangiocarcinoma: advances and challenges. Cancer Commun. (Lond). 40 (12), 655–680. 10.1002/cac2.12109 33142045 PMC7743012

[B169] Yang J. Qi M. Fei X. Wang X. Wang K. (2021). LncRNA H19: a novel oncogene in multiple cancers. Int. J. Biol. Sci. 17 (12), 3188–3208. 10.7150/ijbs.62573 34421359 PMC8375239

[B170] Yang Z. Song D. Wang Y. Tang L. (2022). LncRNA MALAT1 promotes diabetic nephropathy progression *via* miR-15b-5p/TLR4 signaling axis. J. Immunol. Res. 2022:8098001 2022, 1–13. 10.1155/2022/8098001 PMC9334040 35910856

[B171] Yang Q. Fang D. Chen J. Hu S. Chen N. Jiang J. (2023a). LncRNAs associated with oxidative stress in diabetic wound healing: regulatory mechanisms and application prospects. Theranostics 13 (11), 3655–3674. 10.7150/thno.85823 37441585 PMC10334824

[B172] Yang X. Du Y. Luo L. Xu X. Xiong S. Yang X. (2023b). Deciphering the enigmatic influence: non-coding RNAs orchestrating the Wnt/β-catenin signaling pathway in tumor progression. Int. J. Mol. Sci. 24 (18), 13909. 10.3390/ijms241813909 37762212 PMC10530696

[B173] Yi W. Miller J. R. Hu G. Adjeroh D. A. (2025). LncRNA subcellular localization across diverse cell lines: an exploration using deep learning with inexact q-mers. Noncoding RNA. 11 (4), 49. 10.3390/ncrna11040049 40700092 PMC12286058

[B174] Yu G. H. Li S. F. Wei R. Jiang Z. (2022). Diabetes and colorectal cancer risk: clinical and therapeutic implications. J. Diabetes Res. 2022, 1747326. 10.1155/2022/1747326 35296101 PMC8920658

[B175] Yu J. Qin M. Li J. Cui S. (2023). LncRNA SNHG4 sponges miR-200b to inhibit cell apoptosis in diabetic retinopathy. Arch. Physiol. Biochem. 129 (5), 1117–1122. 10.1080/13813455.2021.1900873 33822671

[B176] Yuan H. Tang H. Shi L. (2022). Low expression of lncRNA UCA1 assists the diagnosis of type 2 diabetes mellitus and predicts an increased risk of cardiovascular complications. All Life. 15, 1315–1324. 10.1080/26895293.2022.2138561

[B177] Zeinelabdeen Y. Abaza T. Yasser M. B. Elemam N. M. Youness R. A. (2024). MIAT lncRNA: a multifunctional key player in non-oncological pathological conditions. Noncoding RNA Res. 9 (2), 447–462. 10.1016/j.ncrna.2024.01.011 38511054 PMC10950597

[B178] Zeng J. Xiao X. Q. Zhou Z. Y. (2022). A hypoxia-induced SCF^FBXL1 E3 ligase ubiquitinates and degrades the MEN1 tumor suppressor to promote colorectal cancer tumorigenesis. Cancer Res. Treat. 54 (2), 525–540. 10.4143/crt.2021.373 34237211 PMC9016320

[B179] Zhan K. Pan H. Zhou Z. Tang W. Ye Z. Huang S. (2023). Biological role of long non-coding RNA KCNQ1OT1 in cancer progression. Biomed. Pharmacother. 169, 115876. 10.1016/j.biopha.2023.115876 37976888

[B180] Zhang Y. Wang X. (2020). Targeting the Wnt/β-catenin signaling pathway in cancer. J. Hematol. Oncol. 13 (1), 165. 10.1186/s13045-020-00990-3 33276800 PMC7716495

[B181] Zhang C. Wang Y. (2022). KCNQ1OT1 polymorphism rs35622507 and methylation status of KCNQ1OT1 promoter influence the drug resistance to L-OHP. Aging (Albany NY) 14 (4), 1836–1847. 10.18632/aging.203906 35193115 PMC8908920

[B182] Zhang J. Chen M. Chen J. Lin S. Cai D. Chen C. (2017). Long non-coding RNA MIAT acts as a biomarker in diabetic retinopathy by absorbing miR-29b and regulating cell apoptosis. Biosci. Rep. 37 (2), BSR20170036. 10.1042/BSR20170036 28246353 PMC5408653

[B183] Zhang N. Geng T. Wang Z. Zhang R. Cao T. Camporez J. P. (2018). Elevated hepatic expression of H19 long noncoding RNA contributes to diabetic hyperglycemia. JCI Insight 3 (10), e120304. 10.1172/jci.insight.120304 29769440 PMC6012507

[B184] Zhang Y. Chang B. Zhang J. Wu X. (2019). LncRNA SOX2OT alleviates high glucose-induced podocyte injury through autophagy induction *via* the miR-9/SIRT1 axis. Exp. Mol. Pathol. 110, 104283. 10.1016/j.yexmp.2019.104283 31301307

[B185] Zhang Y. Shi J. Luo J. Liu C. Zhu L. (2022). Regulatory mechanisms and potential medical applications of HNF1A-AS1 in cancers. Am. J. Transl. Res. 14 (6), 4154–4168. 10.18632/aging.203906 35836869 PMC9274608

[B186] Zhang D. Pei S. Feng Z. Xia G. (2025a). Functions and mechanisms of lncRNAs in immune escape and their application in immunotherapy for colorectal cancer. J. Transl. Med. 23, 689. 10.1186/s12967-024-05421-y 40537762 PMC12180200

[B187] Zhang X. Shi L. Xing M. Li C. Ma F. Ma Y. (2025b). Interplay between lncRNAs and the PI3K/AKT signaling pathway in the progression of digestive system neoplasms (review). Int. J. Mol. Med. 55 (1), 15. 10.3892/ijmm.2024.5456 39513614 PMC11573320

[B188] Zhao Y. P. Liu J. L. Wang S. Li X. (2025). Role of non-coding RNA-regulated ferroptosis in colorectal cancer. Cell. Death Discov. 11, 315. 10.1038/s41420-025-02606-6 40628711 PMC12238284

[B189] Zhao S. Zhang X. Chen S. Zhang S. (2021a). Long noncoding RNAs: fine-tuners hidden in the cancer signaling network. Cell. Death Discov. 7 (1), 283. 10.1038/s41420-021-00678-8 34635646 PMC8505617

[B190] Zhao M. Wang H. Chen J. Xi Y. Wang F. Huo C. (2021b). Expression of long non-coding RNA H19 in colorectal cancer patients with type 2 diabetes. Arch. Physiol. Biochem. 127 (3), 228–234. 10.1080/13813455.2019.1628068 31232113

[B191] Zhao L. Chen H. Wu L. Li Z. Zhang R. Zeng Y. (2021c). LncRNA KCNQ1OT1 promotes the development of diabetic nephropathy by regulating miR-93-5p/ROCK2 axis. Diabetol. Metab. Syndr. 13 (1), 108. 10.1186/s13098-021-00726-4 34654473 PMC8518197

[B192] Zhao Y. Ye X. Xiong Z. Ihsan A. Ares I. Martínez M. (2023). Cancer metabolism: the role of ROS in DNA damage and induction of apoptosis in cancer cells. Metabolites. 27;(7):796. 10.3390/metabo13070796 PMC10383295 37512503

[B193] Zheng J. Ludin A. F. M. Rajab N. F. Shaolong L. Jufri N. F. (2025). The roles of lncMALAT1 in coronary artery disease regulation and therapeutic perspective: a systematic review. iScience 28 (7), 112945. 10.1016/j.isci.2025.112945 40687804 PMC12271600

[B194] Zhou Z. Zhang P. Hu X. Kim J. Yao F. Xiao Z. (2017). USP51 promotes deubiquitination and stabilization of ZEB1. Am. J. Cancer Res. 7 (10), 2020–2031. 29119051 PMC5665849

[B195] Zhou Z. Tan F. Pei Q. Li C. Zhou Y. Li Y. (2021). LncRNA SNHG4 modulates colorectal cancer cell cycle and cell proliferation through regulating miR-590-3p/CDK1 axis. Aging (Albany NY) 13 (7), 9838–9858. 10.18632/aging.202737 33744866 PMC8064176

[B196] Zhou H. Lv X. Liang Y. Yu D. Wang W. (2026). Epigenetic regulation in a high-sugar environment. Int. J. Mol. Med. 57 (5), 131. 10.3892/ijmm.2026.5802 41860052 PMC13034894

[B197] Zhu S. Chen C. Y. Hao Y. (2021). LncRNA KCNQ1OT1 acts as miR-216b-5p sponge to promote colorectal cancer progression *via* up-regulating ZNF146. J. Mol. Histol. 52 (3), 479–490. 10.1007/s10735-020-09942-0 33394291

[B198] Zichittella C. Loria M. Celesia A. Di Liberto D. Corrado C. Alessandro R. (2023). Long non-coding RNA H19 enhances the pro-apoptotic activity of ITF2357 (a histone deacetylase inhibitor) in colorectal cancer cells. Front. Pharmacol. 14, 1275833. 10.3389/fphar.2023.1275833 37841928 PMC10572549

